# Covalent Allosteric Inhibitors of Akt Generated Using a Click Fragment Approach

**DOI:** 10.1002/cmdc.202100776

**Published:** 2022-03-09

**Authors:** Leandi van der Westhuizen, Jörn Weisner, Abu Taher, Ina Landel, Lena Quambusch, Marius Lindemann, Niklas Uhlenbrock, Matthias P. Müller, Ivan R. Green, Stephen C. Pelly, Daniel Rauh, Willem A. L. van Otterlo

**Affiliations:** ^1^ Department of Chemistry and Polymer Science Stellenbosch University Matieland 7602 South Africa; ^2^ Faculty of Chemistry and Chemical Biology TU Dortmund University Otto-Hahn-Strasse 4a 44227 Dortmund Germany; ^3^ Drug Discovery Hub Dortmund (DDHD) am Zentrum für Integrierte Wirkstoffforschung (ZIW) 44227 Dortmund Germany; ^4^ Department of Chemistry Emory University 1515 Dickey Drive Atlanta GA 30322 USA

**Keywords:** Akt kinase, covalent allosteric inhibitors, imidazopyridines, click chemistry, fragments

## Abstract

Akt is a protein kinase that has been implicated in the progression of cancerous tumours. A number of covalent allosteric Akt inhibitors are known, and based on these scaffolds, a small library of novel potential covalent allosteric imidazopyridine‐based inhibitors was designed. The envisaged compounds were synthesised, with click chemistry enabling a modular approach to a number of the target compounds. The binding modes, potencies and antiproliferative activities of these synthesised compounds were explored, thereby furthering the structure activity relationship knowledge of this class of Akt inhibitors. Three novel covalent inhibitors were identified, exhibiting moderate activity against Akt1 and various cancer cell lines, potentially paving the way for future covalent allosteric inhibitors with improved properties.

## Graphical abstract

**A small library** of potential covalent allosteric imidazopyridine‐based inhibitors was designed and synthesised, with click chemistry enabling a modular approach. The binding modes, potencies and antiproliferative activities of these compounds was subsequently explored. Three novel covalent inhibitors were identified, exhibiting moderate activity against Akt1 and various cancer cell lines, potentially paving the way for future covalent allosteric inhibitors with improved properties.

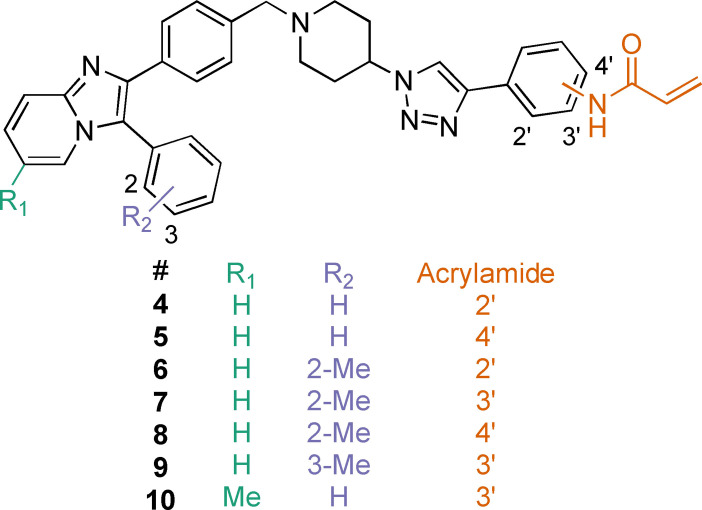

## Introduction

Akt is a serine/threonine kinase of the phosphatidylinositol 3‐kinase/Akt/mammalian target of rapamycin (PI3K/Akt/mTOR) signalling pathway and is involved in regulating several cellular processes including proliferation, growth, cellular survival, glucose metabolism, angiogenesis and migration.^[1]^ This kinase has three wild‐type mammalian isoforms, namely Akt1, Akt2 and Akt3.^[2]^ These three isoforms share a conserved structure, each consisting of an amino‐terminal pleckstrin homology (PH) domain, a central catalytic kinase domain and a short carboxy‐terminal regulatory tail.^[3]^ All three Akt isoforms have been implicated in the progression of tumours and activated Akt has been linked to reduced patient survival and resistance to conventional cancer therapies.^[4]^ Akt inhibition sensitises cancer cells to DNA damage by other therapies and it is therefore a target as part of combination therapy.^[5]^

**Figure 1:**
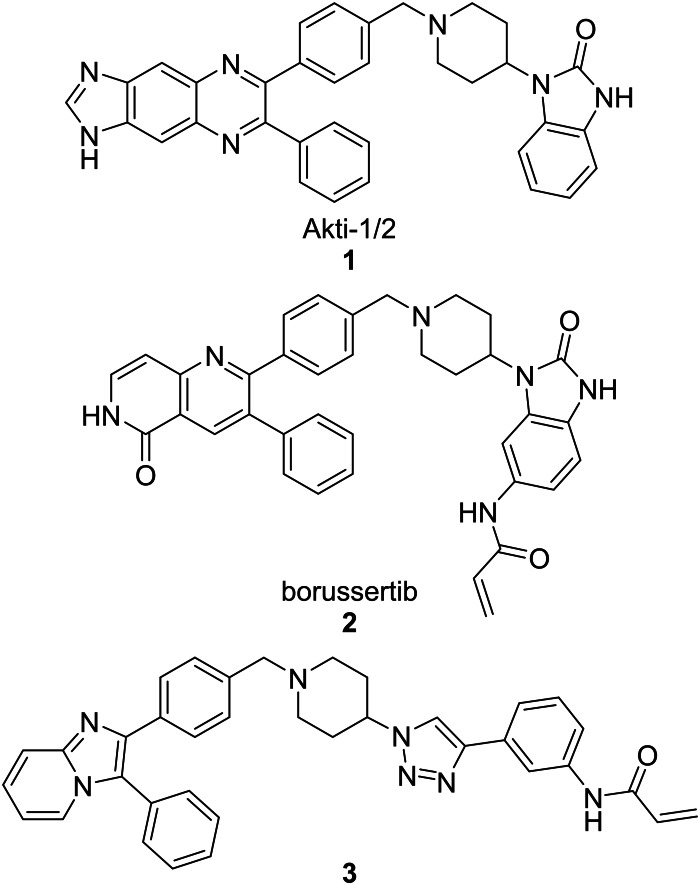
Allosteric Akt inhibitor Akti‐1/2 (**1**) and covalent allosteric inhibitors of Akt, borussertib (**2**) and triazole **3**.

The allosteric Akt inhibitor, Akti‐1/2 (**1**) (Figure 1) served as inspiration for the design and synthesis of covalent allosteric Akt inhibitors borussertib (**2**) and **3**.^[6]^ There has been a growing interest in allosteric kinase inhibitors, with covalent inhibitors also imparting their own advantages, hence combining the two types.^[7]^ This is further supported by recent literature, looking at both covalent and allosteric inhibitors, as well as combining the two.^[8]^

Inhibitors **1**–**3** bind in the allosteric pocket at the interface between the kinase and PH domains, with inhibitors **2** and **3** also being covalent modifiers of Akt, irreversibly binding to Cys296 and Cys310.[^6^, ^9^] Additionally, the activities of inhibitors **2** and **3** were compared to the activities of their respective reversible counterparts and their covalent nature was found to increase their potency.^[6a]^ These two covalent allosteric Akt inhibitors served as inspiration for the design and synthesis of further potential covalent allosteric inhibitors of Akt, conveniently accessible through a modular approach with click chemistry, as described in this paper.

## Results and Discussion

### Design

**Figure 2:**
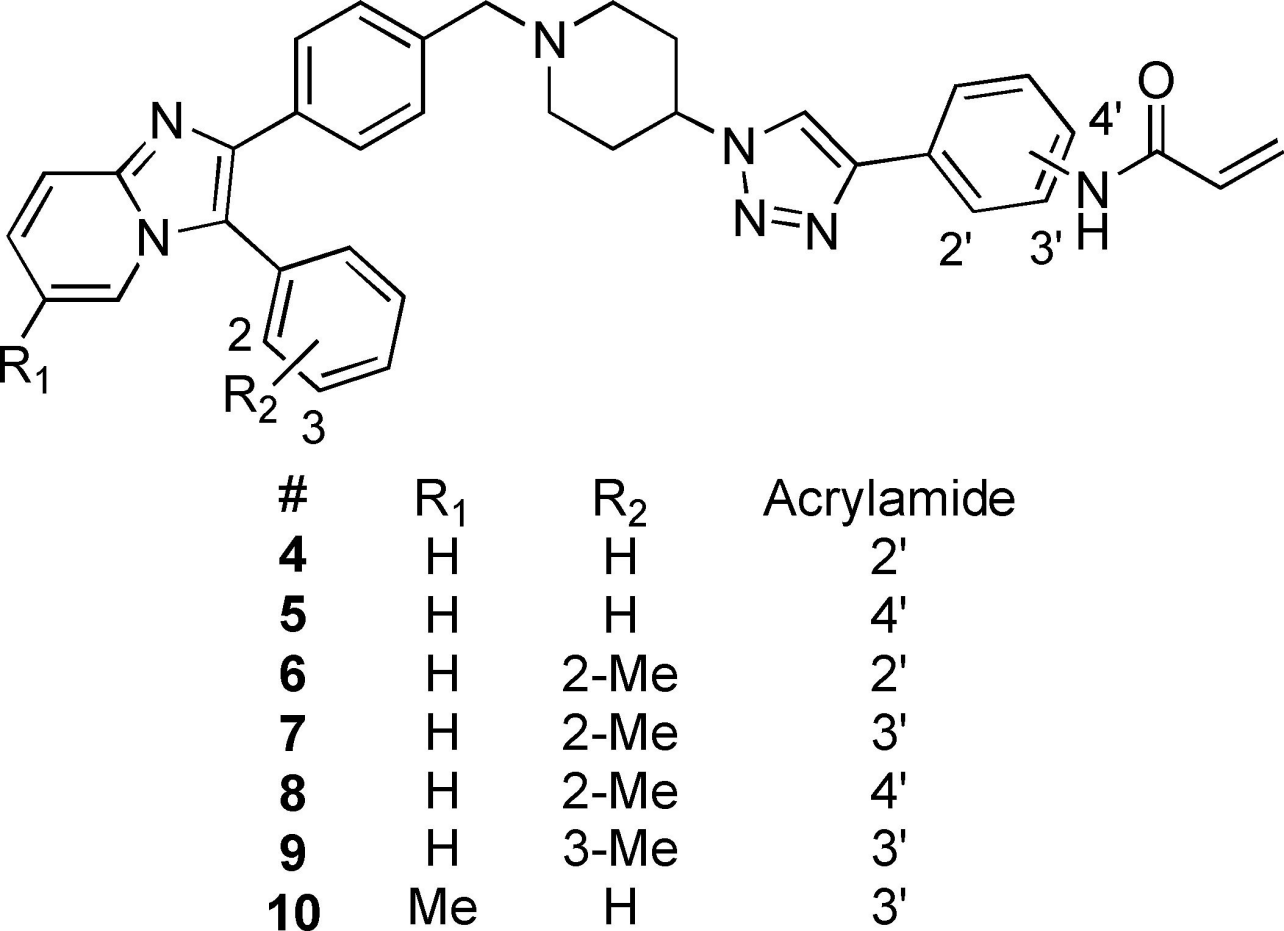
The 1,2,3‐triazole‐containing potential Akt inhibitors.

Overall, three groups of potential covalent allosteric Akt inhibitors were designed. The first group consisted of three regioisomeric analogues, namely the previously synthesised and evaluated *meta*‐acrylamide **3** and its *ortho*‐ and *para*‐acrylamide counterparts **4** and **5**, respectively (Figure 2).^[6a]^ Previous research showed that the *meta*‐acrylamide **3** was able to bind both the Akt Cys296 and Cys310 residues.^[6a]^ Synthesis and evaluation of **4** and **5** was required to determine the best position of the acrylamide in the three regioisomers to target these two cysteine residues.

**Figure 3:**
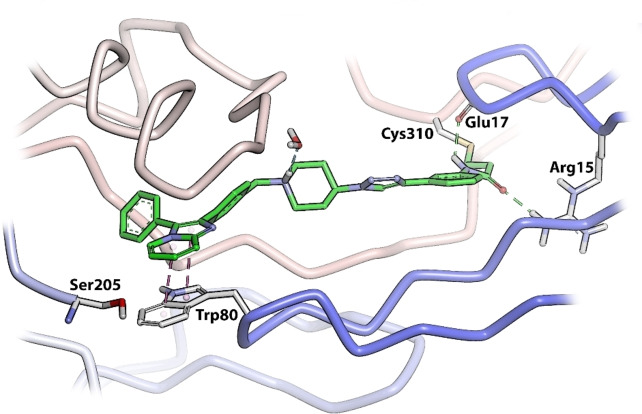
Compound **3** covalently docked into the allosteric pocket of Akt (receptor from PDB 6HHG).

The second group of target compounds consisted of methylated versions of **3**–**5**, namely **6**–**10** (Figure 2). The “methyl effect” has been successfully used in the rational design of bioactive compounds and drugs in an effort to improve the biological activity and physical properties of a molecule.^[10]^ Inhibitor **3** had poor enzymatic potency and rather low solubility and introduction of a methyl group was therefore chosen as a potential way to improve these properties. An *ortho*‐methyl in a biaryl system has been shown to increase the interphenyl dihedral angle, with a consequent decrease in molecular planarity.^[11]^ This decrease in planarity can potentially decrease the efficiency in crystal packing due to reduced π‐π stacking and therefore has the potential to increase aqueous solubility.^[11b]^ We therefore decided to incorporate an *ortho*‐methyl in the phenyl‐imidazo biaryl system on the left‐hand side of the molecule series, thereby providing us with regioisomers **6**–**8**. For comparison purposes, the *meta*‐methyl **9** (with a *meta*‐acrylamide warhead) was also selected as a synthetic target, to evaluate our hypothesis. Furthermore, preliminary molecular modelling suggested that selectivity for Akt1 could be achieved by the introduction of yet another methyl group on the imidazopyridine scaffold of **3**. Covalent docking of **3** into Akt (PDB 6HHG)^[12]^ indicated that this part of **3** occupies the same space as the imidazoquinoxaline part of **1** (from PDB 3O96),^[6b]^ in the region of Ser205 (Figure 3). However, Akt2 and Akt3 have a more sterically demanding Thr207 in this location, potentially providing us with an opportunity to introduce selectivity by the addition of a judiciously placed methyl group on the phenyl of the imidazoquinoxaline moiety of **3**. As with *meta*‐methyl **9**, only the *meta*‐acrylamide regioisomer was selected as synthetic target, and therefore the synthesis of methyl‐imidazopyridine **10** was envisaged.

**Figure 4:**
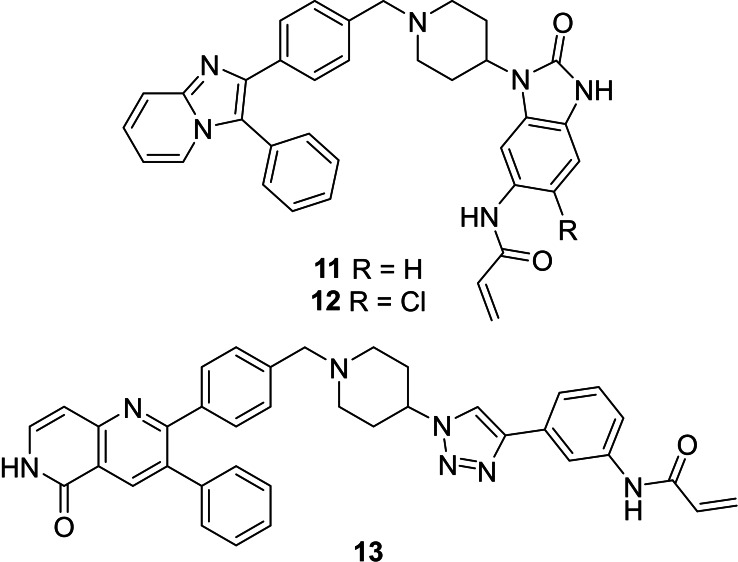
The hybrid potential Akt inhibitors derived from covalent allosteric inhibitors **2** and **3**.

The third and final group of target compounds included hybrid structures based on **2** and **3**, thereby producing **11** and **13** (Figure 4). Since **3** had displayed much lower potency than borussertib (**2**), these compounds were seen as valuable additions for SAR studies, with their evaluation hoped to enable the identification of which inhibitor portions imparted the best activity against Akt. Additionally, **12**, the chlorinated counterpart of **11** was also added as a potential synthetic target (Figure 3). This compound was easily accessible during the synthesis towards **11** and therefore included for comparison purposes. Moreover, the chlorinated analogue of borussertib (**2**) was previously demonstrated to retain the high inhibitory potency of its parent compound and exhibited moderately superior antiproliferative activities in cancer cell lines harbouring genetic alterations in the PI3K/Akt/mTOR signalling pathway.^[12]^

**Chemistry**. For the first two groups of compounds, **3**–**10**, the synthesis of the representative compound **4** will be discussed, the general synthetic approach of which is closely related to **3**, as described by Weisner *et al*.^[6a]^ Please note that during the synthesis of the molecules, the imidazopyridine part will be referred to as the western portion, with the acrylamide‐bearing part being called the eastern portion.

**Scheme 1:**
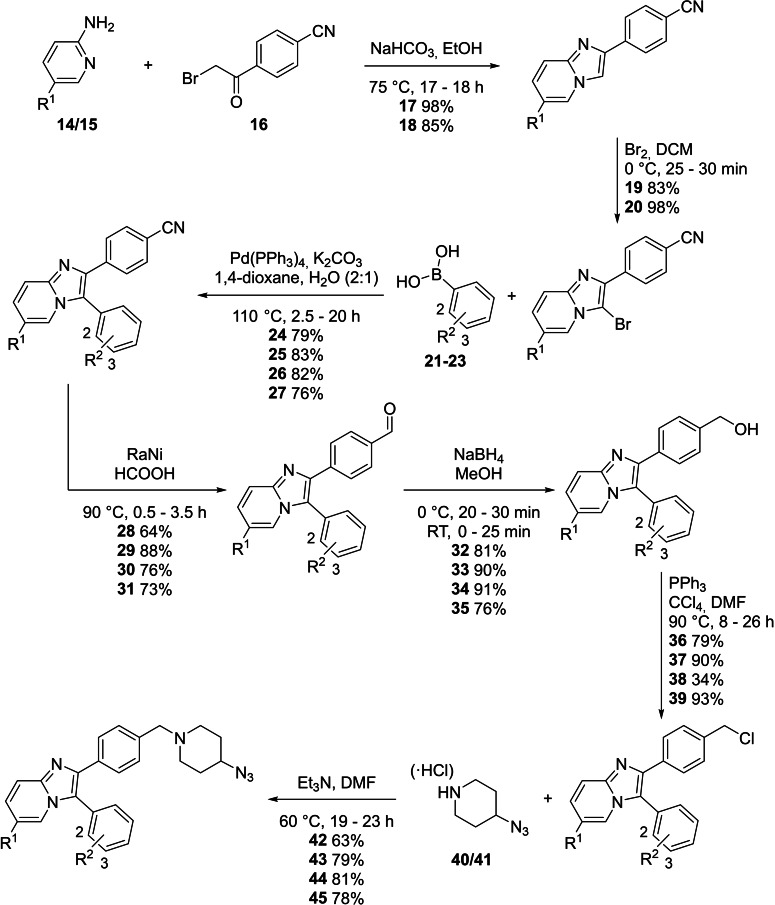
The synthesis of western azide intermediates **42**, **43**, **44** and **45**. For **42**, R^1^, R^2^=H, for **43**, R^1^=H, R^2^=2‐Me, for **44**, R^1^=H, R^2^=3‐Me and for **45**, R^1^=Me, R^2^=H.

**Scheme 2:**
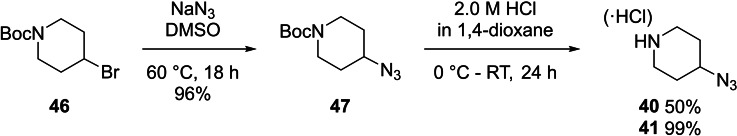
The synthesis of piperidine **40** and **41**, used for the synthesis of intermediates **42**, **43**, **44** and **45** and target compound **13**.

Synthesis of imidazopyridine **17** (and **18**) was achieved from the reaction of commercially available 2‐aminopyridine **14** and 2‐bromo‐4’‐cyanoacetophenone **16** (or 5‐methylpyridin‐2‐amine **15** and 2‐bromo‐4’‐cyanoacetophenone **16** for **18**).^[13]^ Bromination of **17** (and **18**) using molecular bromine then afforded **19** (and **20**) as shown in Scheme 1.^[14]^ A Suzuki‐Miyaura coupling reaction was done between **19** (and **20**) and phenylboronic acid **21** to afford **24** (and **27**).^[15]^ Similarly, the coupling reaction between **19** and *o*‐tolylboronic acid **22** or *m*‐tolylboronic acid **23** afforded **25** and **26**. Raney nickel reduction of the nitrile functionality of **24** (and **25**, **26**, **27**) afforded the benzaldehyde **28** (and **29**, **30**, **31**).^[14]^ Further reduction of the benzaldehyde with sodium borohydride afforded **32** (and **33**, **34**, **35**) in good yields.^[16]^ An Appel reaction with benzyl alcohols **32**, **33**, **34** and **35** next afforded the benzyl chlorides **36**,**37**, **38** and **39**.^[17]^ Lastly, nucleophilic substitution between the benzyl chloride **36** (and **37**, **38**, **39**) and piperidines **40** or **41** (their synthesis is discussed below, Scheme 2) afforded western azide intermediate(s) **42** (and **43**, **44**, **45**).^[18]^

The piperidines **40** and **41** were used for the synthesis of the four intermediates above (and in the subsequent synthesis of **13**). These compounds were synthesised as shown in Scheme 2, starting with nucleophilic substitution between commercially available *tert*‐butyl 4‐bromopiperidine‐1‐carboxylate **46** and sodium azide to afford **47**.^[19]^ Subsequent deprotection with hydrochloric acid in 1,4‐dioxane afforded piperidine **41** as the hydrochloride salt, which could be neutralised to afford piperidine **40**.^[20]^ The poor yield of piperidine **40** is partially attributable to its highly polar nature, which resulted in tailing during purification by column chromatography, and the challenge of its visualization on TLC, requiring ninhydrin stain.

**Scheme 3:**
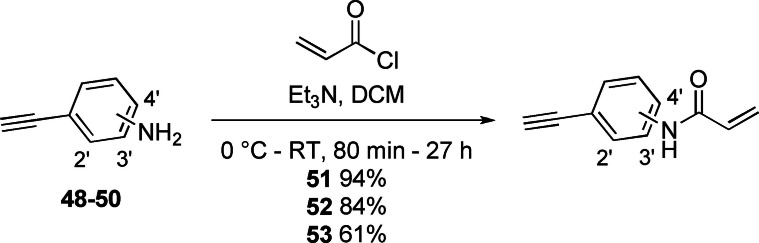
The synthesis of the three acrylamide warhead‐containing alkynes **51**–**53**. For **51**, acrylamide=2’, for **52**, acrylamide=3’ and for **53**, acrylamide=4’.

Acrylamides **51**–**53** were synthesised from the corresponding anilines and acryloyl chloride (Scheme 3) and used for the synthesis of compounds **3**–**10** and **13**.^[21]^

**Scheme 4:**
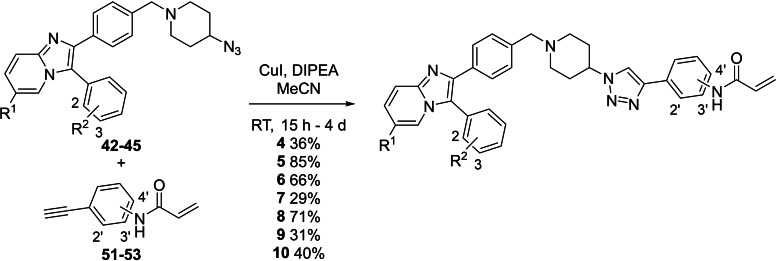
The synthesis of compounds **4**–**10** using CuAAC between western azide intermediates **42**, **43**, **44** and **45** and acrylamides **51**–**53**. For **4**, R^1^, R^2^=H, acrylamide=2’, for **3**, R^1^, R^2^=H, acrylamide=3’, for **5**, R^1^, R^2^=H, acrylamide=4’, for **6**, R^1^=H, R^2^=2‐Me, acrylamide=2’, for **7**, R^1^=H, R^2^=2‐Me, acrylamide=3’, for **8**, R^1^=H, R^2^=2‐Me, acrylamide=4’, for **9**, R^1^=H, R^2^=3‐Me, acrylamide=3’ and for **10**, R^1^=Me, R^2^=H, acrylamide=3’.

Copper(I)‐catalysed azide alkyne cycloaddition (CuAAC) or ‘click’ chemistry was next employed for the synthesis of compounds **4**–**10**, using the appropriate combinations of western azide intermediates **42**, **43**, **44** and **45** and acrylamides **51**–**53** (Scheme 4).^[22]^

**Scheme 5:**
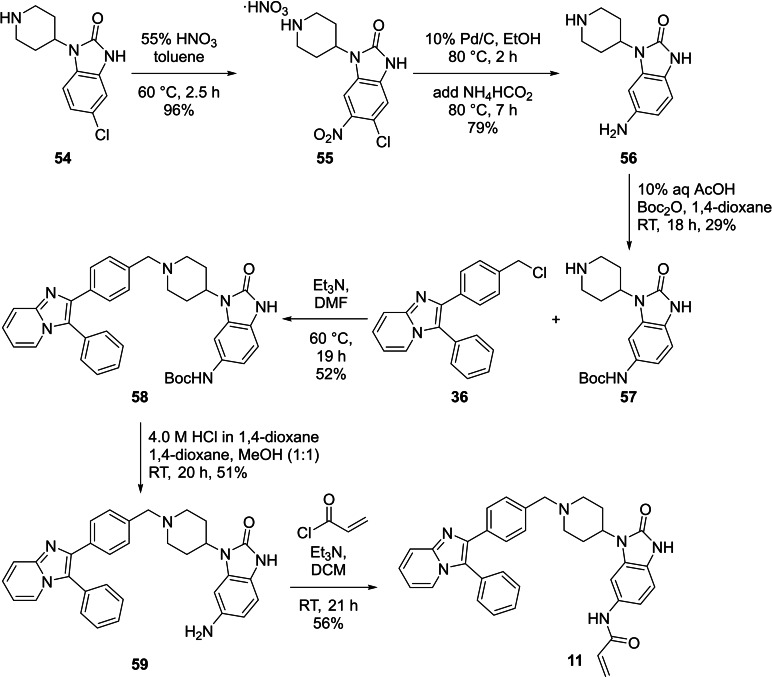
The synthesis of hybrid **11**.

A number of the reactions for the synthesis of hybrid **11** followed procedures reported by Weisner *et al*. (Scheme 5).^[6a]^ The synthesis started with the nitration of commercially available 5‐chloro‐1‐(piperidine‐4‐yl)‐1,3‐dihydro‐2*H*‐benzo[*d*]imidazol‐2‐one **54** to afford **55**.^[23]^ Simultaneous reduction and hydrodehalogenation of **55** then afforded **56**.^[24]^ This was followed by selective Boc‐protection to afford **57** in a low yield of 29 %, in part due to its solubility in water which led to loss of material during work‐up.^[25]^ Nucleophilic substitution between **57** and the previously‐synthesised **36** then gave **58**. Boc‐deprotection of **58** afforded **59**, after which attachment of the acrylamide warhead with acryloyl chloride gave the target **11**.

**Scheme 6:**
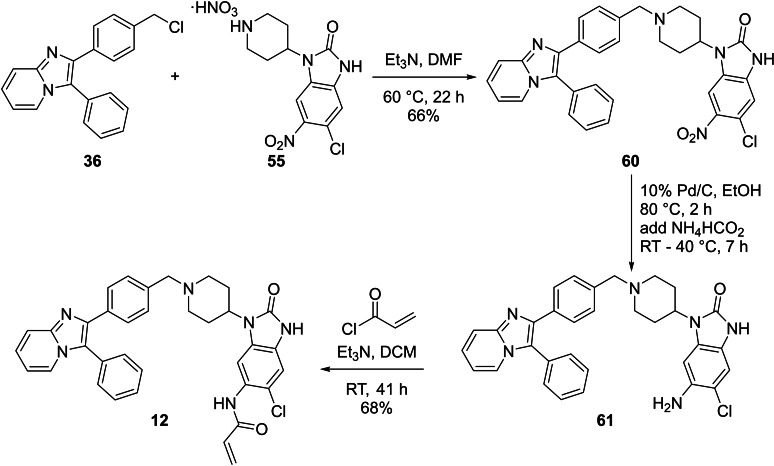
The synthesis of compound **12**.

Compound **12**, the chlorinated version of **11** was obtained during the synthesis towards **11** and its synthesis is given in Scheme 6. The synthesis started with nucleophilic substitution between **36** and **55** to afford **60**. Attempted simultaneous reduction and hydrodehalogenation conditions afforded **61**, with the chlorine still present. Attachment of the acrylamide warhead to **61** then afforded **12** in reasonable yield.

**Scheme 7:**
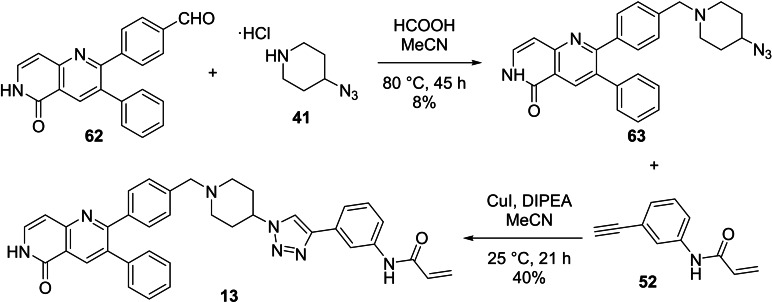
The synthesis of second hybrid **13**.

Lastly, the synthesis of the second hybrid **13** is given in Scheme 7. Aldehyde **62** was supplied, synthesised as described by Uhlenbrock *et al*.^[12]^ A Leuckart‐Wallach reaction between **62** and **41** then afforded the western azide intermediate **63** in a low yield, as also seen in the literature.^[12]^ The reason for this particular approach was that owing to the poor reaction yields obtained for similar scaffolds in previous research performed in our groups,^[6a]^ conventional reductive amination with sodium triacetoxyborohydride or sodium cyanoborohydride was not attempted. The low yield obtained with the Leuckart‐Wallach reaction is thought to be from a combination of **62** being a poor electrophile (possible resonance structures) and **41** being a mediocre nucleophile, either due to electronic or steric effects, or both. A CuAAC reaction between **63** and **52** then afforded the hybrid compound **13**.

### Biological evaluation

A number of different techniques and assays were utilised to evaluate the synthesised compounds. These were done as described in the Supporting Information.

#### Identification of covalent allosteric binders

Covalent binders of Akt1 were identified with LC‐MS. Test compounds were in turn co‐incubated with Akt1 and then analysed by LC‐MS. A mass increase equivalent to the mass of the compound indicated covalent binding, whereas no increase in the mass observed was indicative of reversible Akt binding or of a compound that did not bind Akt at all.

From the LC‐MS results (Figure S1), **11**, **12** and **13** were identified as novel covalent binders of Akt1. As stated earlier, **3** was previously identified as a covalent binder of Akt1. Moreover, **4** and **10** were identified as partial covalent binders of Akt1, potentially due to their low activity against Akt1. This was to be confirmed by the results below.

To further corroborate the covalent allosteric binding mode, we set out to co‐crystallise Akt1 in complex with the newly synthesised inhibitors. Although we were able to obtain co‐crystals of Akt1 complexed with compound **13**, they unfortunately only resulted in intergrown crystals with low quality diffraction data from multiple lattices and a moderate resolution of 3.0 Å. Nevertheless, since several attempts to improve crystal quality were not successful, a small part of the diffraction data was processed. In line with published co‐crystal structures of Akt1 in complex with covalent allosteric Akt inhibitors, the resulting electron density map indicates binding of **13** in the interdomain pocket between the PH and the kinase domain (Figure S2).[^9^, ^12^] However, due to insufficient data quality, the structure was not submitted to the PDB.

#### Biochemical evaluation

The homogeneous time‐resolved fluorescence (HTRF®) KinEASE™ assay by Cisbio was used to determine the half‐maximal inhibitory concentration (IC_50_) values of the synthesised compounds against Akt1, Akt2 and Akt3 (Table 1).^[26]^ The amino acid differences between Akt1 and the other two wild‐type isoforms are expected to change the allosteric binding pocket character and dimensions, influencing the binding of this class of allosteric inhibitors.[^6b^, ^27^] This is supported by inhibitor **1** being less active against the latter two Akt forms than against Akt1.^[6b]^

**Table 1:** IC_50_ values of the test compounds determined against a number of different forms of Akt.^[a]^

Compd	Structure	Akt1 [nM]	Akt2 [nM]	Akt3 [nM]
**4**	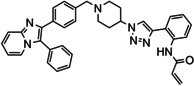	925±277	>20000	>20000
**3**	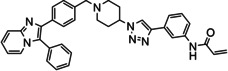	372±49	>20000	>20000
**5**	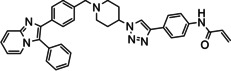	2876±1730	>20000	>20000
**6**	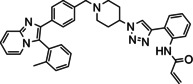	>20000	>20000	>20000
**7**	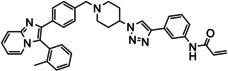	>20000	>20000	>20000
**8**	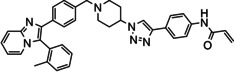	>20000	>20000	>20000
**9**	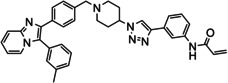	>20000	>20000	>20000
**10**	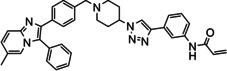	4630±244	>20000	>20000
**11**	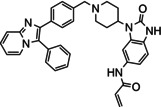	34±20	2563±578	>20000
**12**	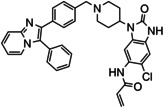	29±5	5587±1518	>20000
**13**	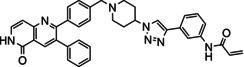	7.0±1.8	ND^[b]^	ND^[b]^
**2**	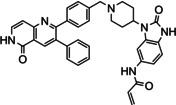	0.8±0.3	56±16	618±24

[a] Borussertib (**2**) was used as control. Data are presented as mean values ± SD (*n*=3, two technical replicates per experiment). [b] ND: not determined.

In this assay, most of the compounds only displayed activity against Akt1 and, as anticipated, little to no measurable activity against any of the other included forms of Akt (Table 1). Only compounds **11**–**13** showed some activity against those other forms of Akt and, as could be seen with these three compounds (and the control **2**), their potency was significantly decreased compared to that against Akt1. Since the remaining compounds were not significantly active against Akt1, any potential inhibitory effect on the other Akt isoforms was likely too low to be observed by this assay.

Of the three regioisomers **3**–**5**, the *meta*‐acrylamide regioisomer **3** displayed the best potency, followed by *ortho*‐acrylamide **4**, and then *para*‐acrylamide **5**, supporting the impression that the orientation of the warhead towards the targeted cysteines plays an important role in covalent bond formation (see similar observations in^[12]^). Compound **3** was the only irreversible Akt inhibitor of this regioisomer set that resulted in quantitative protein labelling under the experimental conditions described in the SI and was, as expected, the most potent of the three. This was followed by compound **4** which showed partial labelling of Akt1 (LC‐MS analysis) and therefore has the acrylamide in a more favourable position for covalent bond formation than compound **5**. This trend could not be further investigated in regioisomers **6**–**8**, as this set of compounds displayed no measurable activity against any of the forms of Akt included in the assay. *Meta*‐methyl compound **9** was likewise inactive, with compound **10** the only of the methylated compounds displaying any activity, and solely against Akt1.

As anticipated, the covalent modifiers of Akt1, compounds **11**–**13**, and to a lesser extent compound **3**, were the most potent against Akt1. Since compounds **11**, **12** and **13** were identified as covalent modifiers of Akt1 and also have inhibitory activity against it, they can be called covalent allosteric inhibitors of Akt1. Compounds **11**–**13** also showed activity against the other forms of Akt. As expected, they were less active against these isoforms which have potentially altered allosteric binding pockets to Akt1. Of all the compounds tested, compound **13** was the most potent, with it having a less than 10‐fold decrease in activity against Akt1 compared to control compound **2**, indicating a generally superior interaction between the protein and the 1,6‐naphthyridinone scaffold as compared to the imidazo[1,2‐*a*]pyridine group.

While IC_50_ values are frequently used to quantify the potential of enzyme inhibition of reversible inhibitors, this parameter is time‐dependant for irreversible inhibitors, making it unsuitable for use in ranking irreversible inhibitors in terms of their inhibitory potential.^[28]^ The kinetic parameters of inactivation, namely the inhibition constant (*K*_I_) and the rate constant of enzyme inactivation (*k*_inact_), are therefore preferentially used to describe irreversible inhibitors.^[28]^ *K*_I_ is defined as the concentration of inhibitor required for half of the maximum potential rate of covalent bond formation, while *k*_inact_ is defined as the maximum potential rate of covalent bond formation.^[29]^ The ratio of *k*_inact_ over *K*_I_, *k*_inact_/*K*_I_, accounts for both the potency of the inhibitor (*K*_I_) and the maximum potential rate of covalent bond formation (*k*_inact_) and thus represents the second‐order rate constant describing the efficiency of covalent complex formation.^[29]^ Using a protocol published by Krippendorff *et al*., *K*_I_ and *k*_inact_ can be directly estimated from time‐dependent IC_50_ determinations by kinetic analysis with HTRF® KinEASE™.^[28]^ This assay was therefore used to determine the kinetic parameters of inactivation against Akt1 for the four irreversible binders, namely compounds **3**, **11**, **12** and **13** (Table 2). The other forms of Akt were not included as all compounds displayed limited potency against these. Borussertib (**2**) was again used as control compound.^[12]^

**Table 2:** The kinetic parameters of inactivation for compounds **3**, **11**, **12** and **13**, measured against Akt1. For ease of comparison, their IC_50_ values are also included.^[a]^

Compd	IC_50_ [nM]	*K*_I_ [nM]	*k*_inact_ [min^−1^]	*k*_inact_/*K*_I_ [μM^−1^s^−1^]
**3**	372.4±48.5	497.2±106.8	0.071±0.006	0.002±0.001
**11**	34.1±20.4	27.2±2.6	0.069±0.004	0.043±0.006
**12**	28.6±5.3	24.7±1.8	0.081±0.008	0.055±0.009
**13**	7.0±1.8	10.5±0.8	0.042±0.012	0.066±0.015
**2**	0.8±0.3	2.2±0.3	0.111±0.020	0.853±0.038

[a] Borussertib (**2**) was included as control. Data are presented as mean values ± SD (*n*=3, two technical replicates per experiment).

Of the compounds tested, compound **13**, shown to have the best IC_50_ against Akt1, had the best inhibition constant (*K*_I_), but interestingly, the less potent compound **12** had a higher rate of enzyme inactivation (*k*_inact_), nearing that of the control compound, borussertib (**2**). This was unfortunately offset by its poor *K*_I_, with compound **13** having better balance between its inhibition constant and rate of enzyme inactivation and displaying the best overall efficiency of covalent bond formation, *k*_inact_/*K*_I_.

When looking at compounds **2** and **3** and their hybrids **11** and **13** (Table 2), compound **13** displayed the best overall properties against Akt1, excluding the control **2**. Compound **3** compared poorly to compounds **2** and **13**, pointing to the shared western portion of inhibitors **2** and **13** being crucial for potent Akt inhibition. Likewise, compound **11** also performed better than compound **3**, pointing to the shared eastern portion of compounds **2** and **11** as the preferred eastern portion for Akt inhibition.

#### Cellular evaluation

The potential *in vitro* antiproliferative activity of the synthesised target compounds was investigated against a number of cancer cell lines by the CellTiter‐Glo® Luminescent Cell Viability Assay (from Promega Corporation, USA, catalogue number: G7570). The half‐maximal effective concentrations (EC_50_) of these compounds were determined against the AN3‐CA, BT‐474, KU‐19‐19, MCF‐7, T‐47D and ZR‐75‐1 cell lines originating from various tissues, i. e., breast, endometrium and bladder (Table S1). These cell lines possess genetic alterations in the PI3K/Akt/mTOR pathway, including PI3K and PTEN, but only one has an Akt mutation, namely the KU‐19‐19 cell line.^[9]^ This cell line possesses a mutated form of Akt1, Akt1^E17K/E49K^, which has glutamic acid to lysine substitutions at amino acid positions 17 and 49.^[9]^

From the results (Table 3) it could be seen that none of the cell lines displayed the same sensitivity to the evaluated compounds than they did to borussertib (**2**), with most of the compounds displaying micromolar antiproliferative activities, if at all, in line with the inferior biochemical activities summarised in Table 1.

**Table 3:** Cellular data for the target compounds synthesised during this project. EC_50_ values are given in nM.^[a]^

Compd	AN3CA	KU‐19‐19	BT‐474	MCF‐7	T47D	ZR‐75‐1
**4**	12271±3485	29370±9587	15375±6134	8554±1247	15150±3254	1751±428
**3**	4754±1537	12371±2884	8274±862	4295±1127	4074±1608	2169±517
**5**	5441±1557	9834±1597	6987±2372	3386±348	3793±1937	3535±440
**6**	14715±2353	21975±4981	>30000	18245±2564	>30000	15509±1861
**7**	5706±706	7691±664	9908±4136	4382±1595	4860±627	4188±1115
**8**	5868±403	7631±636	7388±2337	4303±1180	4521±1587	5318±426
**9**	5134±1277	9448±1040	11167±4733	3610±804	4588±1309	4203±817
**10**	5611±2848	17307±7969	22804±3005	6732±3478	9181±4724	3319±3112
**11**	1028±210	12817±1615	971±301	1105±689	364±87	81±11
**12**	3572±1221	23468±6702	3763±1377	4229±2403	1500±903	227±57
**13**	425±58	5651±1487	469±162	329±225	88±31	20±7
Capivasertib	869±278	3060±2331	1605±450	2653±555	538±30	191±68
Ipatasertib	925±457	4231±3388	2371±745	5036±1830	447±2	219±83
MK‐2206	972±322	5525±927	1682±316	571±111	411±23	63±21
Borussertib (**2**)	191±90	7818±3271	373±54	277±90	48±15	5±1

[a] Data are presented as mean values ± SD (*n*=3, two technical replicates per experiment).

The most sensitive cell line was the breast cancer cell line ZR‐75‐1, while the bladder cancer cell line KU‐19‐19 was the least sensitive of the cell lines. Cell line KU‐19‐19 expresses mutated Akt1 (E17K/E49K) as well as NRAS^Q61R^ and PIK3CA^R1023Q^, potentially mediating significant tolerance against targeted Akt inhibitors.

Surprisingly, of the three regioisomers **3**–**5**, *para*‐acrylamide regioisomer **5**, was the most active against four of the six cell lines, even though it previously displayed the worst potency of the three regioisomers against Akt1 and had no indication of covalent binding to Akt. This activity is potentially from off‐target effects or results from the generally low compound solubility.

Of the methylated compounds **6**–**10**, only compound **10** displayed activity against Akt in the biochemical assays; however, in the cellular assays all five compounds displayed antiproliferative activity, in some cases equalling or exceeding that of the biochemically more potent compounds **3**–**5**. It should be noted that the biochemical data for these compounds could be inaccurate due to a combination of both low potency and low solubility which may have resulted in dose‐response curves that did not go down to full inhibition, potentially skewing the results. Poor solubility was therefore still a problem with these compounds, despite the presence of the added methyl group.

As in the biochemical assays, the best performers, with submicromolar antiproliferative activity for a number of the cell lines, were compounds **11**, **12** and **13**. As with its greater potency against the different forms of Akt, compound **13** was the most active against all of the cell lines, with its antiproliferative activity exceeding that of ATP competitive inhibitors capivasertib and ipatasertib, and reversible allosteric inhibitor MK‐2206 (Figure S3), for some of the cell lines.

## Conclusion

A small library of ten potential Akt inhibitors was synthesised, with the aim to identify novel covalent allosteric Akt inhibitors. The modular click approach allowed for fairly quick access to these novel structures. The biochemical and cellular evaluation of these potential Akt inhibitors led to the identification of three novel covalent allosteric inhibitors of Akt1, namely compounds **11**–**13**. These three compounds further displayed improved potency compared to the previously synthesised related covalent allosteric inhibitor, compound **3**. Unfortunately, while compounds **11**–**13** were irreversible Akt inhibitors and displayed submicromolar antiproliferative activity against a number of cancer cell lines, none matched the highly active inhibitor of the same class, borussertib (**2**), with the most active compounds being related to borussertib **2**, albeit less potent. Notably, Rauh and co‐workers are currently investigating their potent Akt inhibitor borussertib (**2**) in preclinical trials.^[9]^

A number of target compounds were therefore successfully synthesised and evaluated, building on the SAR knowledge of these inhibitors. It is hoped that the additional information on this potentially important class of inhibitors will be useful for the design of future generations of irreversible Akt inhibitors with improved properties.

## Experimental Section

**General procedures**. The chemicals used were purchased from Sigma‐Aldrich or Merck. Reactions were performed in anhydrous conditions with a positive pressure of nitrogen, unless water was used as solvent. TLC was performed using Macherey‐Nagel ALUGRAM® Xtra SIL G UV254 aluminum sheets. Unless otherwise stated, column chromatography was performed under gravity using Sigma silica gel 60 (0.063–0.200 mm mesh particle size, 60 Å). In some cases, column chromatography was performed with a CombiFlash Rf200 UV/Vis system using Sigma silica gel 60 (0.220–0.440 mm mesh particle size, 60 Å).

NMR spectra (^1^H, ^13^C, COSY, NOESY, TOCSY, HSQC and HMBC) were recorded on either one of three instruments: a 300 MHz Varian V NMRS (75 MHz for ^13^C), a 400 MHz Varian Unity Inova (101 MHz for ^13^C) or a 600 MHz Varian Unity Inova (151 MHz for ^13^C). All spectra were obtained at 25 °C. Chemical shifts (δ) are reported in ppm and J‐values are given in Hz. For samples with CDCl_3_ as solvent, referencing was done with the residual solvent peak, unless it was masked by compound peaks and TMS was present, then TMS was used. For DMSO‐d_6_ and MeOD‐d_4_ samples, the residual solvent peaks were used as reference. In certain cases, CDCl_3_ was used as the main solvent and MeOD‐d_4_ as an additive to aid in dissolving the sample (0.6 mL CDCl_3_ with ∼0.05–0.20 mL MeOD‐d_4_ added). This will be indicated in the characterisation.

Infrared spectra were recorded on Thermo Nicolet Nexus 470 FT‐IR, Thermo Nicolet Avatar 330 FT‐IR and Thermo Nicolet iS10 FT‐IR spectrometers, using the diamond Attenuated Total Reflectance (ATR) mode for all three of these. Melting points were obtained using Gallenkamp and Lasany Melting Point Apparatuses.

HRMS was performed on a Waters SYNAPT G2 mass spectrometer. **General procedure for determining compound purity**: ∼1 mg sample was dissolved in MeOH and transferred into a glass vial ready for analysis. A Waters Acquity UHPLC and photo‐diode array detector (PDA) linked to a Waters Synapt G2 quadrupole time‐of‐flight mass spectrometer (Waters Corporation, Milford, MA, USA), was used for LC‐MS analyses. Separation was achieved on a Waters BEH C_18_ UPLC column (2.1×100 mm, 1.7 μm) at 35 °C. Solvent A consisted of 0.1 % formic acid in H_2_O; solvent B consisted of 0.1 % formic acid in MeCN. Gradient elution at a flow rate 0.4 m min^−1^ was used for separation of the compounds, starting with 100 % A and changing to 100 % B over 10 min, washing it 100 % B for 2 min, followed by re‐equilibration to initial conditions over 2 min. Electrospray ionization was applied in the positive mode, using a capillary voltage of 3.5 kV, a cone voltage of 15 V, desolvation temperature of 250 °C and desolvation gas (N_2_) flow of 650 L h^−1^. The rest of the MS settings were optimized for best sensitivity. Data were acquired in MSE mode, consisting of a scan using a low collision energy and a scan using a collision energy ramp from 25 to 60 V, which has the added advantage of acquiring low energy molecular ion data as well as fragmentation data for all compounds. The purity of the peak of interest was determined using the PDA trace at 280 nm and was calculated as the percentage of the peak area of the compound of interest over the total area of any other detected compounds which were not present in the solvent blank.

**Synthesis note**. Compounds **17**, **19**, **24**, **28**, **32**, **36**, **40**, **42** and **52** were synthesised according to literature procedures and their procedures are only given here as reference to those procedures that are based on their synthesis (e. g. **18**, **20** etc.).[^6a^, ^13^, ^14^] Their characterisation corresponded to that reported in Weisner *et al*. and was therefore omitted.^[6a]^

**4‐(Imidazo[1,2‐*a*]pyridin‐2‐yl)benzonitrile (17)**. Following a literature procedure,^[13]^ 2‐bromo‐4’‐cyanoacetophenone **16** (0.501 g, 2.24 mmol), 2‐aminopyridine **14** (1.0 equiv, 0.214 g, 2.27 mmol), EtOH (4.5 mL) and NaHCO_3_ (1.1 equiv, 0.207 g, 2.46 mmol) were heated at 75 °C for 17 h. It was then allowed to cool to room temperature, diluted with H_2_O (125 mL) and extracted with DCM (3×100 mL). The combined organic layers were dried over MgSO_4_ and filtered. The solvent was removed *in vacuo* to afford **17** (0.487 g, 2.22 mmol, 98 %) (R_f_=0.32, 40 % EtOAc/hexane) as a yellow solid with no further purification required. Its characterisation corresponded to the literature.[^6a^, ^30^]

Compound **18** was prepared by a method similar to that for compound **17**.

**4‐(6‐Methylimidazo[1,2‐*a*]pyridin‐2‐yl)benzonitrile (18)**. Dark yellow solid (0.895 g, 3.84 mmol, 85 %) (R_f_=0.30, 40 % EtOAc/hexane). **Decompn**. 224–226 °C. **IR (ATR, cm^−1^)**: 3127 (w), 3037 (w), 2221 (C≡N str, s), 2153 (w), 1608 (s), 1552 (w), 1530 (w), 1479 (w), 1416 (m), 1385 (w), 1344 (w), 1261 (w), 1215 (w). ^**1**^ **H NMR (300 MHz, CDCl_3_) δ** 8.03–7.98 (m, 2H), 7.89 (app. dq, dddd, *J*=1.8, 1.1, 1.1, 1.1 Hz, 1H), 7.82 (d, *J*=0.7 Hz, 1H), 7.70–7.64 (m, 2H), 7.51 (d, *J*=9.3 Hz, 1H), 7.05 (dd, *J*=9.3, 1.8 Hz, 1H), 2.32 (d, *J*=1.1 Hz, 3H, C_Ar_−C*H*_3_). ^**13**^ **C NMR (75 MHz, CDCl_3_) δ** 145.2, 143.5, 138.6, 132.6, 128.8, 126.3, 123.5, 122.9, 119.2, 117.2, 110.9, 109.3, 18.2 (C_Ar_−*C*H_3_). **HRMS (TOF MS ESI+)**: *m/z* calcd for C_15_H_12_N_3_^+^ [M+H]^+^, 234.1031, found 234.1035, error 1.7 ppm.

**4‐(3‐Bromoimidazo[1,2‐*a*]pyridin‐2‐yl)benzonitrile (19)**. Following a literature procedure,^[14]^ 4‐(imidazo[1,2‐*a*]pyridin‐2‐yl)‐benzonitrile **17** (0.521 g, 2.37 mmol) and DCM (8 mL) were cooled to 0 °C by means of an ice bath. Br_2_ (1.1 equiv, 0.13 mL, 0.41 g, 2.5 mmol) was dissolved in DCM (8 mL) and also cooled to 0 °C. This was then slowly added to the reaction flask over 1 h while keeping both at 0 °C. A yellow precipitate formed during the addition of Br_2_ and the reaction was complete after an additional 30 min at 0 °C. The yellow precipitate was filtered through a sinter funnel and washed with cold hexane. The precipitate was suspended in DCM (150 mL), cooled to 0 °C and basified with the slow addition of aqueous NaOH until the pH was between 8 and 10 as shown by universal indicator paper. The two layers were separated, and the aqueous layer was washed with DCM (2×50 mL). The combined organic layers were dried over MgSO_4_, filtered and the solvent was removed *in vacuo* to afford **19** (0.588 g, 1.97 mmol, 83 %) (R_f_=0.47, 40 % EtOAc/hexane) as a pale‐yellow solid which did not require further purification. Its characterisation corresponded to the literature.^[6a]^

Compound **20** was prepared by a method similar to that for compound **19**.

**4‐(3‐Bromo‐6‐methylimidazo[1,2‐*a*]pyridin‐2‐yl)benzonitrile (20)**. Peach solid (1.06 g, 3.39 mmol, 98 %) (R_f_=0.49, 40 % EtOAc/hexane). **Decompn**. 190–192 °C. **IR (ATR, cm^−1^)**: 3080 (w), 3027 (w), 2225 (C≡N str, s), 1609 (s), 1532 (w), 1502 (w), 1475 (s), 1410 (s), 1386 (w), 1344 (C‐N str, s), 1327 (m), 1302 (w), 1270 (w), 1249 (w). ^**1**^ **H NMR (300 MHz, CDCl_3_) δ** 8.34–8.21 (m, 2H), 8.00 (app. dt, ddd, *J*=1.8, 1.1, 0.9 Hz, 1H), 7.81–7.68 (m, 2H), 7.68 (dd, *J*=9.1, 0.9 Hz, 1H), 7.24 (dd, *J*=9.1, 1.8 Hz, 1H), 2.44 (d, *J*=1.1 Hz, 3H, C_Ar_−C*H*_3_). ^**13**^ **C NMR (75 MHz, CDCl_3_) δ** 144.2, 139.3, 136.6, 132.5, 130.3, 128.3, 124.5, 122.0, 119.0, 116.9, 111.9, 93.1, 18.6 (C_Ar_−*C*H_3_). **HRMS (TOF MS ESI+)**: *m/z* calcd for C_15_H_11_BrN_3_^+^ [M+H]^+^, 312.0136, found 312.0132, error 1.3 ppm.

**4‐(3‐Phenylimidazo[1,2‐*a*]pyridin‐2‐yl)benzonitrile (24)**. Following a literature procedure,^[6a]^ 4‐(3‐bromoimidazo[1,2‐*a*]pyridin‐2‐yl)benzonitrile **19** (2.26 g, 7.58 mmol), phenylboronic acid **21** (1.2 equiv, 1.10 g, 9.03 mmol), K_2_CO_3_ (2.4 equiv, 2.51 g, 18.1 mmol) and Pd(PPh_3_)_4_ (6 mol%, 0.568 g, 0.491 mmol) were added to a Schlenk tube and then evacuated and backfilled with N_2_. Degassed 1,4‐dioxane (14 mL) and H_2_O (7 mL) were added and the flask was again evacuated and backfilled with N_2_. The reaction mixture was heated at 110 °C for 20 h and thereafter allowed to cool to room temperature. The clear yellow reaction mixture was diluted with H_2_O (250 mL) and extracted with DCM (3×200 mL). The combined organic layers were dried over MgSO_4_ and filtered, and the solvent was removed *in vacuo*. The crude material was purified by column chromatography (20 % EtOAc/hexane) to afford **24** (1.94 g, 6.57 mmol, 87 %) (R_f_=0.41, 40 % EtOAc/hexane) as an off‐white solid. Its characterisation corresponded to the literature.^[6a]^

Compounds **25**–**27** were prepared by a method similar to that for compound **24**.

**4‐[3‐(*o*‐Tolyl)imidazo[1,2‐*a*]pyridin‐2‐yl]benzonitrile (25)**. White solid (0.502 g, 1.62 mmol, 83 %) (R_f_=0.43, 40 % EtOAc/hexane). **Mp** 156–158 °C. **IR (ATR, cm^−1^)**: 2225 (C≡N str, s), 1742 (w), 1633 (w), 1607 (s), 1548 (w), 1505 (m), 1468 (w), 1443 (w), 1412 (w), 1379 (m), 1360 (m), 1338 (C‐N str, s), 1272 (w), 1234 (m), 1216 (w). ^**1**^ **H NMR (600 MHz, CDCl_3_) δ** 7.79–7.74 (m, 2H), 7.69 (app. dt, ddd, *J*=9.1, 1.2, 1.2 Hz, 1H), 7.56 (app. dt, ddd, *J*=6.8, 1.2, 1.2 Hz, 1H), 7.55–7.52 (m, 2H), 7.49 (app. td, ddd, *J*=7.5, 7.5, 1.5 Hz, 1H), 7.46–7.44 (m, 1H), 7.40–7.37 (m, 1H), 7.31 (dd, *J*=7.5, 1.1 Hz, 1H), 7.25 (ddd, *J*=9.1, 6.7, 1.2 Hz, 1H), 6.76 (app. td, ddd, *J*=6.8, 6.7, 1.2 Hz, 1H), 2.01 (s, 3H, C_Ar_−C*H*_3_). ^**13**^ **C NMR (151 MHz, CDCl_3_) δ** 145.1, 140.1, 139.1, 139.1, 132.3, 131.7, 131.4, 130.4, 128.6, 127.5, 127.4, 125.4, 123.7, 122.0, 119.2, 117.9, 112.9, 110.7, 19.4 (C_Ar_−*C*H_3_). **HRMS (TOF MS ESI+)**: *m/z* calcd for C_21_H_16_N_3_^+^ [M+H]^+^, 310.1344, found 310.1342, error 0.6 ppm.

**4‐[3‐(*m*‐Tolyl)imidazo[1,2‐*a*]pyridin‐2‐yl]benzonitrile (26)**. Off‐white solid (0.446 g, 1.44 mmol, 82 %) (R_f_=0.43, 40 % EtOAc/hexane). **Mp** 155–157 °C. **IR (ATR, cm^−1^)**: 3029 (w), 2923 (w), 2224 (C≡N str, s), 1916 (w), 1788 (w), 1631 (w), 1606 (s), 1585 (w), 1543 (w), 1504 (m), 1470 (m), 1444 (w), 1411 (w), 1381 (w), 1356 (m), 1339 (C‐N str, s), 1306 (m), 1269 (w), 1238 (m). ^**1**^ **H NMR (400 MHz, CDCl_3_) δ** 7.90 (app. dt, ddd, *J*=6.9, 1.2, 1.2 Hz, 1H), 7.83–7.75 (m, 2H), 7.66 (app. dt, ddd, *J*=9.1, 1.2, 1.2 Hz, 1H), 7.60–7.50 (m, 2H, 7.45 (app. t, dd, *J*=7.6, 7.6 Hz, 1H), 7.38–7.30 (m, 1H), 7.26–7.20 (m, 3H), 6.76 (app. td, ddd, *J*=6.9, 6.8, 1.2 Hz, 1H), 2.42 (s, 3H, C_Ar_−C*H*_3_). ^**13**^ **C NMR (101 MHz, CDCl_3_) δ** 145.1, 140.1, 139.9, 139.1, 132.2, 131.1, 130.4, 129.9, 129.2, 128.3, 127.8, 125.5, 123.7, 122.9, 119.3, 117.8, 112.8, 110.7, 21.6 (C_Ar_−*C*H_3_). **HRMS (TOF MS ESI+)**: *m/z* calcd for C_21_H_16_N_3_^+^ [M+H]^+^, 310.1344, found 310.1351, error 2.3 ppm.

**4‐(6‐Methyl‐3‐phenylimidazo[1,2‐*a*]pyridin‐2‐yl)benzonitrile (27)**. Pale yellow solid (0.528 g, 1.71 mmol, 76 %) (R_f_=0.44, 40 % EtOAc/hexane). **Mp** 196–198 °C. **IR (ATR, cm^−1^)**: 3054 (m), 2973 (w), 2923 (w), 2223 (C≡N str, s), 1907 (w), 1641 (w), 1609 (s), 1551 (m), 1537 (m), 1512 (m), 1456 (w), 1443 (w), 1411 (m), 1386 (m), 1335 (C‐N str, s), 1273 (m), 1255 (w), 1226 (m). ^**1**^ **H NMR (600 MHz, CDCl_3_) δ** 7.77–7.73 (m, 2H), 7.67 (app. dt, ddd, *J*=1.5, 1.2, 1.2 Hz, 1H), 7.59–7.51 (m, 6H), 7.44–7.41 (m, 2H), 7.09 (dd, *J*=9.2, 1.5 Hz, 1H), 2.27 (d, *J*=1.2 Hz, 3H, C_Ar_−C*H*_3_). ^**13**^ **C NMR (151 MHz, CDCl_3_) δ** 144.3, 140.2, 139.2, 132.2, 130.8, 130.0, 129.6, 128.8, 128.2, 122.7, 122.4, 121.1, 119.3, 117.2, 110.6, 18.5 (C_Ar_−*C*H_3_). Two carbon peaks are suspected to overlap, presenting as one. **HRMS (TOF MS ESI+)**: *m/z* calcd for C_21_H_16_N_3_^+^ [M+H]^+^, 310.1344, found 310.1344, error 0 ppm.

**4‐(3‐Phenylimidazo[1,2‐*a*]pyridin‐2‐yl)benzaldehyde (28)**. Following a literature procedure,^[6a]^ 4‐(3‐phenylimidazo[1,2‐*a*]pyridin‐2‐yl)benzonitrile **24** (0.420 g, 1.42 mmol), formic acid (7 mL) and Raney nickel were degassed for 5 min and then heated at 90 °C for 2.5 h, after which it was allowed to cool to room temperature. Saturated aqueous NaHCO_3_ (200 mL) was added to the reaction mixture until the pH was over 7 as shown by universal indicator paper. The mixture was filtered through celite and extraction was done with EtOAc (4×100 mL). The combined organic layers were dried over MgSO_4_, filtered and the solvent was removed *in vacuo*. The yellow crude material was purified by column chromatography (40 % EtOAc/hexane) to afford **28** (0.270 g, 0.905 mmol, 64 %) (R_f_=0.32, 40 % EtOAc/hexane) as a yellow solid. Its characterisation corresponded to the literature.^[6a]^

Compounds **29**–**31** were prepared by a method similar to that for compound **28**.

**4‐[3‐(*o*‐Tolyl)imidazo[1,2‐*a*]pyridin‐2‐yl]benzaldehyde (29)**. Pale yellow solid (0.546 g, 1.75 mmol, 88 %) (R_f_=0.39, 40 % EtOAc/hexane). **Mp** 138–140 °C. **IR (ATR, cm^−1^)**: 3103 (w), 2812 (w), 2728 (w), 1725 (w), 1693 (C=O str, s), 1633 (w), 1604 (s), 1569 (m), 1504 (m), 1469 (w), 1444 (w), 1417 (w), 1378 (w), 1361 (w), 1340 (C‐N str, s), 1304 (w), 1271 (w), 1234 (m), 1210 (s). ^**1**^ **H NMR (300 MHz, CDCl_3_) δ** 9.95 (s, 1H, −C*H*O), 7.86–7.81 (m, 2H), 7.79–7.75 (m, 2H), 7.70 (ddd, *J*=9.1, 1.2, 1.2 Hz, 1H), 7.57 (ddd, *J*=6.9, 1.3, 1.2 Hz, 1H), 7.52–7.42 (m, 2H), 7.42–7.30 (m, 2H), 7.24 (ddd, *J*=9.1, 6.7, 1.3 Hz, 1H), 6.75 (ddd, *J*=6.9, 6.7, 1.2 Hz, 1H), 2.01 (s, 3H, C_Ar_−C*H*_3_). ^**13**^ **C NMR (75 MHz, CDCl_3_) δ** 192.0 (−*C*HO), 145.1, 140.6, 139.1, 135.2, 131.8, 131.3, 130.2, 130.1, 130.0, 128.8, 127.5, 127.5, 127.3, 125.3, 123.7, 117.8, 112.8, 19.5 (C_Ar_−*C*H_3_). **HRMS (TOF MS ESI+)**: *m/z* calcd for C_21_H_17_N_2_O^+^ [M+H]^+^, 313.1341, found 313.1335, error 1.9 ppm.

**4‐[3‐(*m*‐Tolyl)imidazo[1,2‐*a*]pyridin‐2‐yl]benzaldehyde (30)**. Yellow solid (0.395 g, 1.26 mmol, 76 %) (R_f_=0.23, 40 % EtOAc/hexane). **Mp** 134–136 °C. **IR (ATR, cm^−1^)**: 3049 (w), 2811 (w), 2725 (m), 1685 (C=O str, s), 1633 (w), 1603 (s), 1571 (m), 1504 (m), 1480 (w), 1442 (w), 1413 (w), 1385 (w), 1357 (m), 1342 (C−N str, s), 1305 (m), 1268 (m), 1244 (m), 1212 (s). ^**1**^ **H NMR (600 MHz, CDCl_3_) δ** 9.96 (s, 1H, −C*H*O), 7.92 (app. dt, ddd, *J*=6.9, 1.2, 1.2 Hz, 1H), 7.88–7.84 (m, 2H), 7.80–7.76 (m, 2H), 7.68 (app. dt, ddd, *J*=9.1, 1.2, 1.2 Hz, 1H), 7.44 (app. t, dd, *J*=7.6, 7.6 Hz, 1H, 7.36–7.31 (m, 1H), 7.27–7.18 (m, 3H), 6.75 (app. td, ddd, *J*=6.9, 6.8, 1.2 Hz, 1H), 2.42 (s, 3H, C_Ar_−C*H*_3_). ^**13**^ **C NMR (151 MHz, CDCl_3_) δ** 192.1 (−*C*HO), 145.1, 140.8, 140.6, 139.8, 135.2, 131.2, 130.3, 129.9, 129.8, 129.4, 128.4, 127.9, 125.3, 123.7, 122.9, 117.8, 112.7, 21.6 (C_Ar_−*C*H_3_). **HRMS (TOF MS ESI+)**: *m/z* calcd for C_21_H_17_N_2_O^+^ [M+H]^+^, 313.1341, found 313.1334, error 2.2 ppm.

**4‐(6‐Methyl‐3‐phenylimidazo[1,2‐*a*]pyridin‐2‐yl)benzaldehyde (31)**. Off‐white solid (0.314 g, 1.01 mmol, 73 %) (R_f_=0.32, 40 % EtOAc/hexane). **Mp** 168–170 °C. **IR (ATR, cm^−1^)**: 3061 (w), 2848 (w), 1695 (C=O str, s), 1603 (s), 1570 (w), 1531 (w), 1512 (m), 1443 (m), 1411 (w), 1388 (w), 1336 (C−N str, m), 1304 (m), 1253 (w), 1211 (s). ^**1**^ **H NMR (600 MHz, CDCl_3_) δ** 9.96 (s, 1H, −C*H*O), 7.85–7.80 (m, 2H), 7.79–7.75 (m, 2H), 7.69 (app. dt, ddd, *J*=1.9, 1.1, 1.1 Hz, 1H), 7.59 (dd, *J*=9.2, 1.1 Hz, 1H), 7.57–7.49 (m, 3H), 7.47–7.42 (m, 2H), 7.09 (dd, *J*=9.2, 1.9 Hz, 1H), 2.27 (d, *J*=1.1 Hz, 3H, C_Ar_−C*H*_3_). ^**13**^ **C NMR (151 MHz, CDCl_3_) δ** 192.1 (−*C*HO), 144.3, 140.8, 140.8, 135.1, 130.9, 129.9, 129.9, 129.8, 129.4, 128.6, 128.3, 122.6, 122.5, 121.1, 117.2, 18.5 (C_Ar_−*C*H_3_). **HRMS (TOF MS ESI+)**: *m/z* calcd for C_21_H_17_N_2_O^+^ [M+H]^+^, 313.1341, found 313.1330, error 3.5 ppm.

**[4‐(3‐Phenylimidazo[1,2‐*a*]pyridin‐2‐yl)phenyl]methanol (32)**. Following a literature procedure,^[6a]^ 4‐(3‐phenylimidazo[1,2‐*a*]pyridin‐2‐yl)‐benzaldehyde **28** (0.172 g, 0.577 mmol) and MeOH (3 mL) were cooled to 0 °C and NaBH_4_ (2.4 equiv, 0.052 g, 1.4 mmol) was added. The resulting clear yellow reaction mixture was left to stir at 0 °C for 30 min after which it was allowed to return to room temperature and was stirred for a further 25 min. Upon complete consumption of starting material, the solvent was removed *in vacuo*. H_2_O (25 mL) was added to the crude material and extraction was done using EtOAc (5×50 mL). The combined organic layers were dried over MgSO_4_ and filtered. The solvent was removed *in vacuo* to yield the crude product which was purified by column chromatography (60 % EtOAc/hexane) to afford **32** (0.140 g, 0.465 mmol, 81 %) (R_f_=0.11, 50 % EtOAc/hexane) as a white solid. Its characterisation corresponded to the literature.^[6a]^

Compounds **33**–**35** were prepared by a method similar to that for compound **32**.

**{4‐[3‐(*o*‐Tolyl)imidazo[1,2‐*a*]pyridin‐2‐yl]phenyl}methanol (33)**. White solid (0.458 g, 1.46 mmol, 90 %) (R_f_=0.13, 40 % EtOAc/hexane). **Mp** 58–60 °C. **IR (ATR, cm^−1^)**: 3362 (w), 3209 (O‐H str, s), 2915 (w), 2856 (s), 2547 (w), 1733 (m), 1633 (m), 1502 (m), 1473 (w), 1444 (w), 1414 (m), 1382 (s), 1357 (m), 1341 (C‐N str, s), 1274 (m), 1237 (C‐O str, s), 1208 (m). ^**1**^ **H NMR (300 MHz, CDCl_3_) δ** 7.68 (app. dt, ddd, *J*=9.1, 1.2, 1.2 Hz, 1H), 7.62–7.57 (m, 2H), 7.56 (app. dt, ddd, *J*=6.8, 1.3, 1.2 Hz, 1H), 7.48–7.38 (m, 2H), 7.38–7.29 (m, 2H), 7.25–7.21 (m, 2H), 7.20 (ddd, *J*=9.1, 6.8, 1.3 Hz, 1H), 6.72 (app. td, ddd, *J*=6.8, 6.8, 1.2 Hz, 1H), 4.64 (s, 2H, C*H*_2_−OH), 2.71 (s, 1H, OH), 2.00 (s, 3H, C_Ar_−C*H*_3_). ^**13**^ **C NMR (75 MHz, CDCl_3_) δ** 144.9, 142.0, 140.5, 139.3, 133.6, 131.9, 131.1, 129.8, 129.3, 127.4, 127.2, 127.1, 124.8, 123.5, 120.4, 117.5, 112.4, 65.1 (*C*H_2_−OH), 19.5 (C_Ar_−*C*H_3_). **HRMS (TOF MS ESI+)**: *m/z* calcd for C_21_H_19_N_2_O^+^ [M+H]^+^, 315.1497, found 315.1499, error 0.6 ppm.

**{4‐[3‐(*m*‐Tolyl)imidazo[1,2‐*a*]pyridin‐2‐yl]phenyl}methanol (34)**. Off‐white solid (0.309 g, 0.982 mmol, 91 %) (R_f_=0.08, 40 % EtOAc/hexane). **Mp** 166–168 °C. **IR (ATR, cm^−1^)**: 3190 (O−H str, s), 2909 (w), 2843 (m), 1738 (w), 1633 (m), 1605 (m), 1501 (m), 1476 (m), 1444 (w), 1414 (m), 1385 (m), 1354 (s), 1340 (C‐N str, s), 1271 (m), 1230 (m), 1203 (m). ^**1**^ **H NMR (600 MHz, CDCl_3_) δ** 7.93 (app. dt, ddd, *J*=6.8, 1.3, 1.2 Hz, 1H), 7.66 (app. dt, ddd, *J*=9.0, 1.2, 1.2 Hz, 1H), 7.62–7.58 (m, 2H), 7.39 (app. t, dd, *J*=7.6, 7.6 Hz, 1H), 7.28 (d, *J*=7.6 Hz, 1H), 7.25–7.20 (m, 4H), 7.18 (ddd, *J*=9.0, 6.7, 1.3 Hz, 1H), 6.72 (app. td, ddd, *J*=6.8, 6.7, 1.2 Hz, 1H), 4.65 (s, 2H, C*H*_2_−OH), 3.07 (br s, 1H, OH), 2.39 (s, 3H, C_Ar_−C*H*_3_). ^**13**^ **C NMR (151 MHz, CDCl_3_) δ** 144.8, 142.1, 140.6, 139.4, 133.3, 131.2, 129.8, 129.8, 129.6, 128.3, 128.0, 127.0, 124.8, 123.5, 121.4, 117.5, 112.4, 65.0 (*C*H_2_−OH), 21.6 (C_Ar_−*C*H_3_). **HRMS (TOF MS ESI+)**: *m/z* calcd for C_21_H_19_N_2_O^+^ [M+H]^+^, 315.1497, found 315.1501, error 1.3 ppm.

**[4‐(6‐Methyl‐3‐phenylimidazo[1,2‐*a*]pyridin‐2‐yl)phenyl]methanol (35)**. White solid (0.197 g, 0.626 mmol, 76 %) (R_f_=0.20, 60 % EtOAc/hexane). **Mp** 196–198 °C. **IR (ATR, cm^−1^)**: 3180 (O‐H str, s), 2914 (m), 2857 (m), 1537 (m), 1513 (m), 1478 (w), 1441 (m), 1410 (s), 1390 (s), 1363 (m), 1336 (C‐N str, s), 1310 (w), 1276 (m), 1231 (m), 1206 (m). ^**1**^ **H NMR (600 MHz, CDCl_3_) δ** 7.71 (app. dt, ddd, *J*=1.5, 1.2, 1.2 Hz, 1H), 7.59–7.55 (m, 3H), 7.53–7.49 (m, 2H), 7.49–7.45 (m, 1H), 7.45–7.40 (m, 2H), 7.24–7.20 (m, 2H), 7.05 (dd, *J*=9.1, 1.5 Hz, 1H), 4.65 (s, 2H, C*H*_2_−OH), 3.00 (br s, 1H, OH), 2.25 (d, *J*=1.2 Hz, 3H, C_Ar_−C*H*_3_). ^**13**^ **C NMR (151 MHz, CDCl_3_) δ** 144.0, 142.1, 140.5, 133.5, 130.9, 130.9, 130.2, 129.6, 128.9, 128.2, 128.1, 127.0, 122.1, 121.0, 116.9, 65.0 (*C*H_2_−OH), 18.4 (C_Ar_−*C*H_3_). **HRMS (TOF MS ESI+)**: *m/z* calcd for C_21_H_19_N_2_O^+^ [M+H]^+^, 315.1497, found 315.1495, error 0.6 ppm.

**2‐[4‐(Chloromethyl)phenyl]‐3‐phenylimidazo[1,2‐*a*]pyridine (36)**. Following a literature procedure,^[6a]^ [4‐(3‐phenylimidazo[1,2‐*a*]pyridin‐2‐yl)phenyl]methanol **32** (0.048 g, 0.16 mmol) and DMF (3 mL) was heated at 90 °C. PPh_3_ (4.1 equiv, 0.175 g, 0.667 mmol) was dissolved in CCl_4_ (1 mL) and slowly added to the heated reaction mixture. The yellow reaction mixture was heated at 90 °C for 26 h after which it was cooled to room temperature and diluted with brine (100 mL). The product was extracted with EtOAc (3×75 mL) and the combined organic layers were washed with 5 % aqueous LiCl (2×25 mL), dried over MgSO_4_ and filtered. The solvent was removed *in vacuo* to yield the crude product which was purified by column chromatography utilizing the CombiFlash system (5 % to 20 % to 40 % EtOAc/hexane) to afford **36** (0.41 g, 0.13 mmol, 79 %) (R_f_=0.40, 40 % EtOAc/hexane) as a yellow semi‐solid. **IR (ATR, cm^−1^)**: 2008 (w), 1634 (m), 1609 (m), 1511 (s), 1475 (m), 1445 (m), 1413 (w), 1385 (m), 1355 (m), 1345 (C‐N str, s), 1260 (m), 1236 (m), 1212 (m). ^**1**^ **H NMR (300 MHz, CDCl_3_) δ** 7.94 (app. dt, ddd, *J*=6.9, 1.2, 1.2 Hz, 1H), 7.73–7.62 (m, 3H), 7.62–7.40 (m, 5H), 7.33–7.28 (m, 2H), 7.21 (ddd, *J*=9.1, 6.7, 1.2 Hz, 1H), 6.74 (app. td, ddd, *J*=6.9, 6.7, 1.2 Hz, 1H), 4.57 (s, 2H, C*H*_2_−Cl). ^**13**^ **C NMR (101 MHz, CDCl_3_) δ** 144.9, 141.7, 136.6, 134.5, 130.9, 129.8, 129.8, 129.2, 128.7, 128.4, 125.0, 123.5, 121.5, 117.7, 112.5, 46.3 (*C*H_2_−Cl). **HRMS (TOF MS ESI+)**: *m/z* calcd for C_20_H_16_ClN_2_^+^ [M+H]^+^, 319.1002 found 319.0993, error 2.8 ppm.

Compounds **37**–**39** were prepared by a method similar to that for compound **36**.

**2‐[4‐(Chloromethyl)phenyl]‐3‐(*o‐*tolyl)imidazo[1,2‐*a*]pyridine (37)**. Orange semi‐solid (0.388 g, 1.16 mmol, 90 %) (R_f_=0.46, 40 % EtOAc/hexane). **IR (ATR, cm^−1^)**: 3366 (m), 3061 (m), 2954 (m), 2866 (w), 1734 (m), 1677 (w), 1633 (m), 1613 (w), 1510 (s), 1473 (m), 1444 (m), 1415 (m), 1382 (s), 1358 (m), 1342 (C‐N str, s), 1265 (s), 1237 (s), 1214 (m). ^**1**^ **H NMR (300 MHz, CDCl_3_) δ** 7.72–7.63 (m, 3H), 7.55 (app. dt, ddd, *J*=6.8, 1.3, 1.2 Hz, 1H), 7.50–7.40 (m, 2H), 7.40–7.32 (m, 2H), 7.32–7.26 (m, 2H), 7.21 (ddd, *J*=9.1, 6.7, 1.3 Hz, 1H), 6.72 (app. td, ddd, *J*=6.8, 6.7, 1.2 Hz, 1H), 4.55 (s, 2H, C*H*_2_−Cl), 2.01 (s, 3H, C_Ar_−C*H*_3_). ^**13**^ **C NMR (75 MHz, CDCl_3_) δ** 144.9, 141.5, 139.3, 136.5, 134.7, 131.9, 131.2, 130.0, 129.2, 128.8, 127.5, 127.2, 124.8, 123.6, 120.7, 117.6, 112.5, 46.3 (*C*H_2_−Cl), 19.5 (C_Ar_−*C*H_3_). **HRMS (TOF MS ESI+)**: *m/z* calcd for C_21_H_18_ClN_2_^+^ [M+H]^+^, 333.1159, found 333.1156, error 0.9 ppm.

**2‐[4‐(Chloromethyl)phenyl]‐3‐(*m‐*tolyl)imidazo[1,2‐*a*]pyridine (38)**. Dark yellow semi‐solid (0.096 g, 0.29 mmol, 34 %) (R_f_=0.50, 40 % EtOAc/hexane). **IR (ATR, cm^−1^)**: 3401 (w), 2920 (w), 1659 (m), 1606 (m), 1509 (s), 1409 (m), 1346 (C‐N str, s), 1273 (s), 1244 (s). ^**1**^ **H NMR (600 MHz, CDCl_3_) δ** 7.91 (app. dt, ddd, *J*=6.8, 1.2, 1.2 Hz, 1H), 7.70–7.68 (m, 2H), 7.67 (app. dt, ddd, *J*=9.1, 1.2, 1.2 Hz, 1H), 7.42 (app. t, dd, *J*=7.8, 7.6 Hz, 1H), 7.33–7.26 (m, 4H), 7.24 (ddd, *J*=7.8, 2.0, 1.1 Hz, 1H), 7.19 (ddd, *J*=9.1, 6.7, 1.2 Hz, 1H), 6.72 (app. td, ddd, *J*=6.8, 6.7, 1.2 Hz, 1H), 4.57 (s, 2H, C*H*_2_−Cl), 2.41 (s, 3H, C_Ar_−C*H*_3_). ^**13**^ **C NMR (151 MHz, CDCl_3_) δ** 144.9, 141.6, 139.6, 136.5, 134.6, 131.3, 130.0, 129.8, 129.7, 128.7, 128.3, 128.0, 124.8, 123.6, 121.7, 117.6, 112.4, 46.4 (*C*H_2_−Cl), 21.6 (C_Ar_−*C*H_3_). **HRMS (TOF MS ESI+)**: *m/z* calcd for C_21_H_18_ClN_2_^+^ [M+H]^+^, 333.1159, found 333.1155, error 1.2 ppm.

**2‐[4‐(Chloromethyl)phenyl]‐6‐methyl‐3‐phenylimidazo[1,2‐*a*]pyridine (39)**. Pale peach solid (0.152 g, 0.455 mmol, 93 %) (R_f_=0.65, 40 % EtOAc/hexane). **Mp** 145–147 °C. **IR (ATR, cm^−1^)**: 3057 (m), 2921 (w), 1728 (w), 1613 (m), 1537 (s), 1512 (m), 1443 (m), 1411 (m), 1387 (s), 1333 (C‐N str, s), 1272 (s), 1261 (s), 1222 (m). ^**1**^ **H NMR (600 MHz, CDCl_3_) δ** 7.69 (app. dt, ddd, *J*=1.5, 1.2, 1.1 Hz, 1H), 7.66–7.63 (m, 2H), 7.58 (dd, *J*=9.2, 1.1 Hz, 1H), 7.56–7.53 (m, 2H), 7.52–7.48 (m, 1H), 7.46–7.43 (m, 2H), 7.30–7.27 (m, 2H), 7.06 (dd, *J*=9.2, 1.5 Hz, 1H), 4.56 (s, 2H, C*H*_2_−Cl), 2.26 (d, *J*=1.2 Hz, 3H, C_Ar_−C*H*_3_). ^**13**^ **C NMR (151 MHz, CDCl_3_) δ** 144.1, 141.7, 136.4, 134.7, 131.0, 130.2, 129.7, 129.1, 128.7, 128.3, 128.1, 122.1, 121.2, 121.0, 117.1, 46.4 (*C*H_2_−Cl), 18.5 (C_Ar_−*C*H_3_). **HRMS (TOF MS ESI+)**: *m/z* calcd for C_21_H_18_ClN_2_^+^ [M+H]^+^, 333.1159, found 333.1159, error 0 ppm.

***tert*** **‐Butyl 4‐azidopiperidine‐1‐carboxylate (47)**. Following a literature procedure,^[6a]^ *tert‐*butyl 4‐bromopiperidine‐1‐carboxylate **46** (1.51 g, 5.71 mmol) and DMSO (5 mL) was stirred at room temperature until the starting material had dissolved. NaN_3_ (1.2 equiv, 0.461 g, 7.09 mmol) was then added and the colourless reaction mixture was stirred at 60 °C for 18 h. The reaction mixture was diluted with brine (75 mL) and the product was extracted with EtOAc (8×100 mL). The combined organic layers were dried over MgSO_4_, filtered and concentrated *in vacuo*. The resulting crude product was purified by column chromatography (hexane to 5 % to 20 % EtOAc/hexane) to afford **47** (1.23 g, 5.46 mmol, 96 %) (R_f_=0.47, 10 % EtOAc/hexane) as a colourless oil. Its characterisation corresponded to the literature.^[6a]^

**4‐Azidopiperidin‐1‐ium chloride (41)**. Following a literature procedure,^[6a]^ *tert*‐butyl 4‐azidopiperidine‐1‐carboxylate **47** (0.819 g, 3.62 mmol) was cooled to 0 °C by means of an ice bath. 1,4‐Dioxane (2 mL) and 4.0 M HCl in 1,4‐dioxane (2 mL) were added to the flask. The reaction mixture was stirred at room temperature for 24 h and additional 1,4‐dioxane (4 mL) was added after the first 20 h. Following starting material consumption, the solvent was removed *in vacuo* to afford **41** (0.580 g, 3.57 mmol, 99 %) as a white solid which required no further purification. Its characterisation corresponded to the literature.^[6a]^

Compound **40** was prepared by a method similar to that for compound **41**, and neutralised thereafter as described below.

**4‐Azidopiperidine (40)**. After completion of synthesis, the reaction mixture was diluted with MeOH (13 mL) and K_2_CO_3_ (2.8 equiv, 2.7 g, 20 mmol) and stirred at RT for 30 min. The mixture was filtered through Celite, washed with DCM (100 mL) and concentrated *in vacuo*. The crude material was purified by column chromatography (DCM to 10 % MeOH/DCM) to afford the title compound **40** (0.447 g, 3.54 mmol, 50 %) as a pale yellow solid. The neutral piperidine was very polar, not easily seen on TLC as it was UV inactive, and thus required ninhydrin reagent for visualization. It is probable that a significant amount of material was “lost” during purification, either due to its polarity, or from an inability to find the product in the collected fractions (or both). Its characterisation corresponded to the literature.^[20]^ ^**1**^ **H NMR (300 MHz, CDCl_3_) δ** 6.30 (s, 1H), 3.84 (app. tt, dddd, *J*=6.7, 6.7, 3.4, 3.4 Hz, 1H), 3.27 (ddd, *J*=12.8, 9.1, 3.6 Hz, 2H), 3.15–3.03 (m, 2H), 2.22–2.08 (m, 2H), 1.99–1.82 (m, 2H). ^**13**^ **C NMR (151 MHz, CDCl_3_) δ** 54.3, 40.6, 27.4.

**2‐{4‐[(4‐Azidopiperidin‐1‐yl)methyl]phenyl}‐3‐phenylimidazo[1,2‐*a*]pyridine (42)**. Following a literature procedure,^[6a]^ 2‐[4‐(chloromethyl)phenyl]‐3‐phenylimidazo[1,2‐*a*]pyridine **36** (0.247 g, 0.775 mmol), DMF (5 mL), 4‐azidopiperidine **40** (1.6 equiv, 0.155 g, 1.23 mmol) and Et_3_N (2.0 equiv, 0.22 mL, 0.16 g, 1.6 mmol) were heated at 60 °C for 23 h after which it was allowed to cool to room temperature and diluted with H_2_O (100 mL). The product was extracted with EtOAc (3×150 mL) and the combined organic layers were dried over MgSO_4_ and filtered. The solvent was removed *in vacuo* to afford the crude product which was purified twice by column chromatography utilizing the CombiFlash system (20 % EtOAc/hexane to EtOAc) to afford **42** (0.199 g, 0.487 mmol, 63 %) (R_f_=0.12, 80 % EtOAc/hexane) as a thick yellow oil. Its characterisation corresponded to the literature.^[6a]^

Compounds **43**–**45** were prepared by a method similar to that for compound **42**, but using 4‐azidopiperidin‐1‐ium chloride **41**, not 4‐azidopiperidine **40**.

**2‐{4‐[(4‐Azidopiperidin‐1‐yl)methyl]phenyl}‐3‐(*o‐*tolyl)imidazo[1,2‐*a*]pyridine (43)**. Yellow semi‐solid (0.327 g, 0.775 mmol, 79 %) (R_f_=0.14, 40 % EtOAc/hexane). **IR (ATR, cm^−1^)**: 3061 (w), 2942 (m), 2803 (m), 2763 (m), 2088 (N=N=N str, s), 1736 (w), 1677 (m), 1633 (w), 1510 (m), 1468 (w), 1447 (m), 1415 (w), 1382 (m), 1362 (m), 1340 (C‐N str, s), 1235 (s). ^**1**^ **H NMR (600 MHz, CDCl_3_) δ** 7.68 (app. dt, ddd, *J*=9.0, 1.2, 1.2 Hz, 1H), 7.64–7.59 (m, 2H), 7.55 (app. dt, ddd, *J*=6.8, 1.2, 1.2 Hz, 1H), 7.45 (ddd, *J*=8.4, 6.6, 1.6 Hz, 1H), 7.42 (dd, *J*=7.4, 1.6 Hz, 1H), 7.38–7.32 (m, 2H), 7.22–7.16 (m, 3H), 6.71 (app. td, ddd, *J*=6.8, 6.7, 1.2 Hz, 1H), 3.45 (s, 2H, C_Ar_−C*H*_2_−N), 3.39–3.32 (m, 1H, C*H*‐N_3_), 2.77–2.71 (m, 2H), 2.16–2.10 (m, 2H), 2.01 (s, 3H, C_Ar_−C*H*_3_), 1.90–1.83 (m, 2H), 1.69–1.60 (m, 2H). ^**13**^ **C NMR (151 MHz, CDCl_3_) δ** 144.9, 142.0, 139.3, 137.5, 133.4, 132.0, 131.1, 129.8, 129.5, 129.2, 127.1, 124.6, 123.5, 120.3, 117.5, 112.3, 62.8 (C_Ar_−*C*H_2_−N), 57.9 (*C*H−N_3_), 51.3, 31.0, 19.5 (C_Ar_−*C*H_3_), one carbon signal not observed in spectrum. **HRMS (TOF MS ESI+)**: *m/z* calcd for C_26_H_27_N_6_^+^ [M+H]^+^, 423.2297, found 423.2295, error 0.5 ppm.

**2‐{4‐[(4‐Azidopiperidin‐1‐yl)methyl]phenyl}‐3‐(*m‐*tolyl)imidazo[1,2‐*a*]pyridine (44)**. Yellow semi‐solid (0.078 g, 0.18 mmol, 81 %) (R_f_=0.10, 40 % EtOAc/hexane). **IR (ATR, cm^−1^)**: 2943 (s), 2802 (m), 2764 (m), 2088 (N=N=N str, s), 1736 (s), 1676 (w), 1633 (w), 1606 (w), 1508 (m), 1447 (w), 1468 (w), 1414 (w), 1360 (m), 1339 (C‐N str, s), 1310 (w), 1239 (s). ^**1**^ **H NMR (600 MHz, CDCl_3_) δ** 7.91 (app. dt, ddd, *J*=6.8, 1.2, 1.2 Hz, 1H), 7.68–7.62 (m, 3H), 7.41 (app. t, dd, *J*=7.6, 7.6 Hz, 1H), 7.31–7.28 (m, 1H), 7.27–7.26 (m, 1H), 7.26–7.23 (m, 1H), 7.22–7.20 (m, 2H), 7.18 (ddd, *J*=9.1, 6.7, 1.2 Hz, 1H), 6.71 (app. td, ddd, *J*=6.8, 6.7, 1.2 Hz, 1H), 3.47 (s, 2H, C_Ar_−C*H*_2_−N), 3.38–3.35 (m, 1H, C*H*‐N_3_), 2.77–2.72 (m, 2H), 2.41 (s, 3H, C_Ar_−C*H*_3_), 2.18–2.11 (m, 2H), 1.91–1.83 (m, 2H), 1.70–1.61 (m, 2H). ^**13**^ **C NMR (151 MHz, CDCl_3_) δ** 144.8, 142.2, 139.4, 137.5, 133.3, 131.3, 130.0, 129.8, 129.6, 129.1, 128.0, 127.9, 124.6, 123.5, 121.3, 117.6, 112.2, 62.8 (C_Ar_−*C*H_2_−N), 57.9 (*C*H‐N_3_), 51.3, 31.0, 21.6 (C_Ar_−*C*H_3_). **HRMS (TOF MS ESI+)**: *m/z* calcd for C_26_H_27_N_6_^+^ [M+H]^+^, 423.2297, found 423.2292, error 1.2 ppm.

**2‐{4‐[(4‐Azidopiperidin‐1‐yl)methyl]phenyl}‐6‐methyl‐3‐phenylimidazo[1,2‐*a*]pyridine (45)**. Yellow solid (0.100 g, 0.237 mmol, 78 %) (R_f_=0.37, 5 % MeOH/EtOAc). **Mp** 134–136 °C. **IR (ATR, cm^−1^)**: 2929 (m), 2807 (m), 2760 (m), 2083 (N=N=N str, s), 1536 (w), 1511 (m), 1474 (w), 1443 (m), 1410 (w), 1387 (s), 1333 (C‐N str, s), 1307 (m), 1257 (s). ^**1**^ **H NMR (600 MHz, CDCl_3_) δ** 7.70–7.68 (m, 1H), 7.62–7.59 (m, 2H), 7.57 (dd, *J*=9.1, 1.0 Hz, 1H), 7.55–7.52 (m, 2H), 7.50–7.47 (m, 1H), 7.46–7.44 (m, 2H), 7.21–7.18 (m, 2H), 7.04 (dd, *J*=9.1, 1.7 Hz, 1H), 3.46 (s, 2H, C_Ar_−C*H*_2_−N), 3.40–3.32 (m, 1H, C*H*‐N_3_), 2.78–2.71 (m, 2H), 2.26 (d, *J*=1.1 Hz, 3H, C_Ar_−C*H*_3_), 2.18–2.10 (m, 2H), 1.91–1.83 (m, 2H), 1.69–1.60 (m, 2H). ^**13**^ **C NMR (151 MHz, CDCl_3_) δ** 144.0, 142.2, 137.4, 133.4, 131.0, 130.4, 129.7, 129.1, 129.1, 128.9, 127.9, 122.0, 121.0, 120.8, 117.0, 62.8 (C_Ar_−*C*H_2_−N), 58.0 (*C*H−N_3_), 51.3, 31.0, 18.4 (C_Ar_−*C*H_3_). **HRMS (TOF MS ESI+)**: *m/z* calcd for C_26_H_27_N_6_^+^ [M+H]^+^, 423.2297, found 423.2303, error 1.4 ppm.

***N*** **‐(2‐Ethynylphenyl)acrylamide (51)**. Following a literature procedure,^[6a]^ 2‐ethynylaniline **48** (0.20 mL, 0.21 g, 1.8 mmol), DCM (15 mL) and Et_3_N (2.4 equiv, 0.60 mL, 0.44 g, 4.3 mmol) were cooled to 0 °C by means of an ice bath, followed by dropwise addition of acryloyl chloride (2.5 equiv, 0.35 mL, 0.39 g, 4.3 mmol). The mixture was then stirred at room temperature for 27 h and subsequently diluted with DCM (200 mL). The organic layer was washed with saturated aqueous NaHCO_3_ (4×50 mL), dried over MgSO_4_ and filtered. The solvent was removed *in vacuo* to yield the crude product which was purified by column chromatography utilizing the CombiFlash system (Hex to 20 % EtOAc/hexane) to afford **51** (0.283 g, 1.65 mmol, 94 %) R_f_=0.35, 20 % EtOAc/hexane) as a pale orange solid. ^**1**^ **H NMR (400 MHz, CDCl_3_) δ** 8.51 (d, *J*=8.6 Hz, 1H), 8.06 (br s, 1H), 7.47 (dd, *J*=7.7, 1.6 Hz, 1H), 7.38 (ddd, *J*=8.6, 7.6, 1.6 Hz, 1H), 7.06 (app. td, ddd, *J*=7.7, 7.6, 1.2 Hz, 1H), 6.50 (d, *J*=7.0 Hz, H_rotamer_), 6.48 (d, *J*=2.4 Hz, H_rotamer_), 6.43 (dd, *J*=16.9, 1.2 Hz, 1H), 6.29 (dd, *J*=16.9, 10.2 Hz, 1H), 5.80 (dd, *J*=10.2, 1.2 Hz, 1H), 5.74 (d, *J*=7.0, 2.4 Hz, H_rotamer_), 3.53 (s, 1H, C≡C−*H*). ^**13**^ **C NMR (101 MHz, CDCl_3_) δ** 163.5 (*C*=O), 139.6, 132.3, 131.5, 130.4, 128.0, 123.7, 119.6, 111.0, 84.7, 79.3. Its characterisation corresponded to the literature.^[31]^

***N*** **‐(3‐Ethynylphenyl)acrylamide (52). 52** was synthesised following a literature procedure and its characterisation corresponded to the same literature.^[6a]^

Compound **53** was prepared by a method similar to that for compound **51**.

***N*** **‐(4‐Ethynylphenyl)acrylamide (53)**. White solid (0.182 g, 1.06 mmol, 61 %) (R_f_=0.63, 50 % EtOAc/hexane). **Mp** 142–144 °C. **IR (ATR, cm^−1^)**: 3297 (alkyne C−H str, m), 3186 (w), 3106 (m), 3064 (w), 2161 (C≡C str, w), 2103 (w), 1662 (C=O str, s), 1633 (m), 1601 (s), 1536 (s), 1506 (s), 1413 (s), 1333 (C‐N str, s), 1288 (m), 1251 (m). ^**1**^ **H NMR (400 MHz, CDCl_3_) δ** 7.60–7.53 (m, 3H), 7.49–7.41 (m, 2H), 6.44 (dd, *J*=16.9, 1.2 Hz, 1H), 6.25 (dd, *J*=16.9, 10.2 Hz, 1H), 5.78 (dd, *J*=10.2, 1.2 Hz, 1H), 3.05 (s, 1H, C≡C‐*H*). ^**13**^ **C NMR (101 MHz, CDCl_3_) δ** 163.7 (*C*=O), 138.3, 133.1, 131.0, 128.5, 119.7, 118.1, 83.4, 77.1. **HRMS (TOF MS ESI+)**: *m/z* calcd for C_11_H_10_NO^+^ [M+H]^+^, 172.0762, found 172.0761, error 0.6 ppm.

***N*** **‐[3‐(1‐{1‐[4‐(3‐Phenylimidazo[1,2‐*a*]pyridin‐2‐yl)benzyl]piperidin‐4‐yl}‐1*H*‐1,2,3‐triazol‐4‐yl)phenyl]acrylamide (3)**. Material was used as previously made in a literature procedure.^[6a]^

***N*** **‐[2‐(1‐{1‐[4‐(3‐Phenylimidazo[1,2‐*a*]pyridin‐2‐yl)benzyl]piperidin‐4‐yl}‐1*H*‐1,2,3‐triazol‐4‐yl)phenyl]acrylamide (4)**. Following a literature procedure,^[6a]^ as for the synthesis of **3**, 2‐{4‐[(4‐azidopiperidin‐1‐yl)methyl]phenyl}‐3‐phenylimidazo[1,2‐*a*]pyridine **42** (0.043 g, 0.11 mmol), *N*‐(2‐ethynylphenyl)acrylamide **51** (1.7 equiv, 0.031 g, 0.18 mmol), *N*,*N*‐diisopropylethylamine (2.7 equiv, 0.050 mL, 0.037 g, 0.29 mmol) and MeCN (1 mL) were heated at 45 °C to dissolve the contents. The reaction mixture was then allowed to cool to room temperature before CuI (38 mol%, 8 mg, 0.04 mmol) was added. The reaction was stirred at room temperature for 24 h and more MeCN (1 mL) was added after the first 4.5 h. The reaction mixture was then heated to 25 °C and more CuI (20 mol%, 4 mg, 0.02 mmol) was added. It was stirred at 25 °C for 3 days and turned a light orange colour with time. The reaction mixture was diluted with DCM, filtered through a cotton wool plug and concentrated *in vacuo*. The resulting orange crude material was purified by CC (2.5 % MeOH/DCM) to afford the still impure **4** which was then further purified by a second round of CC (2.5 % MeOH/DCM) to afford **4** (0.022 g, 0.038 mmol, 36 %) (R_f_=0.54, 5 % MeOH/DCM) as a pale yellow solid. **Mp** 114–116 °C. **IR (ATR, cm^−1^)**: 3074, 2943, 1684 (C=O str), 1615 (Ar C=C str), 1589, 1540, 1476 (Ar C=C str), 1448 (−CH_2_− bend), 1399, 1344, 1320, 1270 (C−N str). ^**1**^ **H NMR (400 MHz, CDCl_3_) δ** 11.68 (s, 1H), 8.76 (d, *J*=7.7 Hz, 1H), 7.95 (dt, *J*=6.9, 1.2 Hz, 1H), 7.89 (s, 1H), 7.73 (dt, *J*=9.1, 1.2 Hz, 1H), 7.65 (d, 8.6 Hz, 2H), 7.57–7.44 (m, 6H), 7.35 (t, *J*=8.0 Hz, 1H), 7.25 (d, *J*=8.6 Hz, 2H), 7.21 (ddd, *J*=9.1, 6.8, 1.3 Hz, 1H), 7.10 (td, *J*=7.7, 1.2 Hz, 1H), 6.76 (td, *J*=6.9, 1.3 Hz, 1H), 6.44 (d, *J*=2.5 Hz, 1H), 6.43 (d, *J*=9.1 Hz, 1H), 5.76 (dd, *J*=9.1, 2.5 Hz, 1H), 4.57–4.48 (m, 1H), 3.55 (s, 2H), 3.06 (d, *J*=9.8 Hz, 2H), 2.27–2.12 (m, 6H). ^**13**^ **C NMR (100 MHz, CDCl_3_) δ** 164.2 (C=O), 147.4, 144.7, 141.9, 137.1, 136.5, 133.0, 132.7, 130.7, 130.4, 129.6, 129.5, 129.0, 128.9, 128.9, 128.0, 126.8, 126.5, 124.9, 123.4, 123.2, 121.4, 121.0, 118.5, 117.4, 112.4, 62.3, 58.7, 52.0, 32.6. **HRMS**: calcd for C_36_H_34_N_7_O^+^ [M+H]^+^, 580.2825, found 580.2831, error 1.0 ppm.

Compounds **5**–**10** were prepared by a method similar to that for compounds **3** and **4**.

***N*** **‐[4‐(1‐{1‐[4‐(3‐Phenylimidazo[1,2‐*a*]pyridin‐2‐yl)benzyl]piperidin‐4‐yl}‐1*H*‐1,2,3‐triazol‐4‐yl)phenyl]acrylamide (5)**. Pale yellow solid (0.117 g, 0.203 mmol, 85 %) (R_f_=0.19, 5 % MeOH/DCM). Decompn. ∼215 °C. IR (ATR, cm^−1^): 3271 (N−H str, s), 3045 (m), 2938 (m), 1661 (C=O str, s), 1625 (m), 1547 (m), 1513 (m), 1342 (C‐N str, s), 1239 (s). ^1^H NMR (600 MHz, DMSO‐d_6_) δ 10.22 (s, 1H), 8.61 (s, 1H), 8.00 (d, *J*=6.8 Hz, 1H), 7.79 (d, *J*=8.7 Hz, 2H), 7.74 (d, *J*=8.7 Hz, 2H), 7.67 (d, *J*=9.1 Hz, 1H), 7.63–7.53 (m, 5H), 7.52–7.48 (m, 2H), 7.32 (ddd, *J*=9.1, 6.7, 1.3 Hz, 1H), 7.25 (d, *J*=7.9 Hz, 2H), 6.90 (app. td, ddd, *J*=6.8, 6.7, 1.2 Hz, 1H), 6.45 (dd, *J*=17.0, 10.1 Hz, 1H), 6.27 (dd, *J*=17.0, 1.9 Hz, 1H), 5.77 (dd, *J*=10.1, 1.9 Hz, 1H), 4.55–4.48 (m, 1H), 3.50 (s, 2H), 2.91 (d, *J*=11.2 Hz, 2H), 2.18 (app. t, dd, *J*=11.3, 11.3 Hz, 2H), 2.12–1.99 (m, 4H). ^13^C NMR (151 MHz, DMSO‐d_6_) δ 163.1 (C=O), 146.0, 144.0, 141.1, 138.6, 137.7, 132.8, 131.8, 130.7, 129.7, 129.4, 129.1, 128.6, 127.3, 126.9, 126.2, 125.6, 125.2, 123.7, 122.0, 119.6, 119.0, 116.8, 112.7, 61.4, 57.5, 51.6, 32.1. HRMS (TOF MS ESI+): *m/z* calcd for C_36_H_33_N_7_O^+^ [M+H]^+^, 580.2825, found 580.2819, error 1.0 ppm. Purity 94.5 %.

***N*** **‐{2‐[1‐(1‐{4‐[3‐(*o*‐Tolyl)imidazo[1,2‐*a*]pyridin‐2‐yl]benzyl}piperidin‐4‐yl)‐1*H*‐1,2,3‐triazol‐4‐yl]phenyl}acrylamide (6)**. Off‐white solid (0.040 g, 0.067 mmol, 66 %) (R_f_=0.53, 5 % MeOH/EtOAc). Mp 128–130 °C. IR (ATR, cm^−1^): 3258 (w), 3059 (w), 2942 (w), 2801 (w), 1682 (C=O str, s), 1615 (m), 1589 (s), 1538 (m), 1474 (w), 1448 (s), 1400 (w), 1340 (C‐N str, m), 1319 (m), 1283 (m). ^1^H NMR (300 MHz, CDCl_3_) δ 11.67 (s, 1H), 8.75 (dd, *J*=8.0, 1.2 Hz, 1H), 7.87 (s, 1H), 7.77 (app. dt, ddd, *J*=9.1, 1.2, 1.2 Hz, 1H), 7.69–7.60 (m, 2H), 7.56 (app. dt, ddd, *J*=6.8, 1.2, 1.2 Hz, 1H), 7.50–7.30 (m, 6H), 7.25–7.19 (m, 3H), 7.10 (app. td, ddd, *J*=8.0, 7.6, 1.3 Hz, 1H), 6.74 (app. td, ddd, *J*=6.8, 6.8, 1.2 Hz, 1H), 6.49 (d, *J*=2.9 Hz, H_rotamer_), 6.43 (d, *J*=2.9 Hz, 1H), 6.42 (d, *J*=8.6 Hz, 1H), 6.36 (d, *J*=8.6 Hz, H_rotamer_), 5.75 (dd, *J*=8.6, 2.9 Hz, 1H), 4.58–4.45 (m, 1H), 3.52 (s, 2H), 3.08–2.98 (m, 2H), 2.27–2.05 (m, 6H), 2.01 (s, 3H, C_Ar_−C*H*_3_). ^13^C NMR (75 MHz, CDCl_3_) δ 164.4 (C=O), 147.6, 144.9, 141.8, 139.3, 137.4, 136.7, 133.2, 132.9, 131.9, 131.1, 129.9, 129.2, 129.1, 129.1, 127.3, 127.1, 127.0, 126.7, 125.0, 123.6, 123.5, 121.6, 120.4, 118.7, 117.6, 117.6, 112.6, 62.5, 59.0, 52.2, 32.8, 19.5 (C_Ar_−*C*H_3_). HRMS (TOF MS ESI+): *m/z* calcd for C_37_H_36_N_7_O^+^ [M+H]^+^, 594.2981, found 594.2991, error 1.7 ppm. Purity 98.8 %.

***N*** **‐{3‐[1‐(1‐{4‐[3‐(*o*‐Tolyl)imidazo[1,2‐*a*]pyridin‐2‐yl]benzyl}piperidin‐4‐yl)‐1*H*‐1,2,3‐triazol‐4‐yl]phenyl}acrylamide (7)**. White solid (19 mg, 0.033 mmol, 29 %) (R_f_=0.46, 5 % MeOH/EtOAc). Mp 124–126 °C. IR (ATR, cm^−1^): 3463 (w), 3267 (m), 3212 (w), 3076 (w), 2934 (m), 2801 (w), 1668 (C=O str, s), 1615 (s), 1568 (s), 1532 (m), 1480 (m), 1444 (s), 1410 (m), 1340 (C‐N str, s), 1237 (s). ^1^H NMR (600 MHz, CDCl_3_) δ 8.32 (s, 1H), 8.05 (s, 1H), 7.75 (s, 1H), 7.73 (d, *J*=9.0 Hz, 1H), 7.65–7.60 (m, 1H), 7.63 (d, *J*=7.8 Hz, 2H), 7.59 (d, *J*=7.7 Hz, 1H), 7.56 (d, *J*=6.8 Hz, 1H), 7.47–7.39 (m, 2H), 7.38–7.30 (m, 3H), 7.24–7.18 (m, 1H), 7.20 (d, *J*=7.8 Hz, 2H), 6.73 (app. t, dd, *J*=6.7, 6.7 Hz, 1H), 6.42 (d, *J*=16.8 Hz, 1H), 6.33 (dd, *J*=16.8, 10.1 Hz, 1H), 5.71 (d, *J*=10.1 Hz, 1H), 4.44 (app. tt, dddd, *J*=11.7, 11.7, 4.2, 4.2 Hz, 1H), 3.48 (s, 2H), 2.96 (d, *J*=11.4 Hz, 2H), 2.18–2.11 (m, 4H), 2.08–2.01 (m, 2H), 2.00 (s, 3H, C_Ar_−C*H*_3_). ^13^C NMR (151 MHz, CDCl_3_) δ 164.0 (C=O), 147.2, 144.8, 141.9, 139.2, 138.7, 137.6, 133.2, 131.9, 131.5, 131.4, 131.1, 129.9, 129.7, 129.1, 127.8, 127.3, 127.1, 124.9, 123.5, 121.7, 120.4, 119.7, 117.8, 117.5, 117.2, 112.6, 62.5, 58.5, 52.2, 32.8, 19.5 (C_Ar_−*C*H_3_). HRMS (TOF MS ESI+): *m/z* calcd for C_37_H_36_N_7_O^+^ [M+H]^+^, 594.2981, found 594.2982, error 0.2 ppm. Purity 99.9 %.

***N*** **‐{4‐[1‐(1‐{4‐[3‐(*o*‐Tolyl)imidazo[1,2‐*a*]pyridin‐2‐yl]benzyl}piperidin‐4‐yl)‐1*H*‐1,2,3‐triazol‐4‐yl]phenyl}acrylamide (8)**. Yellow solid (0.045 g, 0.075 mmol, 71 %) (R_f_=0.33, 5 % MeOH/EtOAc). Mp 169–170 °C. IR (ATR, cm^−1^): 3465 (m), 3429 (s), 3263 (s), 3109 (w), 2947 (m), 2811 (w), 2257 (w), 2167 (w), 2135 (w), 2037 (w), 1737 (w), 1668 (C=O str, s), 1633 (w), 1595 (s), 1531 (s), 1497 (s), 1441 (w), 1412 (s), 1341 (C−N str, s), 1244 (m). ^1^H NMR (600 MHz, CDCl_3_&MeOD‐d_4_) δ 8.64 (s, 1H), 7.78 (s, 1H), 7.71–7.64 (m, 5H), 7.59 (d, *J*=8.1 Hz, 2H), 7.55 (dd, *J*=6.9, 1.2 Hz, 1H), 7.44 (app. td, ddd, *J*=7.5, 7.2, 1.6 Hz, 1H), 7.40 (d, *J*=7.3 Hz, 1H), 7.34 (app. td, ddd, *J*=7.3, 7.2, 1.6 Hz, 1H), 7.31 (dd, *J*=7.5, 1.6 Hz, 1H), 7.24–7.21 (m, 1H), 7.21 (d, *J*=8.1 Hz, 2H), 6.74 (app. td, ddd, *J*=6.9, 6.8, 1.2 Hz, 1H), 6.40 (dd, *J*=16.9, 1.9 Hz, 1H), 6.34 (dd, *J*=16.9, 9.7 Hz, 1H), 5.71 (dd, *J*=9.7, 1.9 Hz, 1H), 4.47 (app. tt, dddd, *J*=11.5, 11.5, 4.4, 4.4 Hz, 1H), 3.51 (s, 2H), 2.99 (d, *J*=11.2 Hz, 2H), 2.22–2.12 (m, 4H), 2.12–2.02 (m, 2H), 1.99 (s, 3H, C_Ar_−C*H*_3_). ^13^C NMR (151 MHz, CDCl_3_&MeOD‐d_4_) δ 164.1 (C=O), 147.2, 144.8, 141.7, 139.2, 138.1, 137.0, 133.2, 131.9, 131.2, 131.1, 129.9, 129.4, 129.0, 127.7, 127.3, 127.1, 126.6, 126.3, 125.1, 123.6, 120.5, 120.3, 117.5, 117.2, 112.7, 62.5, 58.4, 52.2, 32.6, 19.5 (C_Ar_−*C*H_3_). HRMS (TOF MS ESI+): *m/z* calcd for C_37_H_36_N_7_O^+^ [M+H]^+^, 594.2981, found 594.2991, error 1.7 ppm. Purity 92.4 %.

***N*** **‐{3‐[1‐(1‐{4‐[3‐(*m*‐Tolyl)imidazo[1,2‐*a*]pyridin‐2‐yl]benzyl}piperidin‐4‐yl)‐1*H*‐1,2,3‐triazol‐4‐yl]phenyl}acrylamide (9)**. Yellow solid (15 mg, 0.025 mmol, 31 %) (R_f_=0.48, 5 % MeOH/EtOAc). Mp 130–132 °C. IR (ATR, cm^−1^): 3564 (m), 1674 (C=O str, s), 1614 (s), 1532 (m), 1479 (m), 1444 (s), 1414 (s), 1338 (C=N str, s), 1248 (m). ^1^H NMR (600 MHz, CDCl_3_) δ 8.10 (s, 1H), 8.05 (s, 1H), 7.93 (d, *J*=6.8 Hz, 1H), 7.77 (s, 1H), 7.73 (d, *J*=9.1 Hz, 1H), 7.66–7.62 (m, 1H), 7.64 (d, *J*=7.9 Hz, 2H), 7.62–7.58 (m, 1H), 7.40 (app. t, dd, *J*=7.7, 7.6 Hz, 1H), 7.34 (app. t, dd, *J*=7.9, 7.9 Hz, 1H), 7.29 (d, *J*=7.7 Hz, 1H), 7.26–7.18 (m, 3H), 7.22 (d, *J*=7.9 Hz, 2H), 6.75 (app. t, dd, *J*=6.8, 6.7 Hz, 1H), 6.43 (dd, *J*=16.8, 1.4 Hz, 1H), 6.32 (dd, *J*=16.8, 10.1 Hz, 1H), 5.73 (dd, *J*=10.1, 1.4 Hz, 1H), 4.46 (app. tt, dddd, *J*=11.5, 11.5, 4.2, 4.2 Hz, 1H), 3.51 (s, 2H), 3.01–2.95 (m, 2H), 2.39 (s, 3H, C_Ar_−C*H*_3_), 2.20–2.11 (m, 4H), 2.10–2.00 (m, 2H). ^13^C NMR (151 MHz, CDCl_3_) δ 163.9 (C=O), 147.2, 144.8, 142.0, 139.5, 138.7, 137.6, 133.0, 131.6, 131.4, 131.3, 130.0, 129.7, 129.6, 129.6, 129.0, 128.2, 128.0, 127.8, 125.1, 123.6, 121.7, 121.4, 119.7, 117.8, 117.5, 117.1, 112.6, 62.5, 58.6, 52.3, 32.8, 21.6 (C_Ar_−*C*H_3_). HRMS (TOF MS ESI+): *m/z* calcd for C_37_H_36_N_7_O^+^ [M+H]^+^, 594.2981, found 594.2988, error 1.2 ppm. Purity 93 %.

***N*** **‐[3‐(1‐{1‐[4‐(6‐Methyl‐3‐phenylimidazo[1,2‐*a*]pyridin‐2‐yl)benzyl]piperidin‐4‐yl}‐1*H*‐1,2,3‐triazol‐4‐yl)phenyl]acrylamide (10)**. Pale yellow solid (0.028 g, 0.047 mmol, 40 %) (R_f_=0.23, 5 % MeOH/EtOAc). Mp 217–219 °C. IR (ATR, cm^−1^): 3400 (N−H str, w), 2921 (m), 2291 (m), 1679 (C=O str, s), 1614 (m), 1561 (w), 1536 (m), 1513 (m), 1488 (s), 1450 (m), 1412 (s), 1389 (m), 1377 (m), 1336 (C‐N str, m), 1316 (m), 1290 (w), 1200 (m). ^1^H NMR (400 MHz, CDCl_3_&MeOD‐d_4_) δ 8.03 (s, 1H), 7.81 (s, 1H), 7.73–7.67 (m, 1H), 7.65–7.49 (m, 8H), 7.48–7.41 (m, 3H), 7.39 (app. t, dd, *J*=7.9, 7.9 Hz, 1H), 7.24 (d, *J*=8.2 Hz, 2H), 7.05 (dd, *J*=9.2, 1.7 Hz, 1H), 6.45 (dd, *J*=16.8, 1.3 Hz, 1H), 6.27 (dd, *J*=16.8, 10.2 Hz, 1H), 5.79 (dd, *J*=10.2, 1.3 Hz, 1H), 4.56–4.46 (m, 1H), 3.54 (s, 2H), 3.07–3.00 (m, 2H), 2.27 (s, 3H, C_Ar_−C*H*_3_), 2.24–2.16 (m, 4H), 2.16–2.05 (m, 2H). ^13^C NMR (75 MHz, CDCl_3_&MeOD‐d_4_) δ 164.4 (C=O), 147.2, 143.9, 141.7, 139.2, 136.9, 133.2, 131.3, 130.9, 130.8, 129.9, 129.7, 129.7, 129.2, 129.0, 128.4, 128.0, 127.6, 122.3, 121.2, 121.0, 121.0, 119.9, 118.0, 116.5, 116.5, 62.5, 58.6, 52.2, 32.5, 18.4 (C_Ar_−*C*H_3_). HRMS (TOF MS ESI+): *m/z* calcd for C_37_H_36_N_7_O^+^ [M+H]^+^, 594.2981, found 594.2985, error 0.7 ppm. Purity ∼98 %.

**4‐(5‐Chloro‐6‐nitro‐2‐oxo‐2,3‐dihydro‐1*H*‐benzo[*d*]imidazol‐1‐yl)‐piperidin‐1‐ium nitrate (55)**. Following a literature procedure,^[6a]^ 5‐chloro‐1‐(piperidine‐4‐yl)‐1,3‐dihydro‐2*H*‐benzo[*d*]imidazol‐2‐one **54** (0.258 g, 1.02 mmol), toluene (5 mL) and 55 % HNO_3_ (11 equiv, 1.25 mL, 0.688 g, 10.9 mmol) were stirred at 60 °C for 2.5 h. The mixture was cooled in an ice bath and diluted with H_2_O (20 mL). The resulting yellow precipitate was filtered through a sinter funnel, washed with cold H_2_O and solvent was removed *in vacuo* to afford **55** (0.355 g, 0.986 mmol, 96 %) as a pale‐yellow solid with no further purification required. **Decompn**. 220–222 °C. **IR (ATR, cm^−1^)**: 3 487 (N−H str, w), 3433 (w), 3075 (w), 3065 (w), 3003 (m), 2839 (m), 1692 (C=O str, s), 1607 (w), 1525 (nitro str, m), 1494 (m), 1458 (m), 1412 (m), 1383 (s), 1326 (nitro str, s), 1297 (s). ^**1**^ **H NMR (600 MHz, DMSO‐d_6_) δ** 11.75 (s, 1H), 8.59 (d, *J*=11.3 Hz, 1H), 8.35 (d, *J*=11.3 Hz, 1H), 8.06 (s, 1H), 7.27 (s, 1H), 4.58 (app. tt, dddd, *J*=12.3, 12.3, 4.2, 4.2 Hz, 1H), 3.45 (d, *J*=12.4 Hz, 2H), 3.09 (d, *J*=12.4 Hz, 1H), 3.05 (d, *J*=12.4 Hz, 1H), 2.55–2.45 (m, 2H), 1.95–1.89 (m, 2H). ^**13**^ **C NMR (151 MHz, DMSO‐d_6_)** δ 153.8 (C=O), 140.7, 133.1, 128.1, 119.3, 110.6, 106.1, 47.7, 43.1, 25.2. **HRMS (TOF MS ESI+)**: *m/z* calcd for C_12_H_14_ClN_4_O_3_^+^ [M+H]^+^, 297.0754, found 297.0754 (thus without NO_3_^−^), error 0 ppm.

**6‐Amino‐1‐(piperidin‐4‐yl)‐1,3‐dihydro‐2*H*‐benzo[*d*]imidazol‐2‐one (56)**. Following a literature procedure,^[6a]^ 4‐(5‐chloro‐6‐nitro‐2‐oxo‐2,3‐dihydro‐1*H*‐benzo[*d*]imidazol‐1‐yl)piperidin‐1‐ium nitrate **55** (0.396 g, 1.10 mmol), 10 % Pd/C (0.039 g) and EtOH (8 mL) were stirred at 80 °C for 2 h and then cooled to room temperature. Ammonium formate (12 equiv, 0.80 g, 13 mmol) was added and the reaction mixture was stirred at 80 °C for a further 7 h after which it allowed to cool to room temperature, filtered through celite and concentrated *in vacuo*. The yellow semi‐solid was run through a short silica plug (10 % MeOH/DCM) and the solvent was removed to yield the product **56** (0.203 g, 0.876 mmol, 79 %) (R_f_=0.03, 15 % MeOH/DCM) as a yellow solid which did not require further purification. **Mp** 128–130 °C. **IR (ATR, cm^−1^)**: 3038 (N−H str, s), 2839 (m), 2518 (w), 1672 (C=O str, s), 1631 (s), 1500 (m), 1473 (m), 1456 (m), 1373 (s), 1304 (C‐N str, s), 1218 (m). ^**1**^ **H NMR (400 MHz, DMSO‐d_6_) δ** 10.47 (s, 1H), 8.93 (br s, 2H, C_Ar_−N*H*_2_), 6.74 (d, *J*=2.0 Hz, 1H), 6.71 (d, *J*=8.2 Hz, 1H), 6.36 (dd, *J*=8.2, 2.0 Hz, 1H), 5.45 (br s, 1H), 4.38 (app. tt, dddd, *J*=12.2, 12.2, 4.1, 4.1 Hz, 1H), 3.45–3.31 (m, 2H), 3.12–3.00 (m, 2H), 2.66–2.53 (m, 2H), 1.81 (br d, *J*=14.0 Hz, 2H). ^**13**^ **C NMR (101 MHz, DMSO‐d_6_) δ** 153.8 (C=O), 141.0, 129.8, 120.2, 109.3, 108.2, 96.6, 47.0, 43.0, 25.3. **HRMS (TOF MS ESI+)**: *m/z* calcd for C_12_H_17_N_4_O^+^ [M+H]^+^, 233.1402, found 233.1400, error 0.9 ppm.

***tert*** **‐Butyl [2‐oxo‐3‐(piperidin‐4‐yl)‐2,3‐dihydro‐1*H*‐benzo[*d*]imidazol‐5‐yl]carbamate (57)**. Following a literature procedure,^[6a]^ 6‐amino‐1‐(piperidin‐4‐yl)‐1,3‐dihydro‐2*H*‐benzo[*d*]imidazol‐2‐one **56** (0.100 g, 0.431 mmol) and 10 % AcOH in H_2_O (4 mL) were added together. Boc_2_O (1.0 equiv, 0.10 mL, 0.095 g, 0.43 mmol) in 1,4‐dioxane (4 mL) was then added to the mixture in a dropwise manner. The reaction mixture was stirred at room temperature for 18 h and then basified with 2 M NaOH to pH 10 as indicated by universal indicator paper. It was extracted with 10 % MeOH/DCM (8×25 mL) and the combined organic extracts were dried over MgSO_4_ and filtered. The solvent was removed *in vacuo* to yield the crude product which was purified by column chromatography (10 % MeOH/DCM) to afford **57** (0.041 g, 0.12 mmol, 29 %) (R_f_=0.08, 10 % MeOH/DCM) as a yellow solid. **Mp** 146–148 °C. ^**1**^ **H NMR (400 MHz, CDCl_3_) δ** 7.54 (s, 1H), 6.98 (dd, *J*=8.4, 2.0 Hz, 1H), 6.86 (dd, *J*=8.4, 1.9 Hz, 1H), 4.36 (app. tt, dddd, *J*=12.4, 12.4, 4.3, 4.3 Hz, 1H), 3.31 (d, *J*=13.1 Hz, 2H), 2.81 (app. t, dd, *J*=13.1, 11.3 Hz, 2H), 2.55–2.42 (m, 2H), 1.82 (d, *J*=12.8 Hz, 2H), 1.51 (d, *J*=2.3 Hz, 9H, −C(C*H*_3_)_3_). **HRMS (TOF MS ESI+)**: *m/z* calcd for C_17_H_25_N_4_O_3_^+^ [M+H]^+^, 333.1927, found 333.1918, error 2.7 ppm.

Compound **58** was prepared by a method similar to that for compound **42**.

***tert*** **‐Butyl (2‐oxo‐3‐{1‐[4‐(3‐phenylimidazo[1,2‐*a*]pyridin‐2‐yl)benzyl]piperidin‐4‐yl}‐2,3‐dihydro‐1*H*‐benzo[*d*]imidazol‐5‐yl)carbamate (58)**. Yellow solid (0.055 g, 0.089 mmol, 52 %) (R_f_=0.40, 10 % MeOH/DCM). Mp 196–198 °C. IR (ATR, cm^−1^): 3243 (N−H str, m), 2931 (m), 1686 (C=O str, s), 1634 (m), 1500 (s), 1453 (m), 1365 (s), 1344 (m), 1270 (w), 1239 (s). ^1^H NMR (400 MHz, CDCl_3_) δ 9.30 (s, 1H), 7.94 (app. dt, ddd, *J*=6.9, 1.2, 1.2 Hz, 1H), 7.70 (app. dt, ddd, *J*=9.1, 1.2, 1.2 Hz, 1H), 7.69–7.62 (m, 2H), 7.59–7.43 (m, 5H), 7.37 (s, 1H), 7.32–7.25 (m, 2H), 7.20 (ddd, *J*=9.1, 6.7, 1.2 Hz, 1H), 6.99–6.91 (m, 2H), 6.73 (app. td, ddd, *J*=6.9, 6.7, 1.2 Hz, 1H), 6.59 (s, 1H), 4.36–4.25 (m, 1H), 3.57 (s, 2H), 3.05 (d, *J*=11.3 Hz, 2H), 2.55–2.42 (m, 2H), 2.18 (app. t, dd, *J*=11.9, 11.6 Hz, 2H), 1.76 (br d, *J*=11.9 Hz, 2H), 1.53 (s, 9H, −C(C*H*_3_)_3_). ^13^C NMR (151 MHz, CDCl_3_) δ 155.3 (C=O), 153.4 (C=O), 145.0, 142.4, 137.7, 133.2, 132.6, 130.9, 130.1, 129.8, 129.7, 129.2, 129.1, 128.0, 124.8, 124.1, 123.4, 121.2, 121.1, 117.7, 112.9, 112.4, 109.5, 80.6 (−*C*(CH_3_)_3_), 62.6, 53.1, 51.1, 29.2, 28.6 (−C(*C*H_3_)_3_). HRMS (TOF MS ESI+): *m/z* calcd for C_37_H_39_N_6_O_3_^+^ [M+H]^+^, 615.3084, found 615.3064, error 3.3 ppm.

Compound **59** was prepared by a method similar to that for compound **40**.

**6‐Amino‐1‐{1‐[4‐(3‐phenylimidazo[1,2‐*a*]pyridin‐2‐yl)benzyl]piperidin‐4‐yl}‐1,3‐dihydro‐2*H*‐benzo[*d*]imidazol‐2‐one (59)**. Brown solid (19 mg, 0.037 mmol, 51 %) (R_f_=0.26, 10 % MeOH/DCM). Decompn. ∼220 °C. ^1^H NMR (300 MHz, CDCl_3_) δ 9.83 (s, 1H), 7.93 (app. dt, ddd, *J*=6.9, 1.2, 1.2 Hz, 1H), 7.70 (app. dt, ddd, *J*=9.1, 1.2, 1.2 Hz, 1H), 7.68–7.62 (m, 2H), 7.60–7.41 (m, 5H), 7.30–7.25 (m, 2H), 7.19 (ddd, *J*=9.1, 6.8, 1.2 Hz, 1H), 6.79 (d, *J*=8.2 Hz, 1H), 6.72 (app. td, ddd, *J*=6.9, 6.8, 1.2 Hz, 1H), 6.68 (d, *J*=2.0 Hz, 1H), 6.36 (dd, *J*=8.2, 2.0 Hz, 1H), 4.32 (app. tt, dddd, *J*=12.3, 12.3, 4.1, 4.1 Hz, 1H), 3.56 (s, 2H), 3.05 (d, *J*=11.1 Hz, 2H), 2.43 (app. qd, dddd, *J*=12.6, 12.6, 12.5, 3.8 Hz, 2H), 2.24–2.10 (m, 2H), 1.76 (dd, *J*=13.2, 4.3 Hz, 2H). ^13^C NMR (75 MHz, CDCl_3_) δ 155.5 (C=O), 144.9, 142.3, 141.2, 137.3, 133.2, 130.9, 130.2, 130.0, 129.7, 129.2, 129.0, 128.0, 124.8, 123.4, 121.1, 121.0, 117.6, 112.4, 110.2, 108.6, 98.0, 62.8, 53.2, 50.6, 29.2. HRMS (TOF MS ESI+): *m/z* calcd for C_32_H_31_N_6_O^+^ [M+H]^+^, 515.2559, found 515.2556, error 0.6 ppm.

Compound **11** was prepared by a method similar to that for compound **52**.

***N*** **‐(2‐Oxo‐3‐{1‐[4‐(3‐phenylimidazo[1,2‐*a*]pyridin‐2‐yl)benzyl]piperidin‐4‐yl}‐2,3‐dihydro‐1*H*‐benzo[*d*]imidazol‐5‐yl)acrylamide (11)**. Yellow solid (13 mg, 0.022 mmol, 56 %) (R_f_=0.28, 10 % MeOH/DCM). Mp 221–223 °C. IR (ATR, cm^−1^): 3077 (w), 2927 (w), 2804 (w), 1682 (C=O str, s), 1616 (m), 1558 (w), 1496 (s), 1465 (m), 1378 (m), 1343 (m), 1297 (w), 1273 (w), 1235 (m). ^1^H NMR (300 MHz, CDCl_3_) δ 9.99 (s, 1H), 8.46 (s, 1H), 7.95 (app. dt, ddd, *J*=6.9, 1.2, 1.2 Hz, 1H), 7.78–7.72 (m, 1H), 7.72 (s, 1H), 7.63 (d, *J*=8.2 Hz, 2H), 7.59–7.40 (m, 5H), 7.27–7.22 (m, 2H), 7.20 (ddd, *J*=9.1, 6.8, 1.2 Hz, 1H), 7.11 (br d, *J*=8.4 Hz, 1H), 6.77 (d, *J*=8.4 Hz, 1H), 6.74 (app. td, ddd, *J*=6.9, 6.8, 1.1 Hz, 1H), 6.40 (dd, *J*=16.9, 2.1 Hz, 1H), 6.30 (dd, *J*=16.9, 9.6 Hz, 1H), 5.65 (dd, *J*=9.6, 2.1 Hz, 1H), 4.37–4.16 (m, 1H), 3.57 (s, 2H), 3.02 (d, *J*=11.0 Hz, 2H), 2.56–2.38 (m, 2H), 2.22–2.08 (m, 2H), 1.71 (d, *J*=12.3 Hz, 2H). ^13^C NMR (75 MHz, CDCl_3_) δ 164.0 (C=O), 155.3 (C=O), 145.0, 142.2, 136.6, 133.3, 132.3, 131.5, 130.9, 129.8, 129.7, 129.6, 129.5, 129.1, 128.2, 127.4, 125.0, 123.5, 121.2, 117.6, 113.9, 112.5, 109.5, 103.0, 62.5, 53.0, 50.9, 28.9. HRMS (TOF MS ESI+): *m/z* calcd for C_35_H_33_N_6_O_2_^+^ [M+H]^+^, 569.2665, found 569.2667, error 0.4 ppm. Purity ∼98 %.

Compound **60** was prepared by a method similar to that for compound **42**.

**5‐Chloro‐6‐nitro‐1‐{1‐[4‐(3‐phenylimidazo[1,2‐*a*]pyridin‐2‐yl)benzyl]piperidin‐4‐yl}‐1,3‐dihydro‐2*H*‐benzo[*d*]imidazol‐2‐one (60)**. Bright yellow solid (0.369 g, 0.637 mmol, 66 %) (R_f_=0.34, 10 % MeOH/DCM). Mp 198–199 °C. IR (ATR, cm^−1^): 3071 (m), 2927 (m), 2816 (m), 1712 (C=O str, s), 1629 (w), 1604 (w), 1519 (nitro str, s), 1494 (s), 1392 (w), 1354 (m), 1327 (nitro str, s), 1297 (m), 1281 (m), 1246 (m). ^1^H NMR (300 MHz, CDCl_3_) δ 10.81 (s, 1H), 7.97 (app. dt, ddd, *J*=6.9, 1.2, 1.1 Hz, 1H), 7.83 (s, 1H), 7.74 (app. dt, ddd, *J*=9.0, 1.2, 1.1 Hz, 1H), 7.68–7.62 (m, 2H), 7.61–7.45 (m, 5H), 7.31–7.27 (m, 2H), 7.22 (ddd, *J*=9.0, 6.8, 1.2 Hz, 1H), 7.03 (s, 1H), 6.75 (app. td, ddd, *J*=6.9, 6.8, 1.2 Hz, 1H), 4.29 (app. tt, dddd, *J*=12.4, 12.4, 4.2, 4.2 Hz, 1H), 3.56 (s, 2H), 3.05 (d, *J*=11.3 Hz, 2H), 2.39 (app. qd, dddd, *J*=12.1, 12.1, 12.1, 3.6 Hz, 2H), 2.15 (app. t, dd, *J*=11.6, 11.3 Hz, 2H), 1.79 (br d, *J*=11.9 Hz, 2H). ^13^C NMR (75 MHz, CDCl_3_) δ 155.3 (C=O), 145.0, 142.3, 141.7, 137.6, 133.2, 132.3, 130.9, 129.9, 129.7, 129.2, 129.1, 128.2, 128.0, 125.0, 123.5, 121.6, 121.2, 117.6, 112.5, 111.7, 107.1, 62.7, 53.0, 52.0, 29.5. HRMS (TOF MS ESI+): *m/z* calcd for C_32_H_28_ClN_6_O_3_^+^ [M+H]^+^, 579.1911, found 579.1926, error 2.6 ppm.

Compound **61** was prepared by a method similar to that for compound **56**, but afforded the product with the chlorine still present.

**6‐Amino‐5‐chloro‐1‐{1‐[4‐(3‐phenylimidazo[1,2‐*a*]pyridin‐2‐yl)benzyl]piperidin‐4‐yl}‐1,3‐dihydro‐2*H*‐benzo[*d*]imidazol‐2‐one (61)**. Off‐white solid (22 mg, 0.040 mmol, 43 %) (R_f_=0.30, 10 % MeOH/DCM). ^1^H NMR (300 MHz, CDCl_3_) δ 9.82 (s, 1H), 8.54 (s, C_Ar_−N*H*_2_), 7.93 (app. dt, ddd, *J*=6.9, 1.2, 1.1 Hz, 1H), 7.71 (app. dt, ddd, *J*=9.0, 1.1, 1.1 Hz, 1H), 7.69–7.65 (m, 2H), 7.62–7.40 (m, 5H), 7.34–7.26 (m, 2H), 7.21 (ddd, *J*=9.0, 6.7, 1.2 Hz, 1H), 6.89 (s, 1H), 6.88 (s, 1H), 6.74 (app. td, ddd, *J*=6.9, 6.7, 1.1 Hz, 1H), 4.50–4.29 (m, 1H), 3.73 (s, 2H), 3.20 (d, *J*=11.2 Hz, 2H), 2.64–2.44 (m, 2H), 2.40–2.22 (m, 2H), 1.78 (br d, *J*=12.1 Hz, 2H). HRMS (TOF MS ESI+): *m/z* calcd for C_32_H_30_ClN_6_O^+^ [M+H]^+^, 549.2170, found 549.2187, error 3.1 ppm.

Compound **12** was prepared by a method similar to that for compound **52**.

***N*** **‐(6‐Chloro‐2‐oxo‐3‐{1‐[4‐(3‐phenylimidazo[1,2‐*a*]pyridin‐2‐yl)benzyl]piperidin‐4‐yl}‐2,3‐dihydro‐1*H*‐benzo[*d*]imidazol‐5‐yl)acrylamide (12)**. Yellow solid (8 mg, 0.01 mmol, 68 %) (R_f_=0.33, 10 % MeOH/DCM). Mp 80–82 °C. IR (ATR, cm^−1^): 2920 (m), 2850 (m), 1729 (C=O str, s), 1694 (C=O str, m), 1622 (w), 1604 (w), 1514 (m), 1481 (m), 1447 (w), 1403 (m), 1366 (w), 1344 (C‐N str, s), 1259 (w), 1236 (w), 1203 (m). ^1^H NMR (300 MHz, CDCl_3_) δ 10.00 (s, 1H), 8.38 (s, 1H), 7.95 (app. dt, ddd, *J*=6.9, 1.2, 1.2 Hz, 1H), 7.74–7.68 (m, 1H), 7.72 (s, 1H), 7.64 (d, *J*=8.2 Hz, 2H), 7.60–7.42 (m, 5H), 7.30 (d, *J*=8.2 Hz, 2H), 7.20 (ddd, *J*=9.1, 6.8, 1.2 Hz, 1H), 7.07 (s, 1H), 6.73 (app. td, ddd, *J*=6.9, 6.8, 1.2 Hz, 1H), 6.47 (dd, *J*=16.8, 1.4 Hz, 1H), 6.31 (dd, *J*=16.8, 10.0 Hz, 1H), 5.82 (dd, *J*=10.0, 1.4 Hz, 1H), 4.23 (app. tt, dddd, *J*=12.2, 12.2, 3.8, 3.8 Hz, 1H), 3.56 (s, 2H), 3.05 (d, *J*=11.2 Hz, 2H), 2.62–2.44 (m, 2H), 2.23 –2.07 (m, 2H), 1.76 (br d, *J*=11.8 Hz, 2H). ^13^C NMR (75 MHz, CDCl_3_) δ 163.6 (C=O), 155.4 (C=O), 144.9, 142.4, 137.5, 133.1, 131.2, 130.9, 130.1, 129.7, 129.3, 129.1, 128.8, 128.3, 128.1, 125.2, 124.8, 123.4, 121.1, 117.6, 116.5, 112.4, 109.9, 103.9, 62.6, 53.1, 51.8, 29.1. HRMS (TOF MS ESI+): *m/z* calcd for C_35_H_32_ClN_6_O_2_^+^ [M+H]^+^, 603.2275, found 603.2292, error 2.8 ppm. Purity 99.5 %.

**2‐{4‐[(4‐Azidopiperidin‐1‐yl)methyl]phenyl}‐3‐phenyl‐1,6‐naphthyridin‐5(6*H*)‐one (63)**. Following a literature procedure,^[32]^ 4‐(5‐oxo‐3‐phenyl‐5,6‐dihydro‐1,6‐naphthyridin‐2‐yl)benzaldehyde **62** (0.260 g, 0.79 mmol), 4‐azidopiperidin‐1‐ium chloride **41** (1.5 equiv, 0.189 g, 1.16 mmol) and 95% formic acid (5.2 equiv, 0.20 mL, 0.19 g, 4.1 mmol) in MeCN (10 mL) were stirred at 80 °C for 45 h. The reaction was allowed to cool to RT, diluted with EtOAc (100 mL) and quenched with saturated aqueous NaHCO_3_ (50 mL). The layers were separated, and the aqueous layer was extracted with EtOAc (2×100 mL). The combined organic layers were dried over MgSO_4_, filtered and concentrated *in vacuo* to yield the crude material which was purified by column chromatography to afford the title compound **63** as a yellow solid. (0.026 g, 0.060 mmol, 8 %, 35 % brsm) (R_f_=0.12, 80 % EtOAc/hexane). **Mp** 196–198 °C. ^**1**^ **H NMR (300 MHz, CDCl_3_) δ** 11.25 (br d, *J*=21.1 Hz, 1H), 8.69 (s, 1H), 7.39 (d, *J*=8.2 Hz, 2H), 7.33–7.18 (m, 8H), 6.93 (d, *J*=7.4 Hz, 1H), 3.48 (s, 2H, C_Ar_−C*H*_2_−N), 3.47–3.30 (m, 1H, C*H*−N_3_), 2.78–2.67 (m, 2H), 2.21–2.07 (m, 2H), 1.93–1.83 (m, 2H), 1.73–1.57 (m, 2H). **HRMS (TOF MS ESI+)**: *m/z* calcd for C_26_H_25_N_6_O^+^ [M+H]^+^, 437.2090, found 437.2093, error 0.7 ppm.

Compound **13** was prepared by a method similar to that for compounds **3** and **4**.

***N*** **‐[3‐(1‐{1‐[4‐(5‐Oxo‐3‐phenyl‐5,6‐dihydro‐1,6‐naphthyridin‐2‐yl)benzyl]piperidin‐4‐yl}‐1*H*‐1,2,3‐triazol‐4‐yl)phenyl]acrylamide (13)**. Yellow solid (14 mg, 0.023 mmol, 40 %) (R_f_=0.05, EtOAc). Mp 252–254 °C. IR (ATR, cm^−1^): 3405 (N−H str, m), 3230 (m), 2921 (m), 1652 (C=O str, s), 1629 (C=O str, s), 1589 (s), 1567 (m), 1532 (m), 1470 (w), 1445 (m), 1398 (s), 1341 (w), 1272 (w), 1239 (w), 1209 (m). ^1^H NMR (300 MHz, CDCl_3_&MeOD‐d_4_) δ 8.58 (s, 1H), 7.92 (s, 1H), 7.83 (app. dt, ddd, *J*=8.1, 1.7, 1.5 Hz, 1H), 7.79 (app. t, dd, *J*=1.7, 1.7 Hz, 1H), 7.38 (app. dt, ddd, *J*=7.7, 1.7, 1.5 Hz, 1H), 7.41–7.24 (m, 4H), 7.26–7.15 (m, 5H), 7.17–7.11 (m, 2H), 6.83 (d, *J*=7.4 Hz, 1H), 6.34 (d, *J*=3.1 Hz, 1H), 6.32 (d, *J*=8.6 Hz, 1H), 5.69 (dd, *J*=8.6, 3.1 Hz, 1H), 4.43–4.37 (m, 1H), 3.51 (s, 2H), 2.96 (d, *J*=10.6 Hz, 2H), 2.23–1.99 (m, 6H). ^13^C NMR (75 MHz, CDCl_3_&MeOD‐d_4_) δ 164.7 (C=O), 163.6 (C=O), 162.5, 153.2, 147.2, 139.1, 138.8, 138.6, 138.2, 137.8, 135.1, 131.7, 131.2, 130.6, 130.0, 129.9, 129.6, 128.9, 128.4, 127.6, 127.5, 121.3, 120.8, 120.0, 118.3, 116.6, 107.9, 62.3, 58.5, 52.0, 32.3. HRMS (TOF MS ESI+): *m/z* calcd for C_37_H_34_N_7_O_2_^+^ [M+H]^+^, 608.2774, found 608.2778, error 0.7 ppm. Purity 88.8 %.

## Supporting Information

Protocols for evaluation, additional results from the evaluation, NMR spectra, 2D NMR spectroscopic assignments, and LC‐MS traces for purity are available in the Supporting Information.

## Author Contributions

The manuscript was written through contributions of all authors. Contributions to the work was done as follows: L.v.d.W. (Synthesis and writing of the manuscript), J.W. (HTRF and biochemistry), A.T. (Original synthesis of precursor **28**), I.L. (CTG, X‐ray crystallography), L.Q. (HTRF), M.L. (Synthesis of precursor **62**), N.U. (Synthesis of precursor **62**), M.P.M. (X‐ray crystallography), I.R.G. (Synthesis support), S.C.P. (Molecular modelling), D.R. (Group leader, Dortmund) and W.A.L.v.O. (Group leader, Stellenbosch).

## Abbreviations

Akt1^E17K/E49K^, Akt1 with glutamic acid to lysine substitutions at amino acids 17 and 49; CTG, CellTiter‐Glo® Luminescent Cell Viability Assay; CuAAC, copper(I)‐catalyzed azide alkyne cycloaddition; EC_50_, half‐maximal effective concentration; HTRF®, homogeneous time‐resolved fluorescence; IC_50_, half‐maximal inhibitory concentration; *K*_I_, inhibition constant; *k_inact_*, rate of enzyme inactivation; mTOR, mammalian target of rapamycin; PH, pleckstrin homology; PTEN, phosphatase and tensin homologue deleted on chromosome 10; TOCSY, total correlation spectroscopy.

## Conflict of interest

The authors declare no conflict of interest.

1

## Supporting information

As a service to our authors and readers, this journal provides supporting information supplied by the authors. Such materials are peer reviewed and may be re‐organized for online delivery, but are not copy‐edited or typeset. Technical support issues arising from supporting information (other than missing files) should be addressed to the authors.

Supporting Information

## Data Availability

The data that support the findings of this study are available in the supplementary material of this article.
