# Supplementary material for: Covalent Allosteric Inhibitors of Akt Generated Using a Click Fragment Approach
Source: ChemMedChem. 2022 Mar 9;17(10):e202100776. doi: 10.1002/cmdc.202100776 (PMC9311865; doi:10.1002/cmdc.202100776)
Supplement: Supplementary file 1 — Supporting Information [file CMDC-17-0-s001.pdf]

ChemMedChem

Supporting Information

Covalent Allosteric Inhibitors of Akt Generated Using a Click Fragment Approach

Leandi van der Westhuizen, Jörn Weisner, Abu Taher, Ina Landel, Lena Quambusch, Marius Lindemann, Niklas Uhlenbrock, Matthias P. Müller, Ivan R. Green, Stephen C. Pelly, Daniel Rauh,* and Willem A. L. van Otterlo*

Supporting Information

Table of Contents

1. Evaluation 2
2. Evaluation results 4
3. Characterisation 7
4. Molecular Modelling 57
5. References 58

1. Evaluation

IDENTIFICATION OF COVALENT BINDERS OF AKT

In order to identify covalent binders of Akt, each compound was co-incubated with wild-type Akt1 and then analysed by LC-MS. A mass increase equivalent to the mass of the compound indicated covalent binding.

For analysis of compound **3**, the wild-type Akt1 used was prepared as described by Fang *et al.* and the protocol followed was as described by Weisner *et al.*[1,2] A similar protocol was followed for the remainder of the compounds, as described by Uhlenbrock *et al.*, with the wild-type Akt1 used prepared as described in the same article.[3]

The results are given in Figure S1.

BIOCHEMICAL ASSAYS

The HTRF® KinEASE™ assay (Cisbio) was used to determine the IC_{50} values of the synthesised target compounds.[4] This assay was done as described by Weisner *et al*. (Akt1) and Quambusch *et al.* (Akt2 and Akt3).[1,5] The three wild-type Akt isoforms used were acquired from ProQinase.

Using a protocol published by Krippendorff *et al.*, K_I, k_{inact} and k_{inact}/K_I could be directly estimated from time-dependent IC_{50} determinations by kinetic analysis with HTRF® KinEASE™, as described by Weisner *et al.*[1,6] The same wild-type Akt1 was used as for the initial HTRF® KinEASE™ assay.

The results are given in Tables 1 and 2.

STRUCTURAL BIOLOGY

For co-crystallisation, the protein expression and purification as well as the crystallisation experiments and data processing were performed as described by Weisner *et al.*[7] Due to insufficient data quality the crystal structure was not submitted to the PDB.

CELLULAR ASSAYS

The CellTiter-Glo® Luminescent Cell Viability Assay from Promega was used for the cellular assays, performed as explained by Weisner *et al.*[7,8] Information on the cancer cell lines are given in Table S1, the control compounds used in Figure S3 and the results in Table S2.

Table S1. The cell lines used for the cellular assays, including what cancer type they are predominantly found in, and where the cell lines were acquired form.

<table>
<tr><th>Cell line</th><th>Cancer type</th><th>Acquired from</th></tr>
<tr><td>AN3CA</td><td>Endometrium</td><td>ATCC (provided by Prof. Jan G. Hengstler at Leibniz-Institut für Arbeitsforschung Dortmund)</td></tr>
<tr><td>KU-19-19</td><td>Bladder</td><td>Deutsche Sammlung von Mikroorganismen und Zellkulturen (DSMZ)</td></tr>
<tr><td>BT-474</td><td rowspan="4">Breast</td><td rowspan="2">CLS Cell Lines Service (provided by Prof. Jan G. Hengstler at Leibniz-Institut für Arbeitsforschung Dortmund)</td></tr>
<tr><td>MCF-7</td></tr>
<tr><td>T47D</td><td rowspan="2">Sigma-Aldrich/ECACC</td></tr>
<tr><td>ZR-75-1</td></tr>
</table>

2. Evaluation results

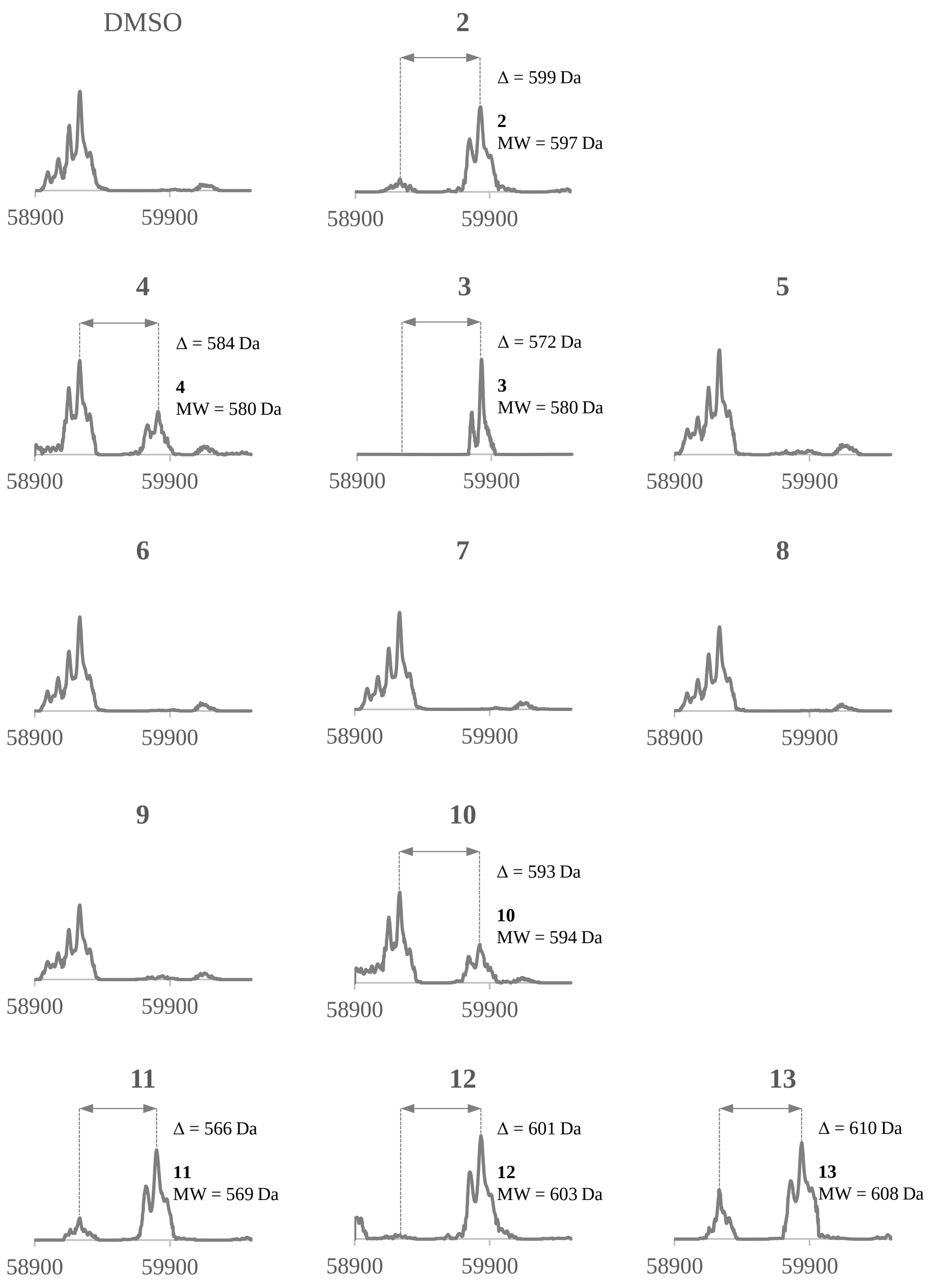

Figure S1. The LC-MS traces of the compounds to identify covalent modifiers of wild-type Akt1. The negative control was Akt1 incubated in dimethyl sulfoxide, with no potential ligand added, and the positive control was Akt1 incubated with **2**. Published data for compound **3** is included.[2]

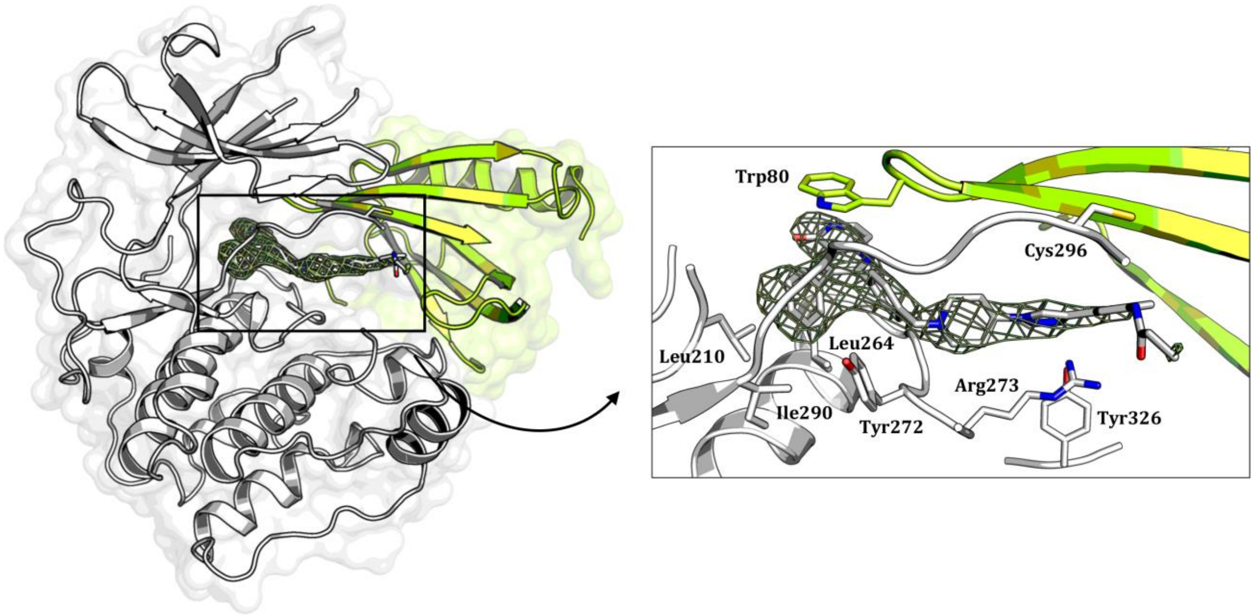

Figure S2. Co-crystal structure of Akt1 (2-446) in complex with covalent-allosteric inhibitor **13** indicates the predicted binding mode in the allosteric pocket between the catalytic kinase (gray) and the regulatory PH domain (green). The F_O-F_C simulated annealing omit map ($\sigma = 2.5$) is shown.

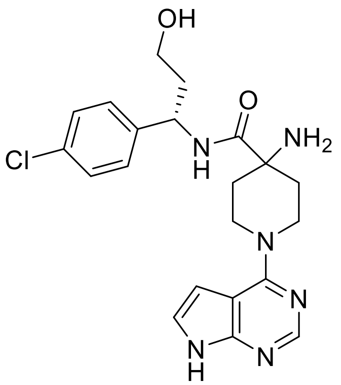

Capivasertib
AZD5363

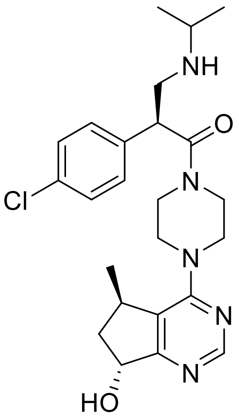

Ipatasertib
GDC-0068

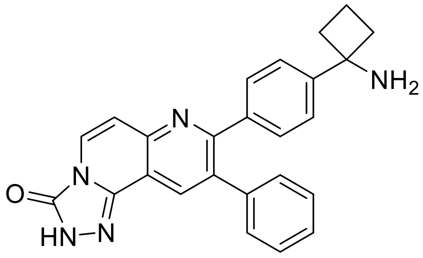

MK-2206

Figure S3. The control compounds used for the cellular data. Borussertib **2** is shown in Figure 1.

3. Characterisation

NMR SPECTRA

4-(6-Methylimidazo[1,2-*a*]pyridin-2-yl)benzonitrile (18).

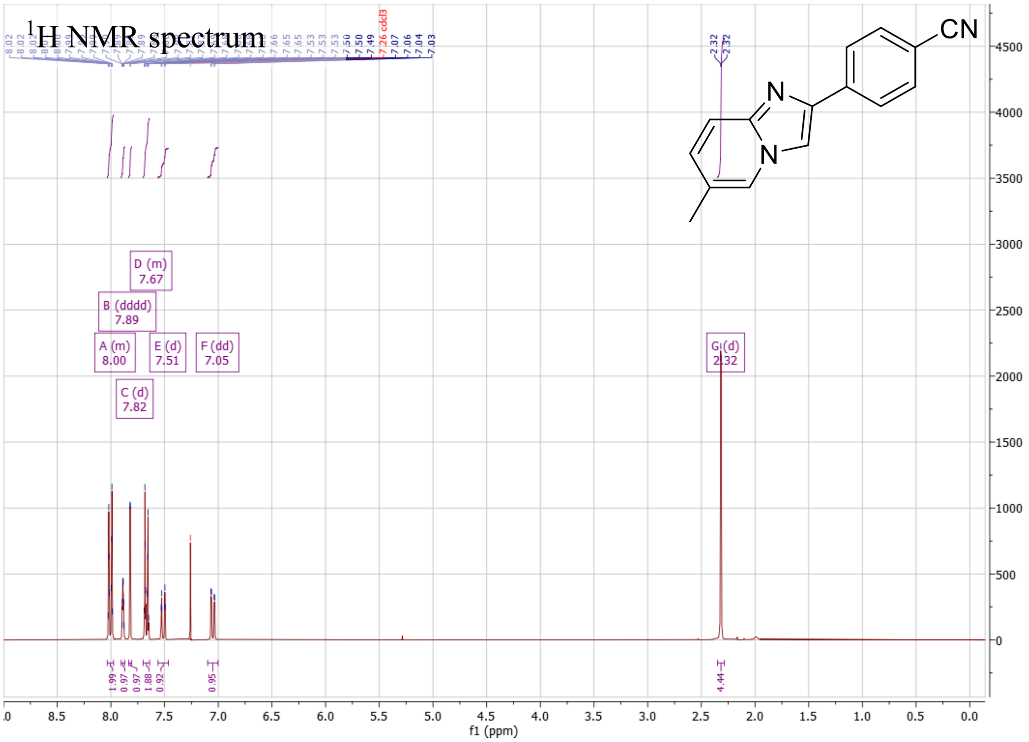

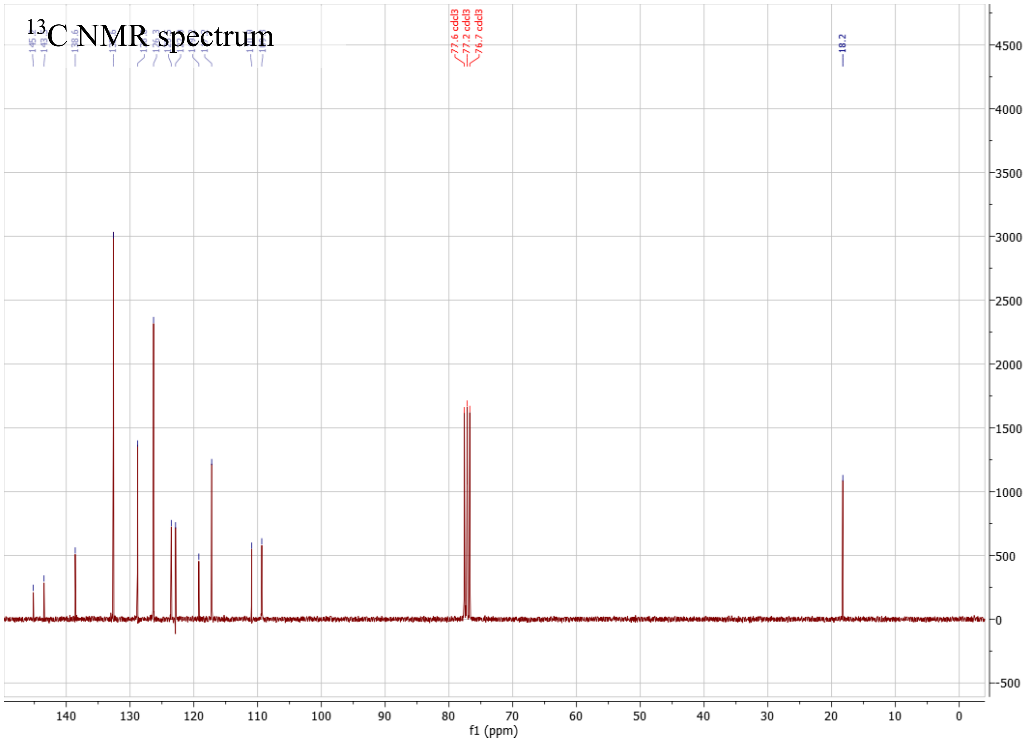

4-(3-Bromo-6-methylimidazo[1,2-*a*]pyridin-2-yl)benzonitrile (20).

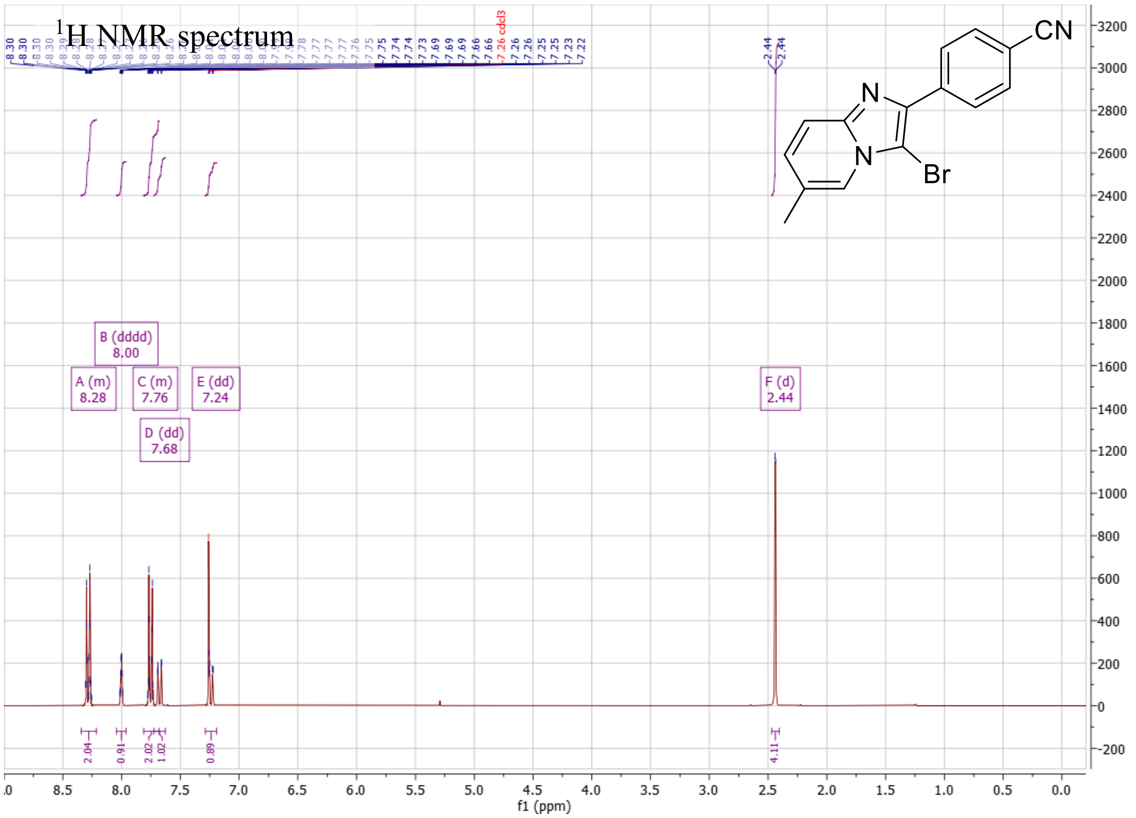

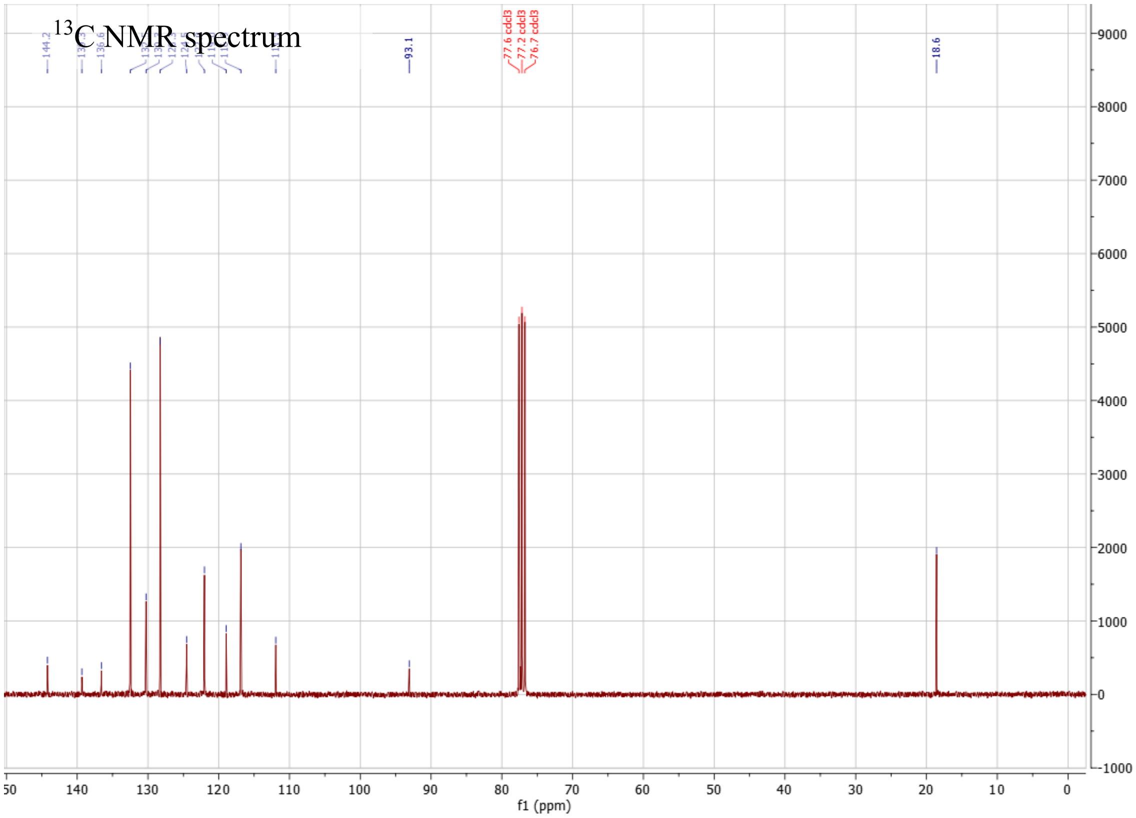

4-[3-(*o*-Tolyl)imidazo[1,2-*a*]pyridin-2-yl]benzonitrile (25).

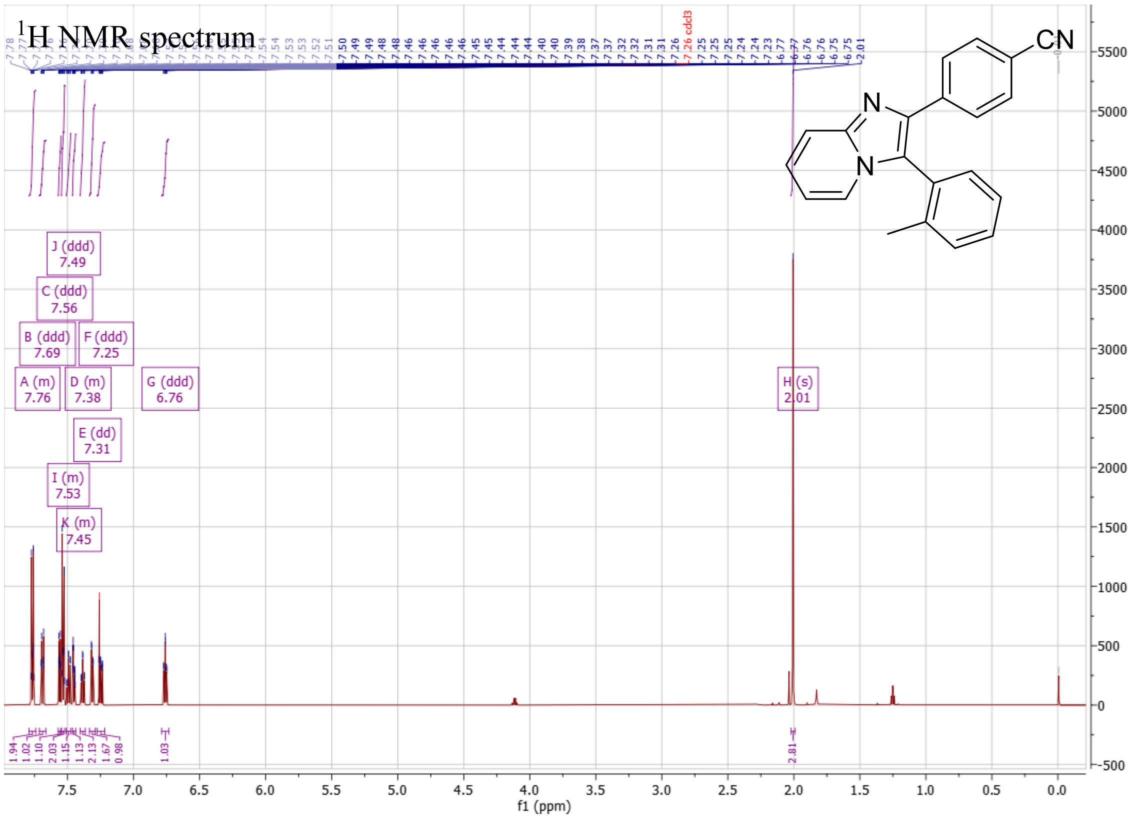

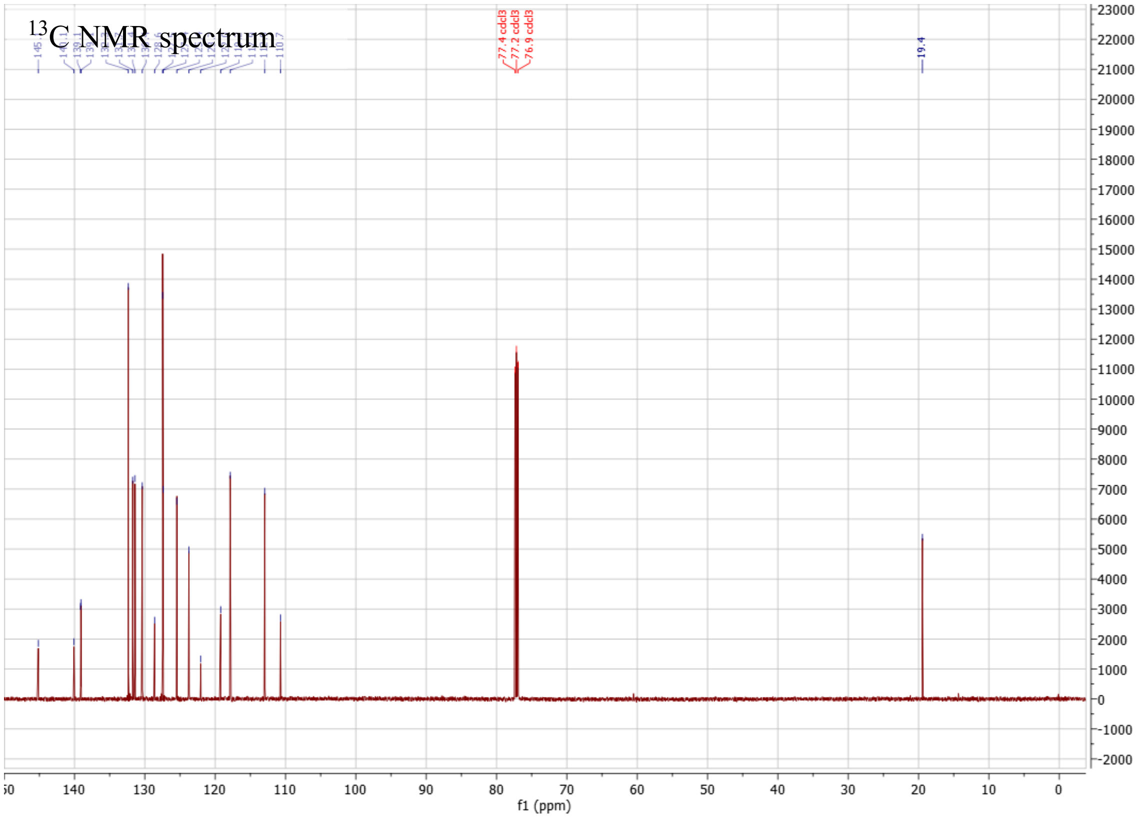

4-[3-(*m*-Tolyl)imidazo[1,2-*a*]pyridin-2-yl]benzonitrile (26).

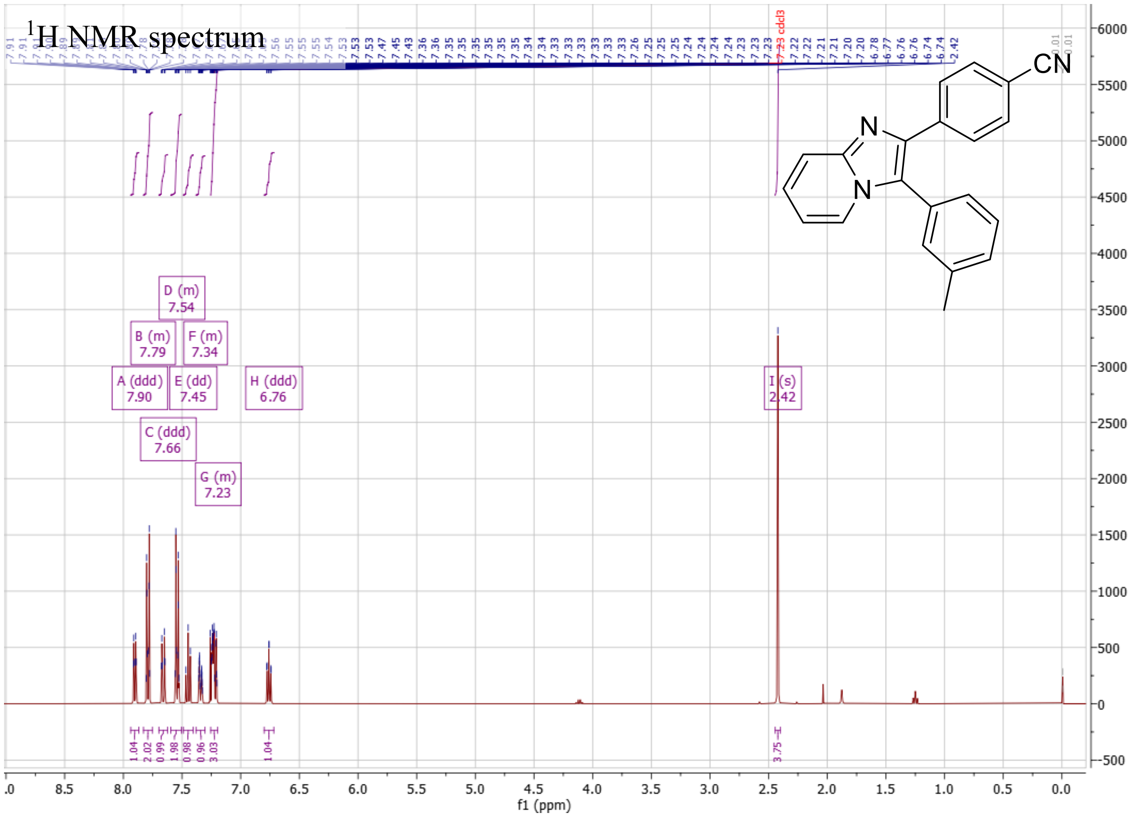

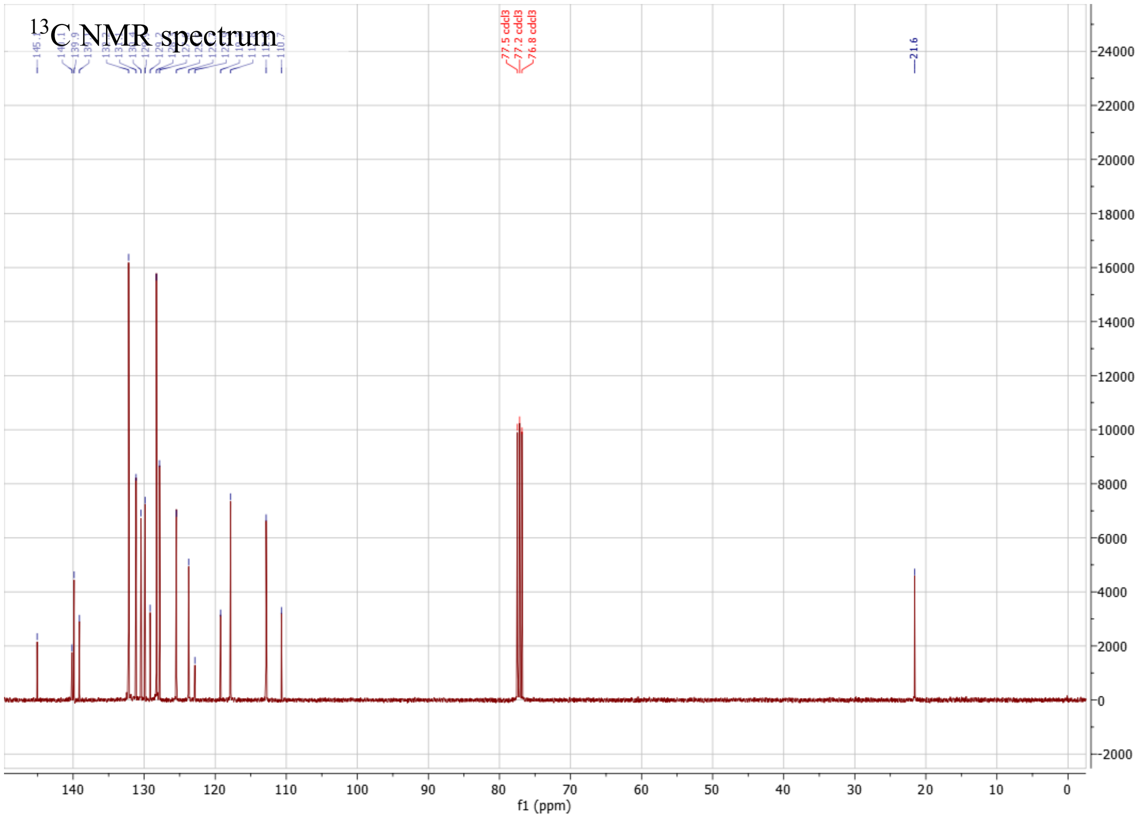

4-(6-Methyl-3-phenylimidazo[1,2-*a*]pyridin-2-yl)benzonitrile (27).

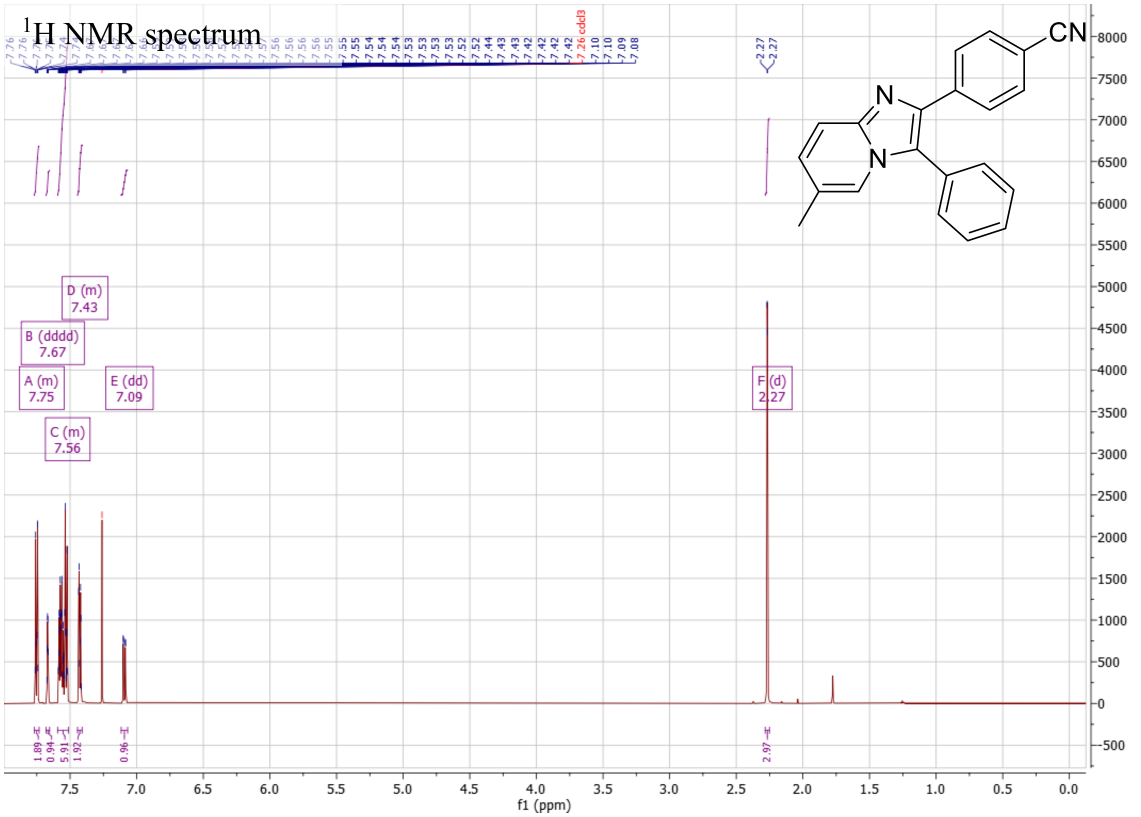

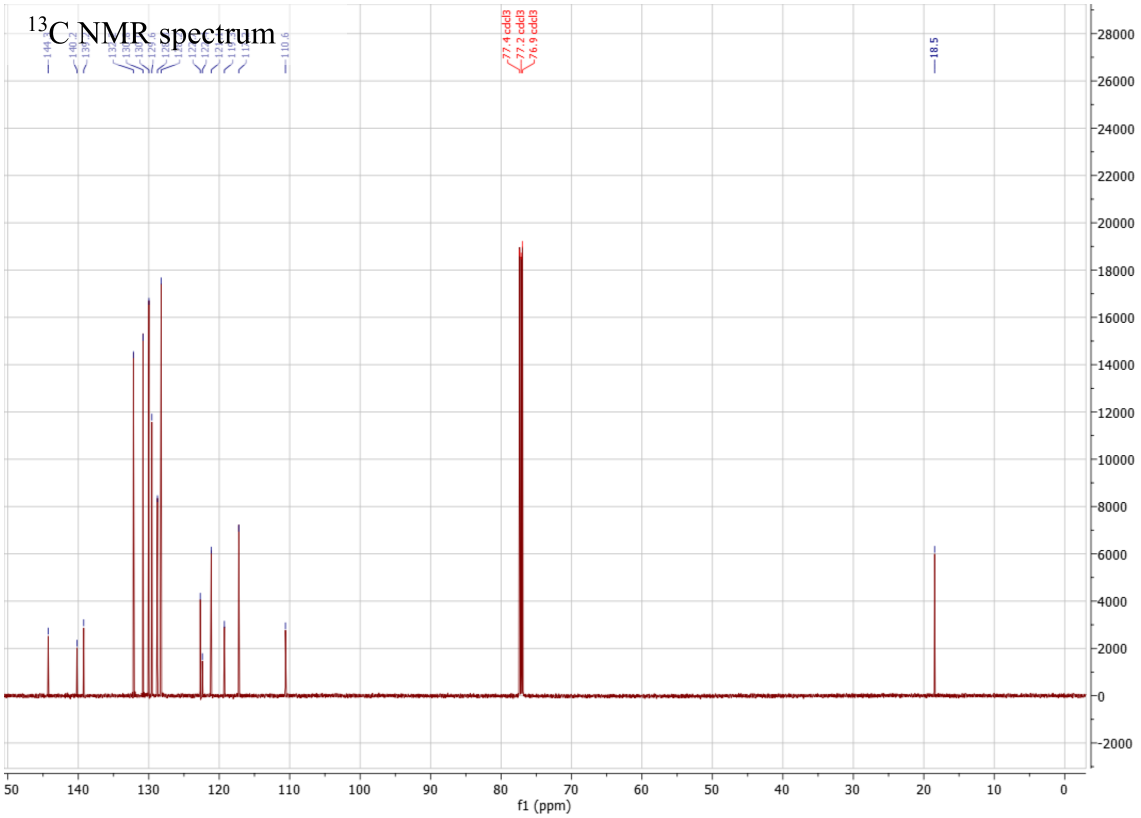

4-[3-(*o*-Tolyl)imidazo[1,2-*a*]pyridin-2-yl]benzaldehyde (29).

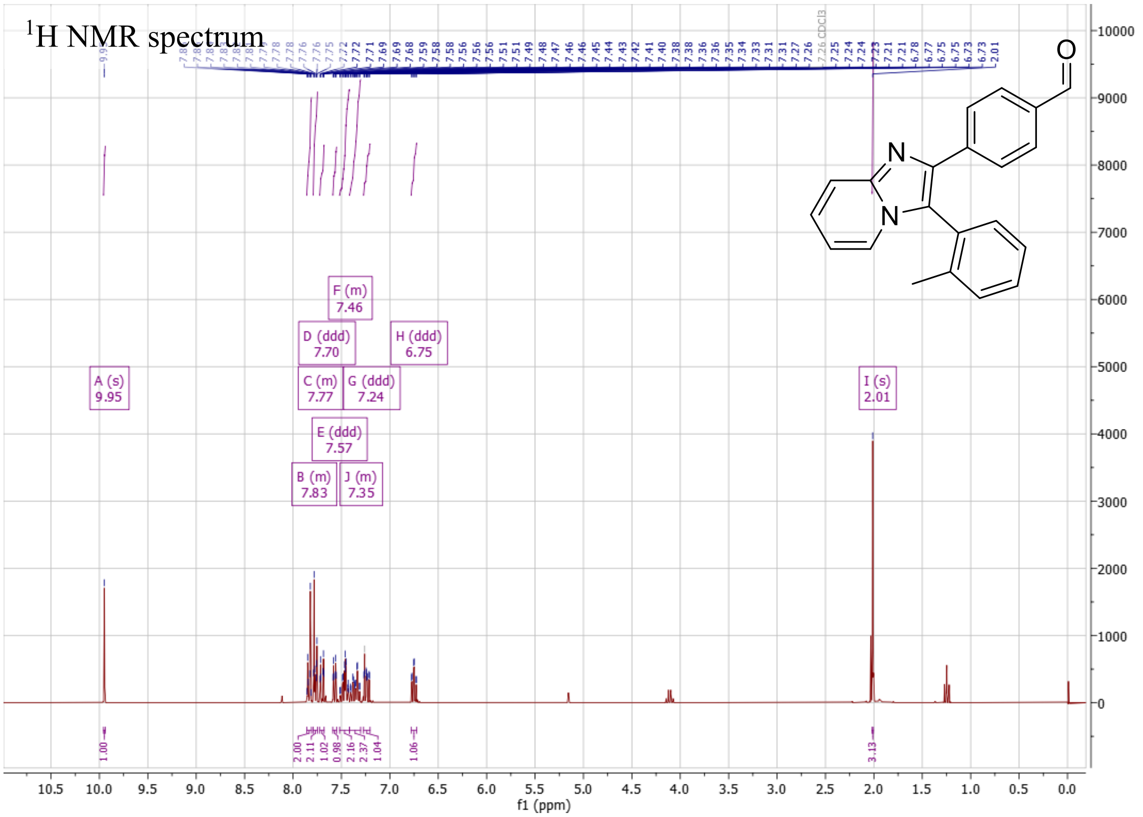

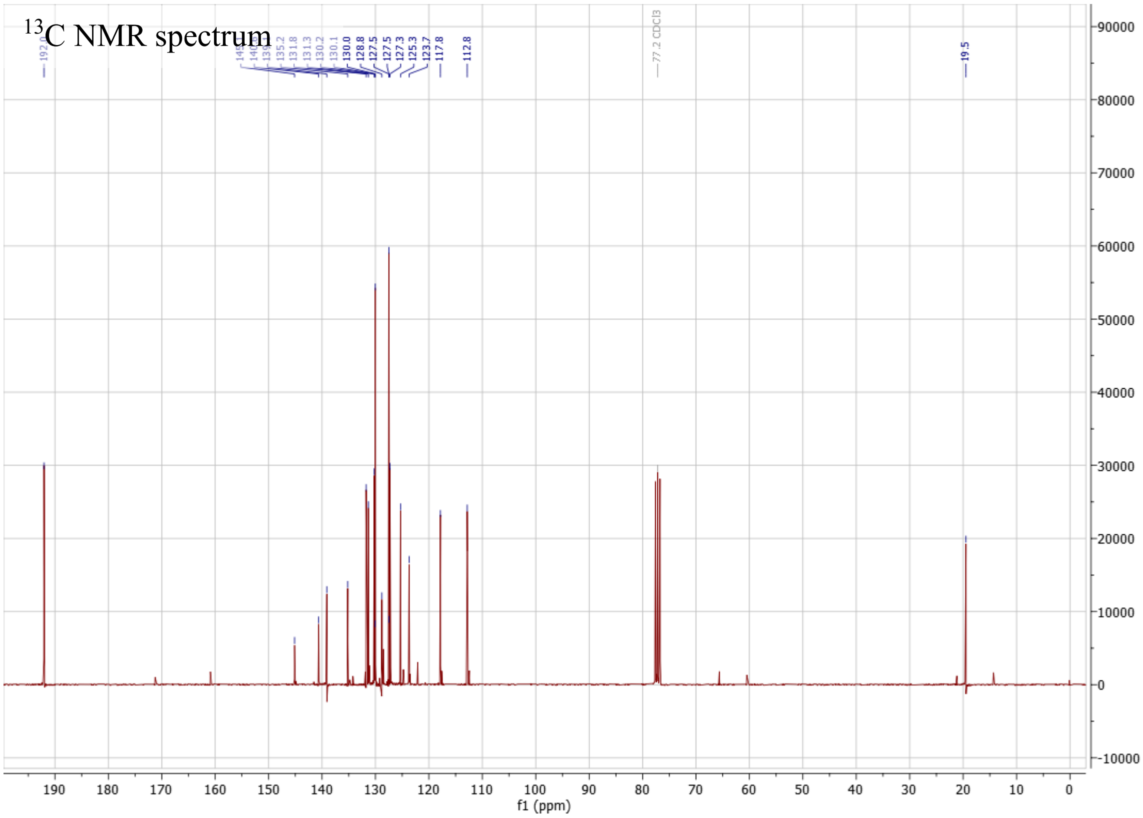

4-[3-(*m*-Tolyl)imidazo[1,2-*a*]pyridin-2-yl]benzaldehyde (30).

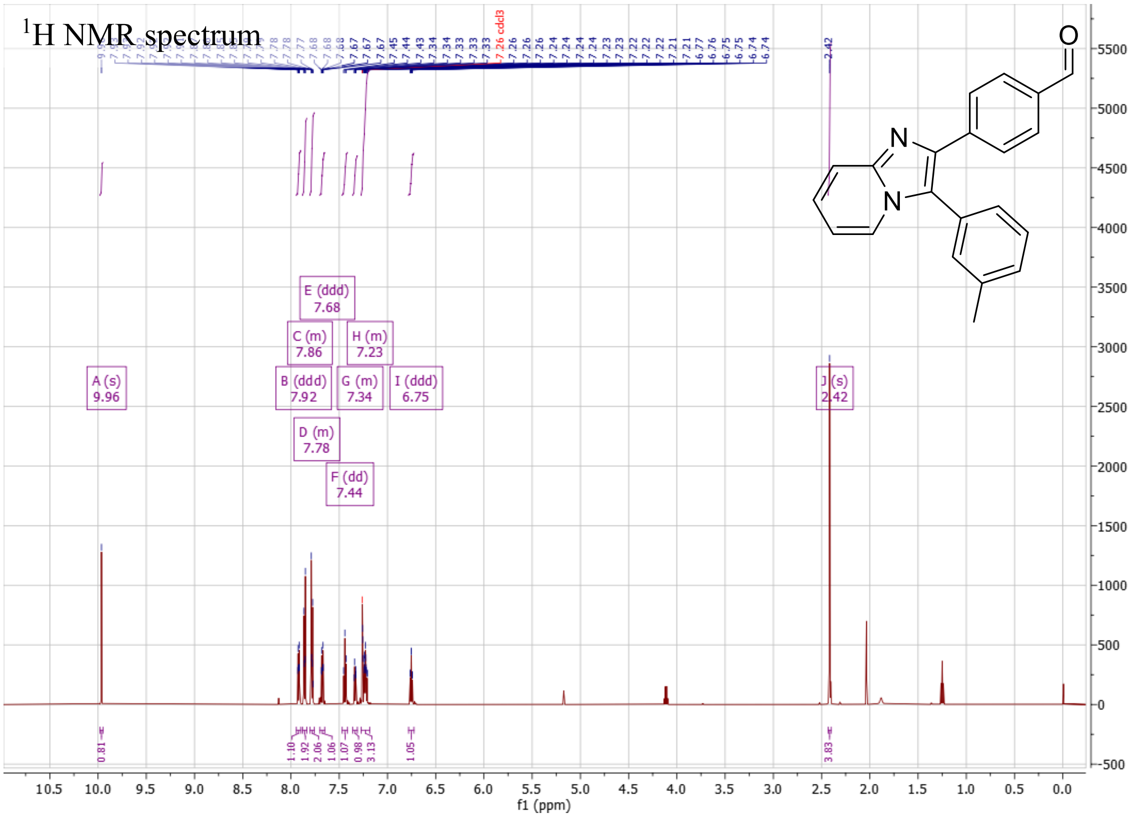

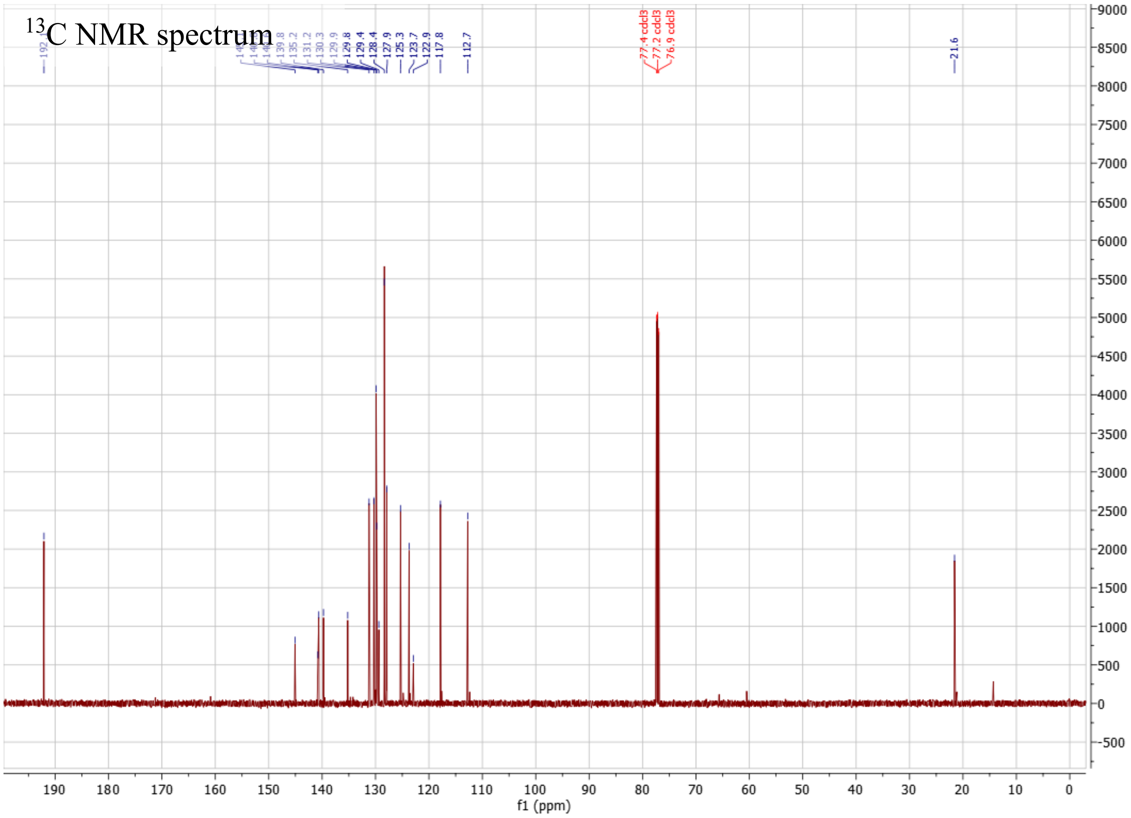

4-(6-Methyl-3-phenylimidazo[1,2-*a*]pyridin-2-yl)benzaldehyde (31).

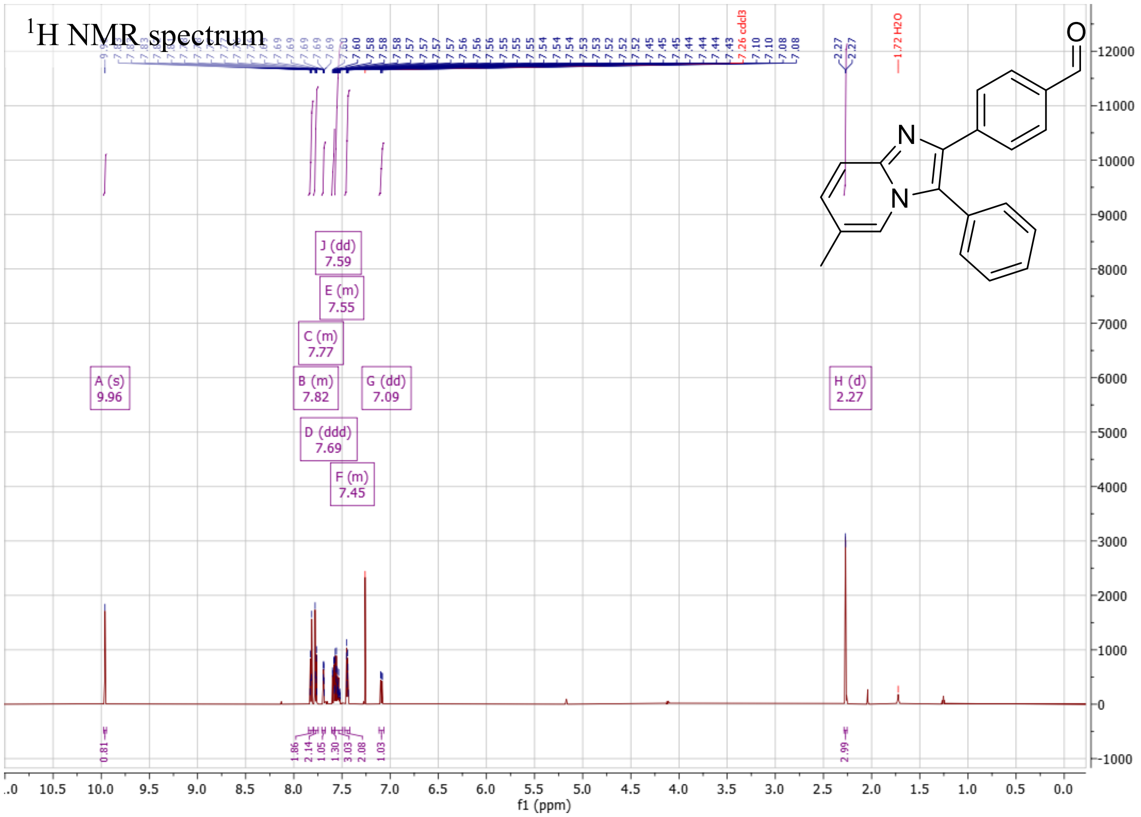

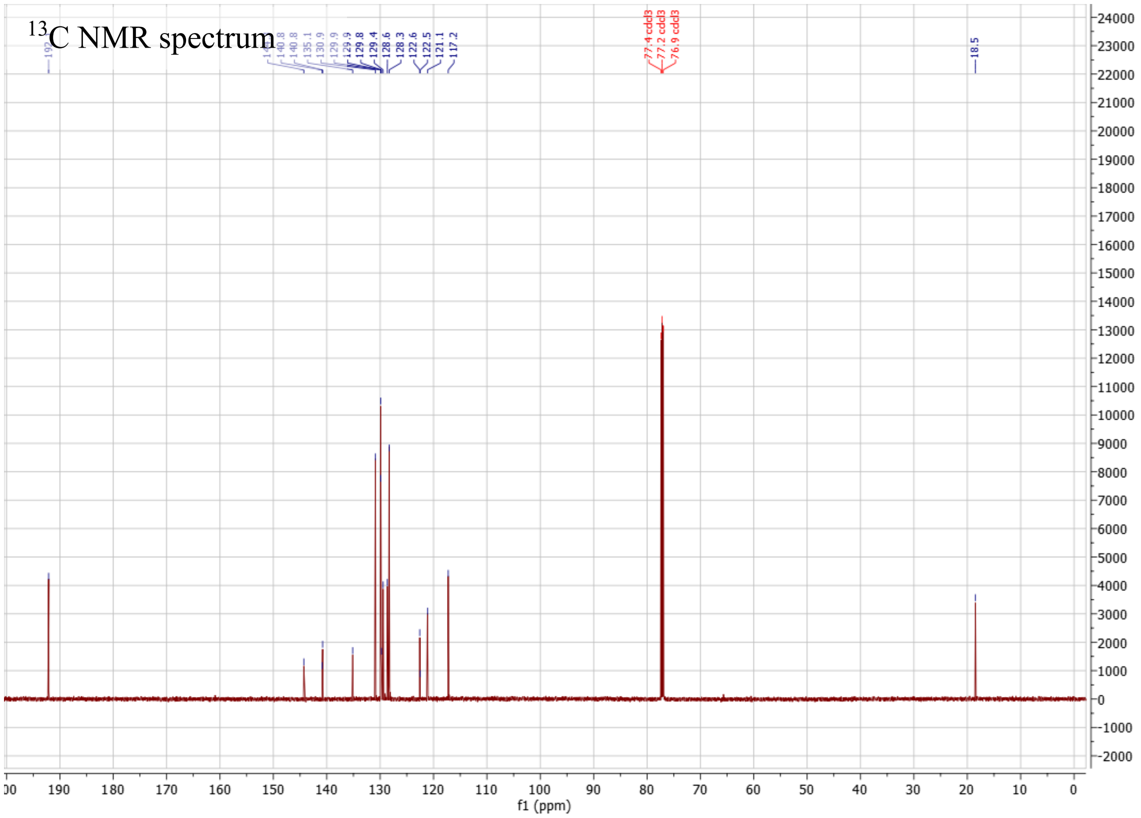

{4-[3-(*o*-Tolyl)imidazo[1,2-*a*]pyridin-2-yl]phenyl}methanol (33).

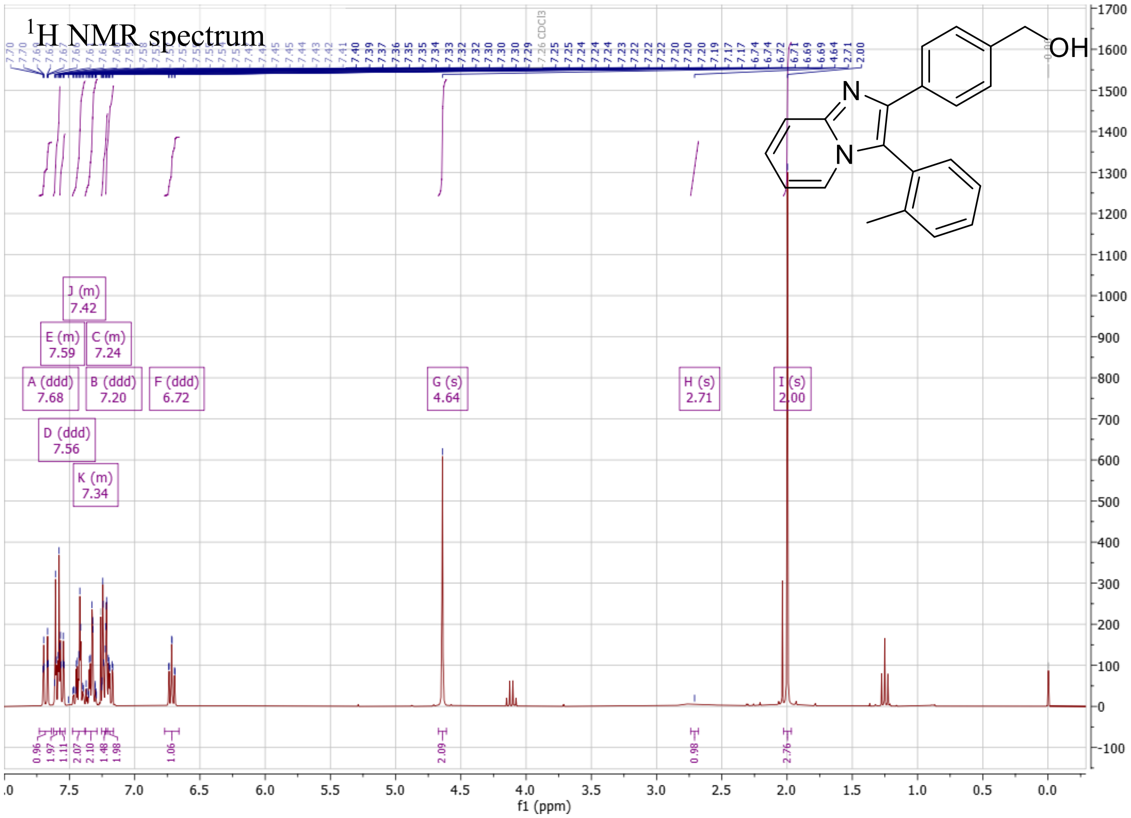

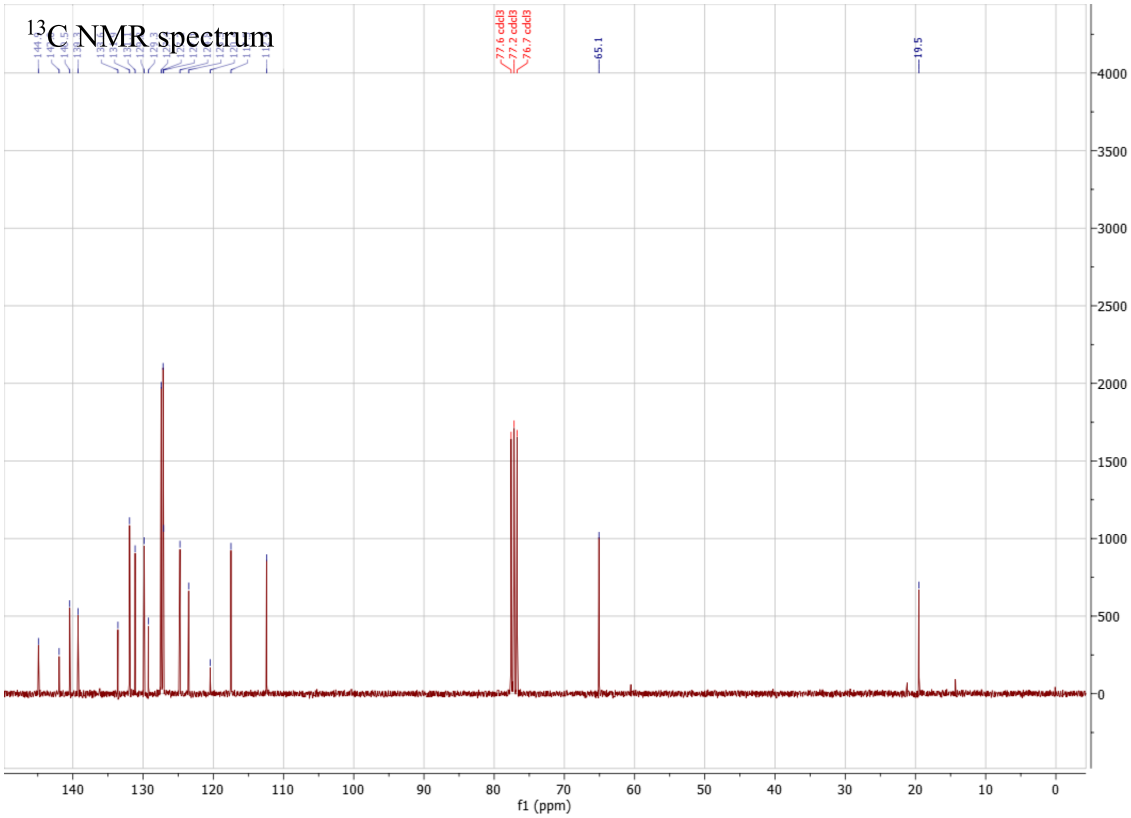

{4-[3-(*m*-Tolyl)imidazo[1,2-*a*]pyridin-2-yl]phenyl}methanol (34).

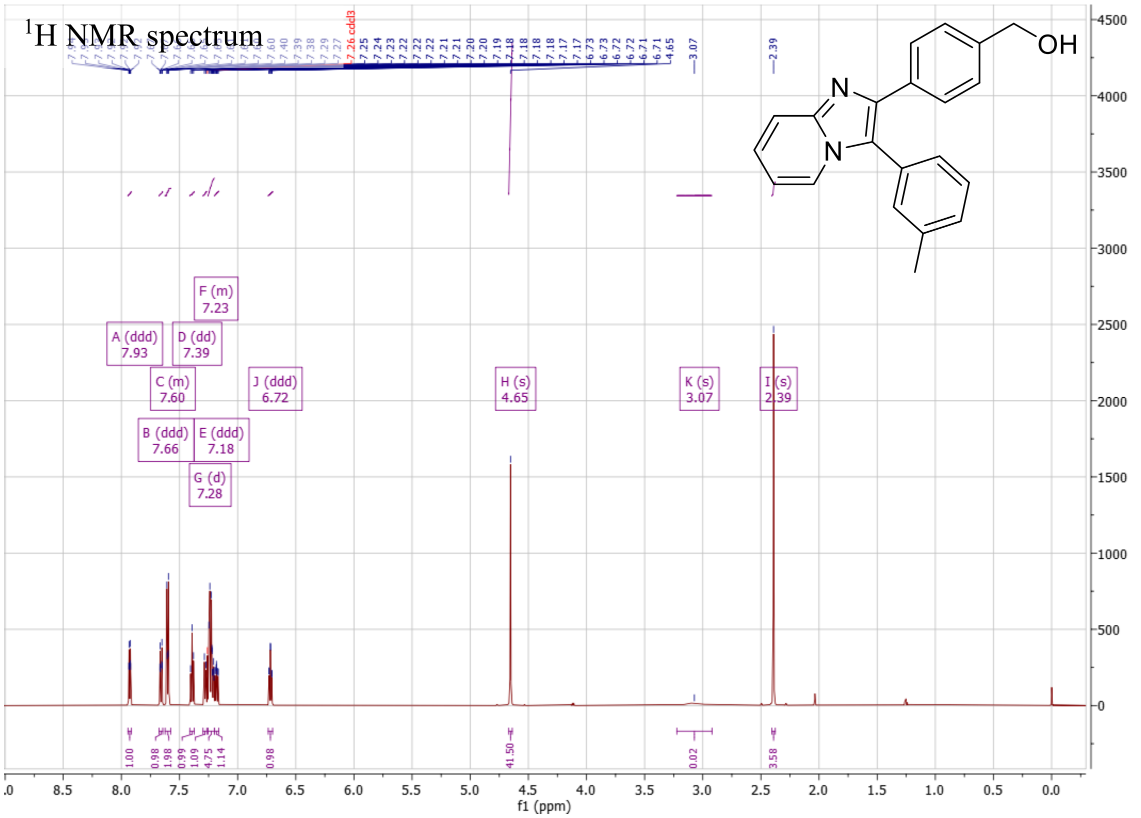

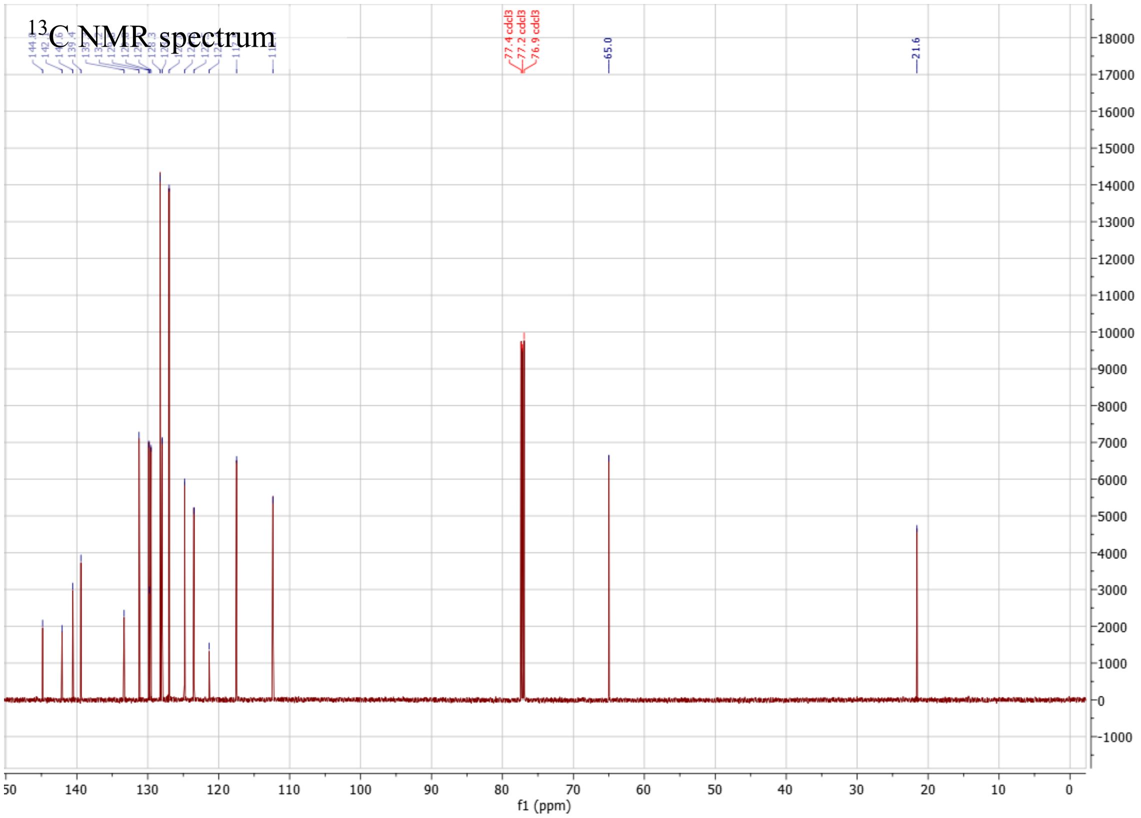

[4-(6-Methyl-3-phenylimidazo[1,2-*a*]pyridin-2-yl)phenyl]methanol (35).

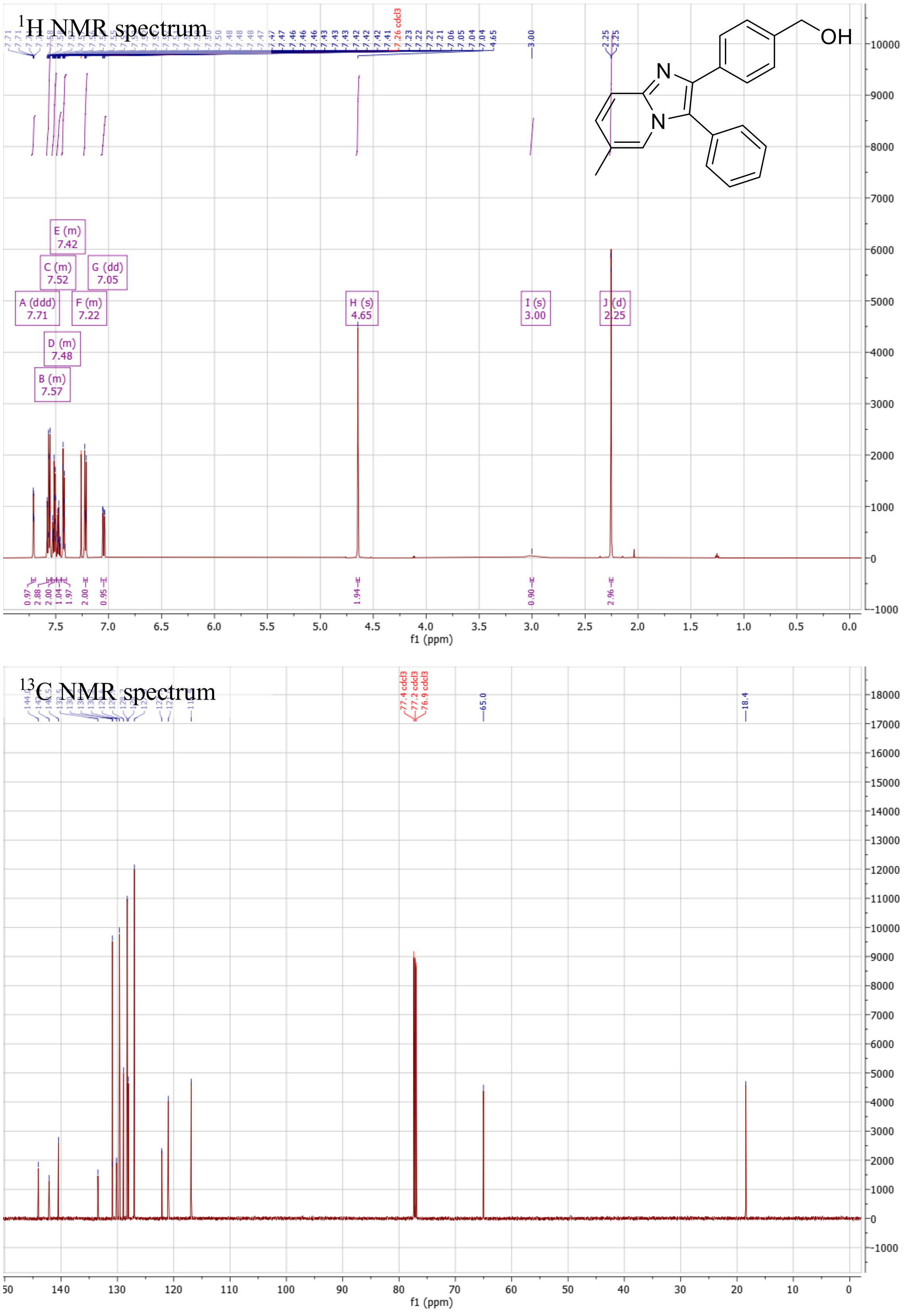

2-[4-(Chloromethyl)phenyl]-3-phenylimidazo[1,2-*a*]pyridine (36).

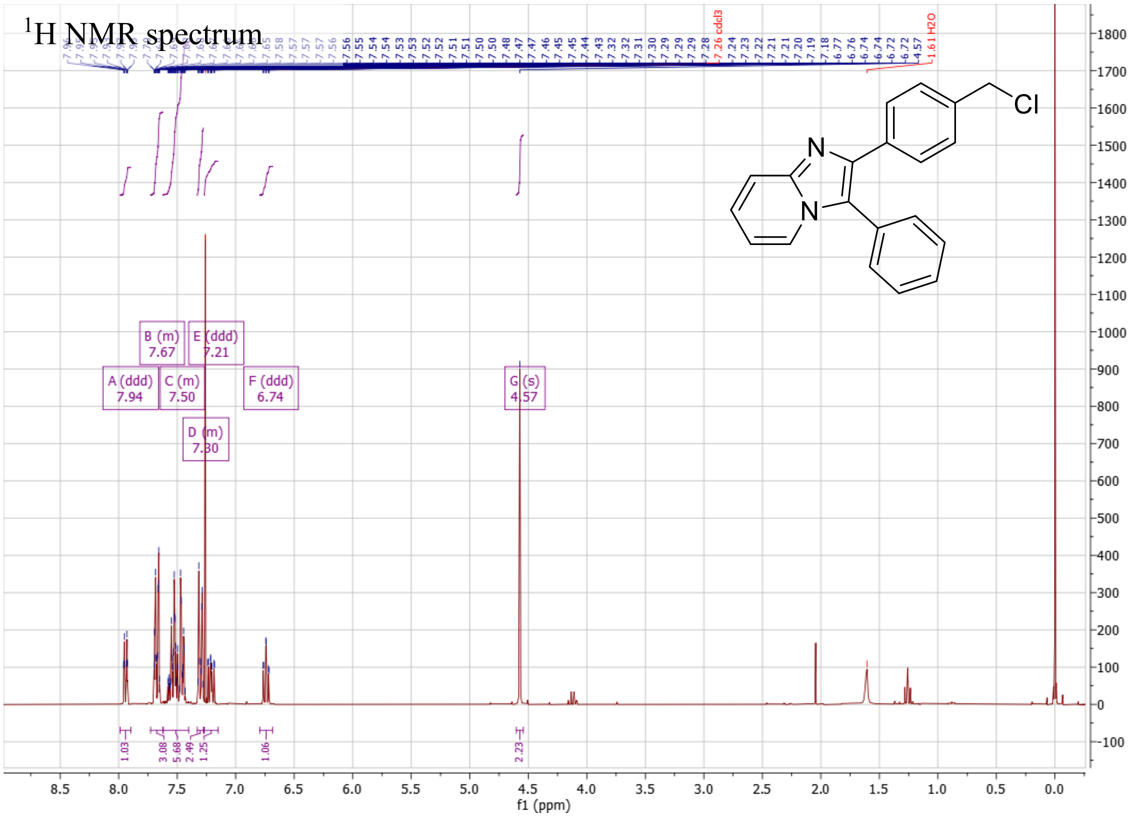

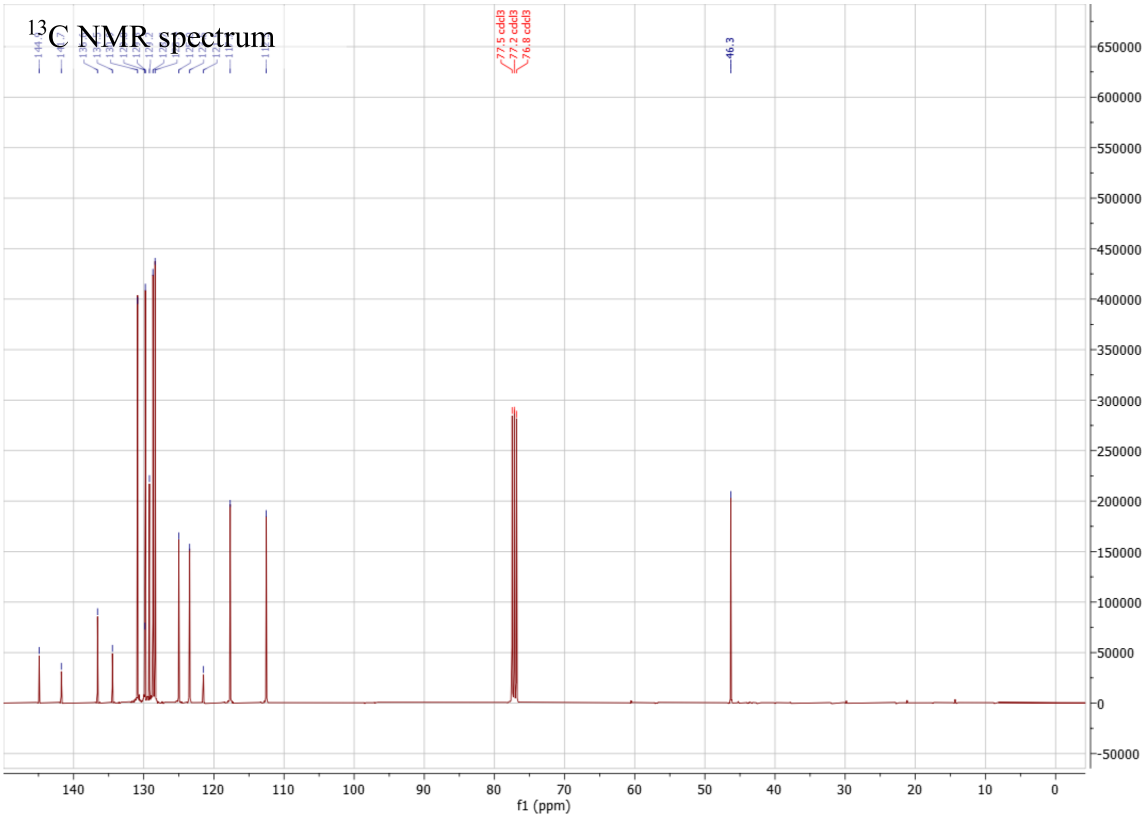

2-[4-(Chloromethyl)phenyl]-3-(*o*-tolyl)imidazo[1,2-*a*]pyridine (37).

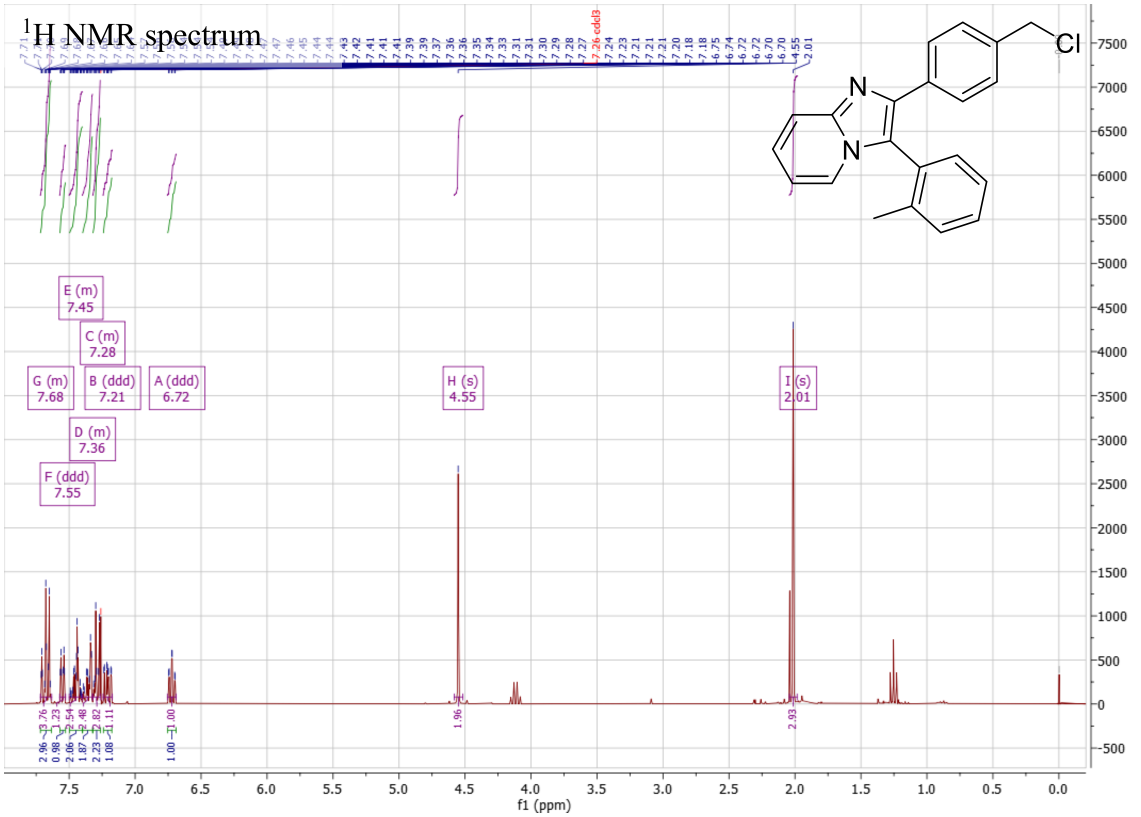

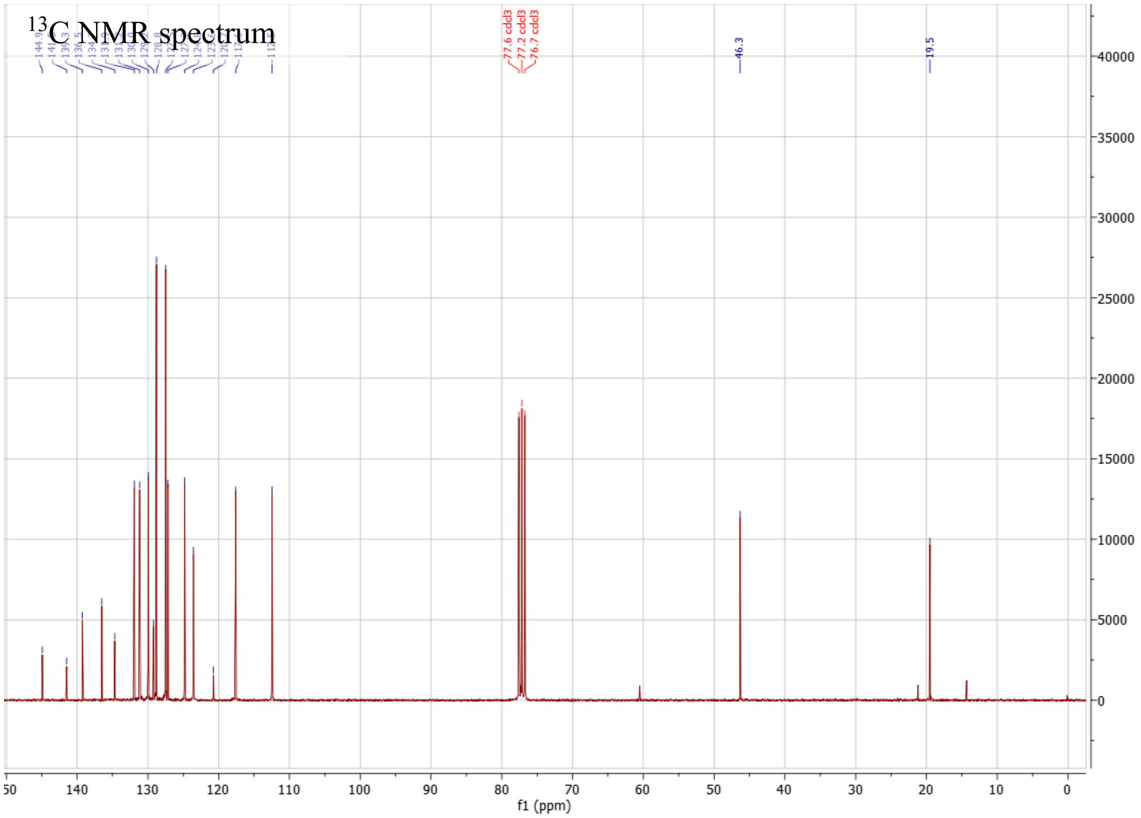

2-[4-(Chloromethyl)phenyl]-3-(*m*-tolyl)imidazo[1,2-*a*]pyridine (38).

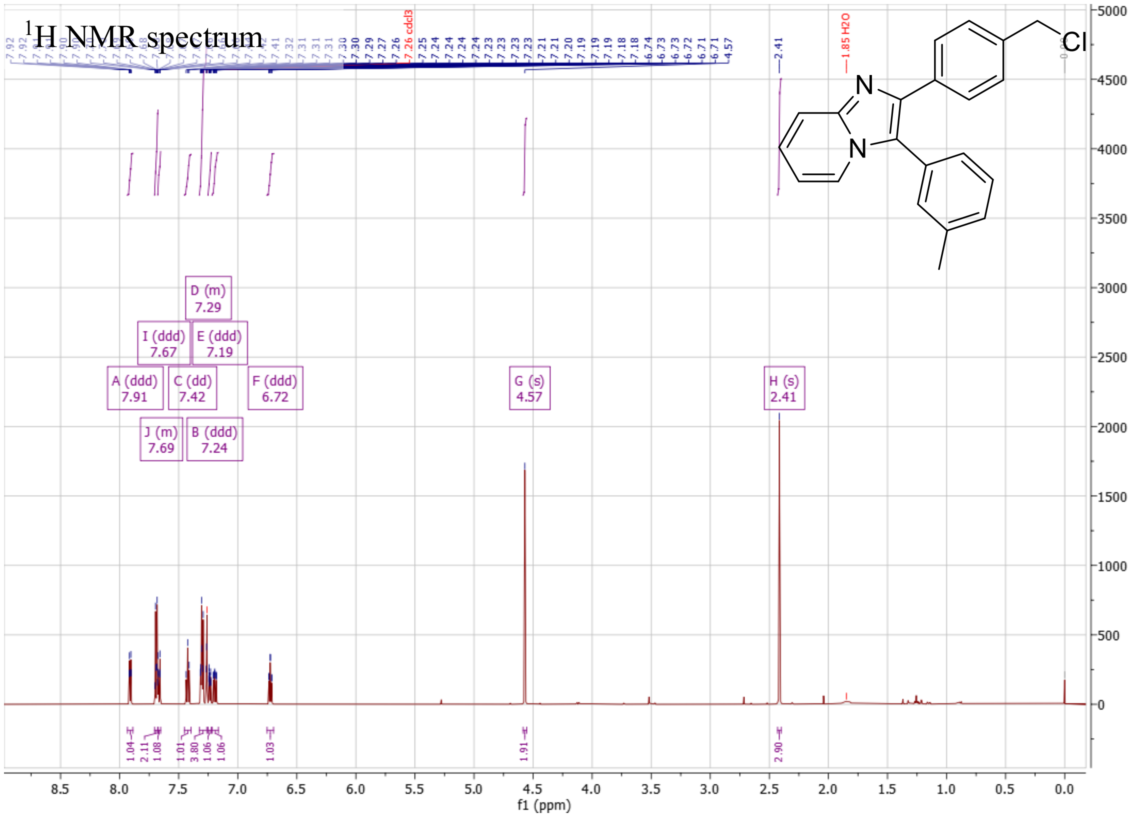

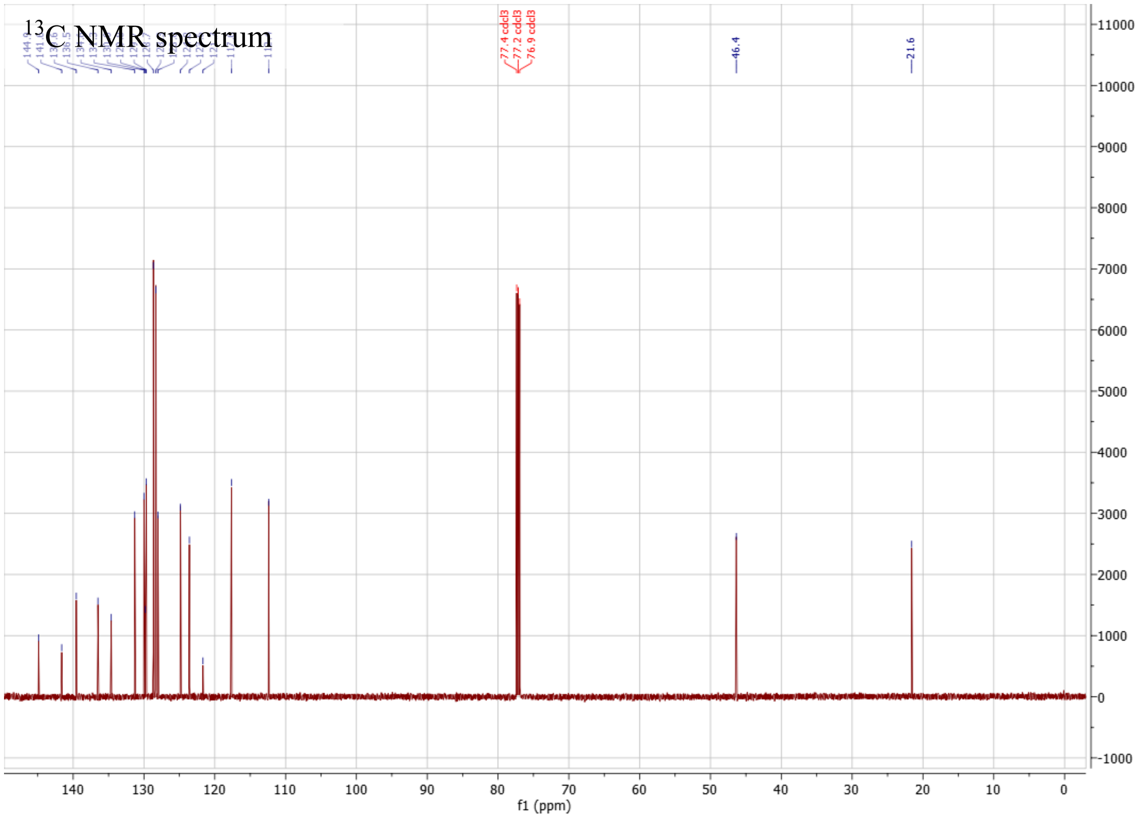

2-[4-(Chloromethyl)phenyl]-6-methyl-3-phenylimidazo[1,2-*a*]pyridine (39).

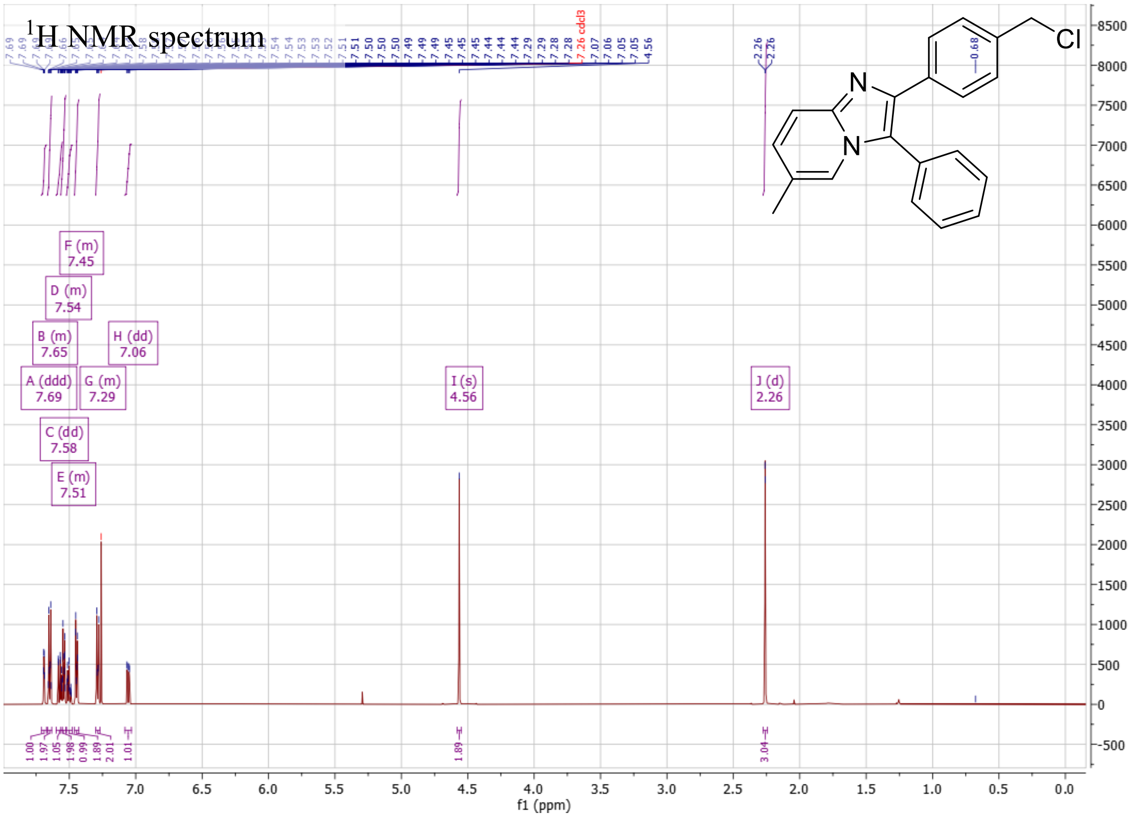

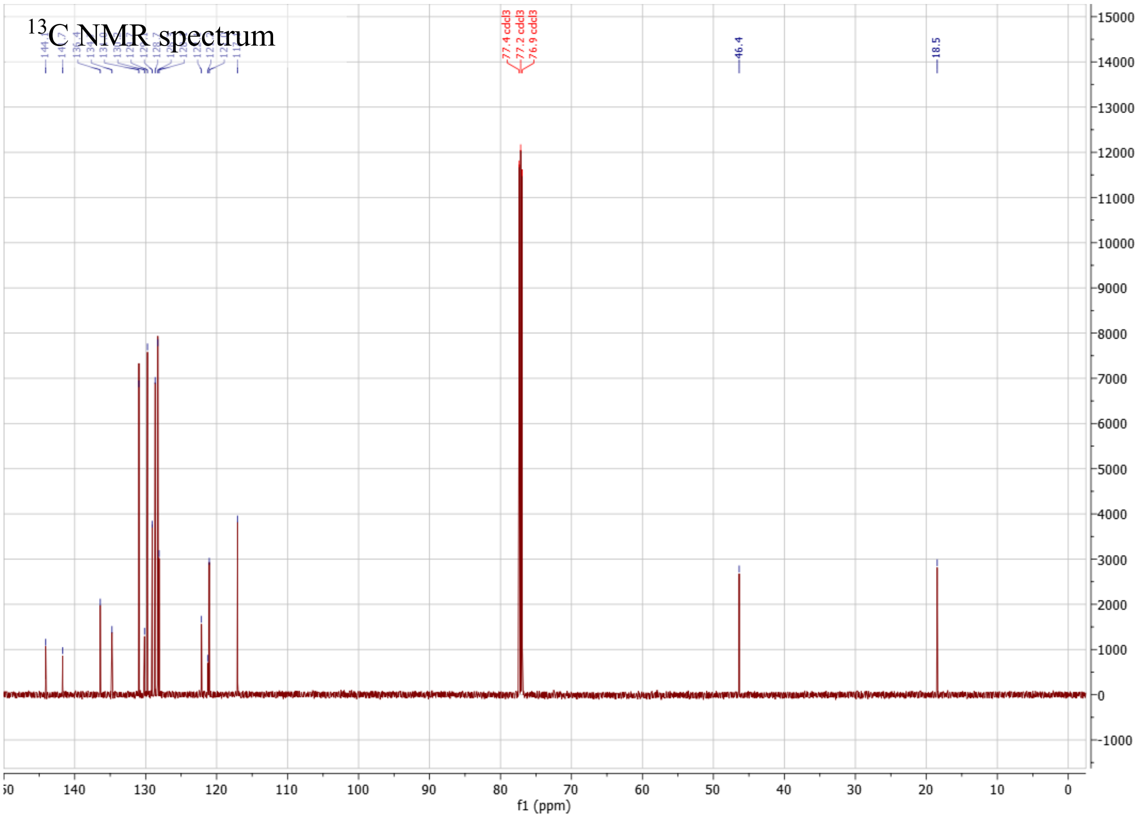

4-Azidopiperidine (40).

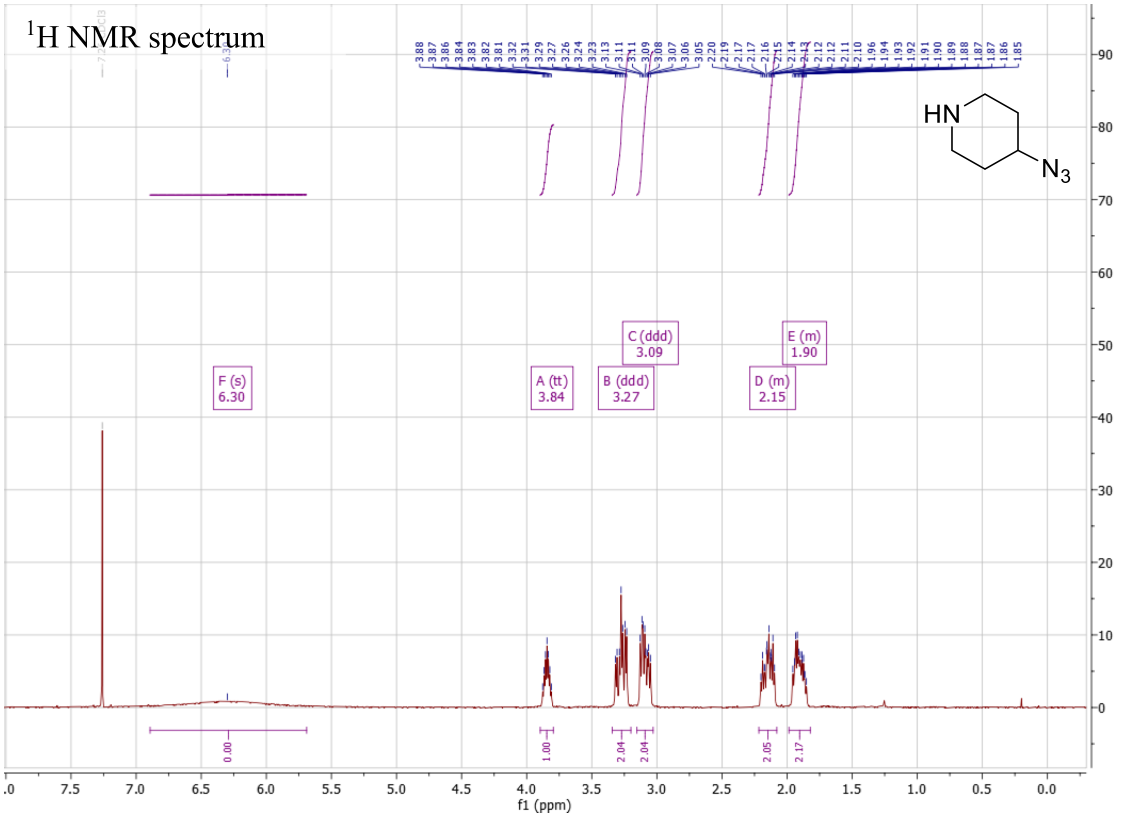

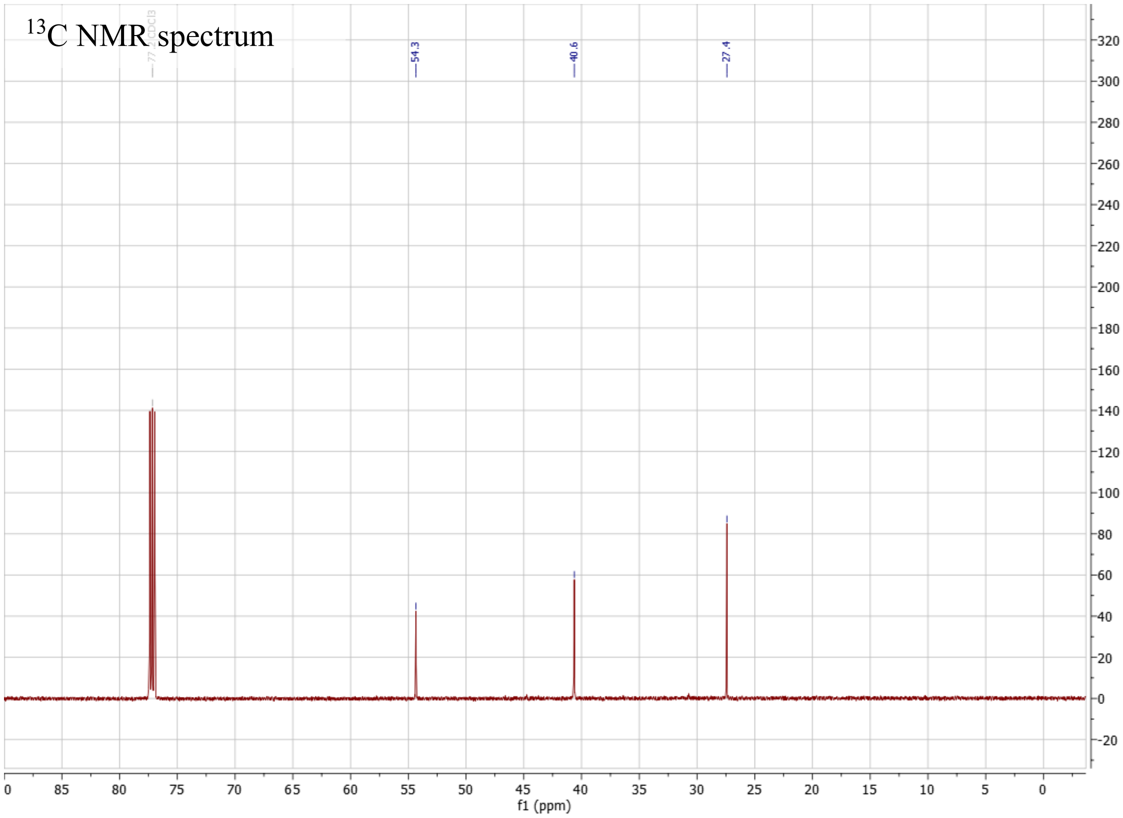

2-{4-[(4-Azidopiperidin-1-yl)methyl]phenyl}-3-(*o*-tolyl)imidazo[1,2-*a*]pyridine (43).

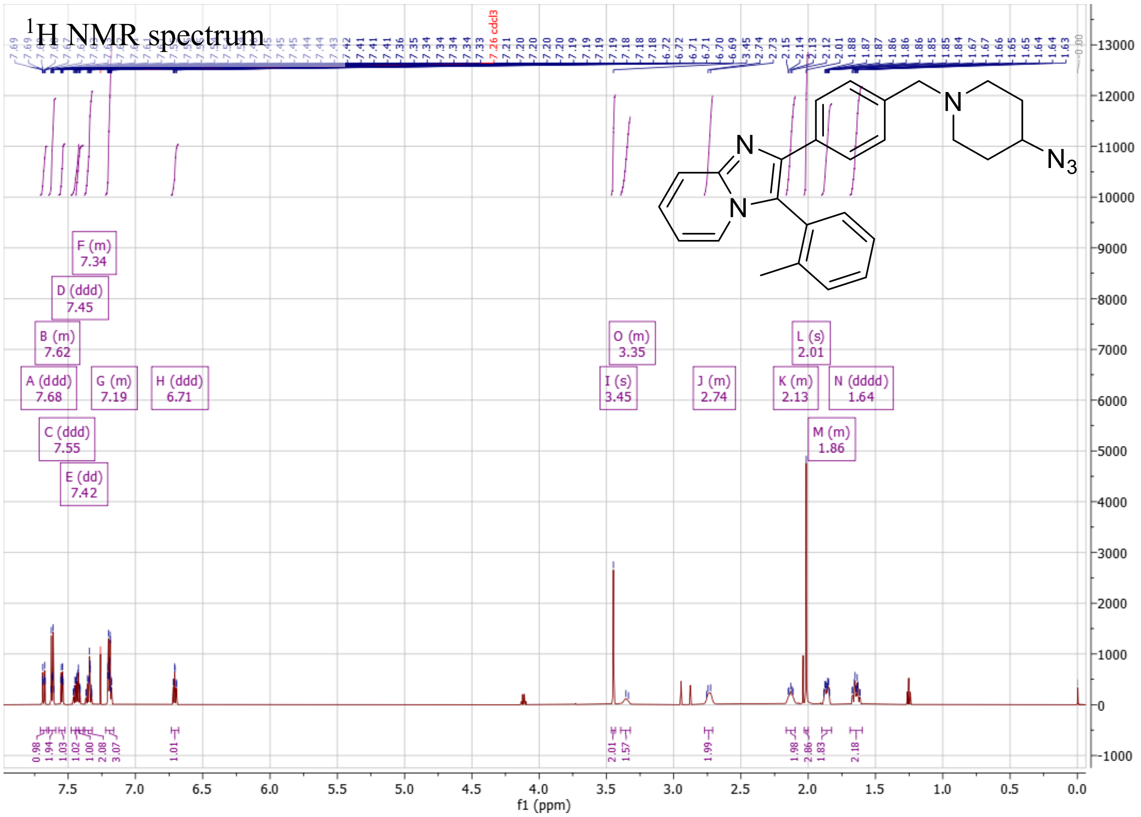

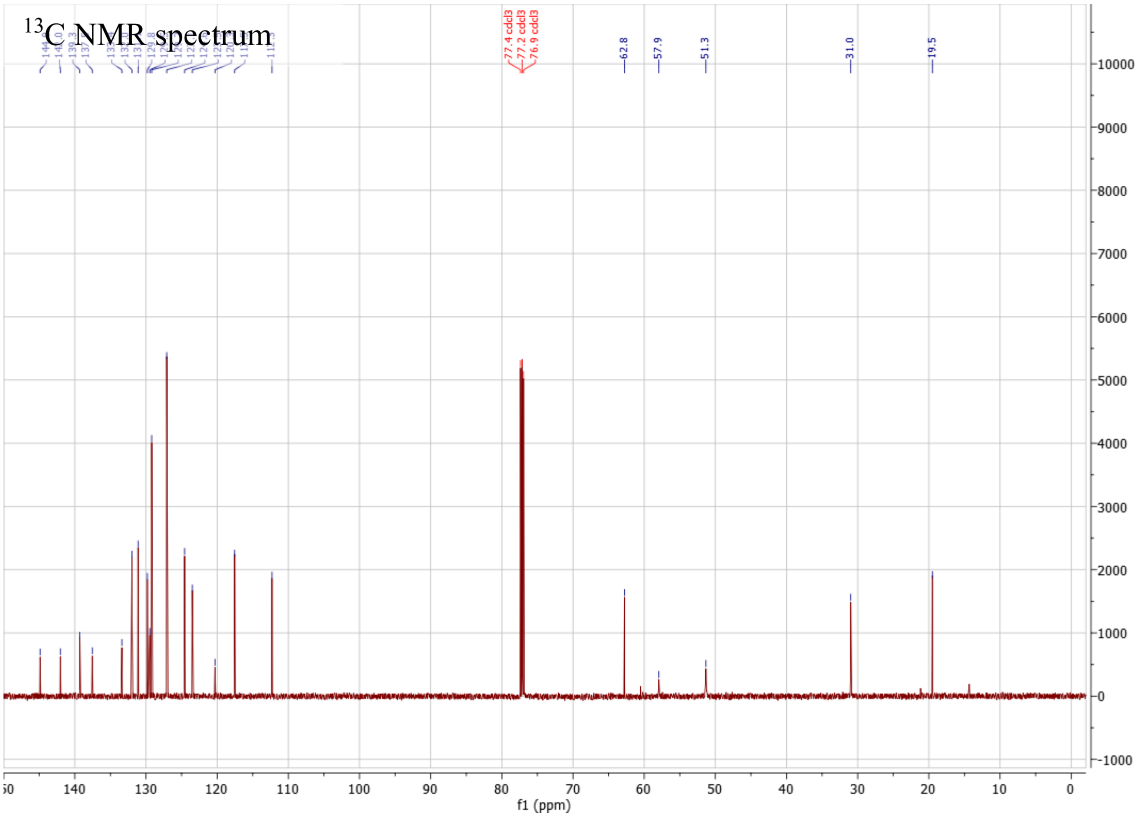

2-{4-[(4-Azidopiperidin-1-yl)methyl]phenyl}-3-(*m*-tolyl)imidazo[1,2-*a*]pyridine (44).

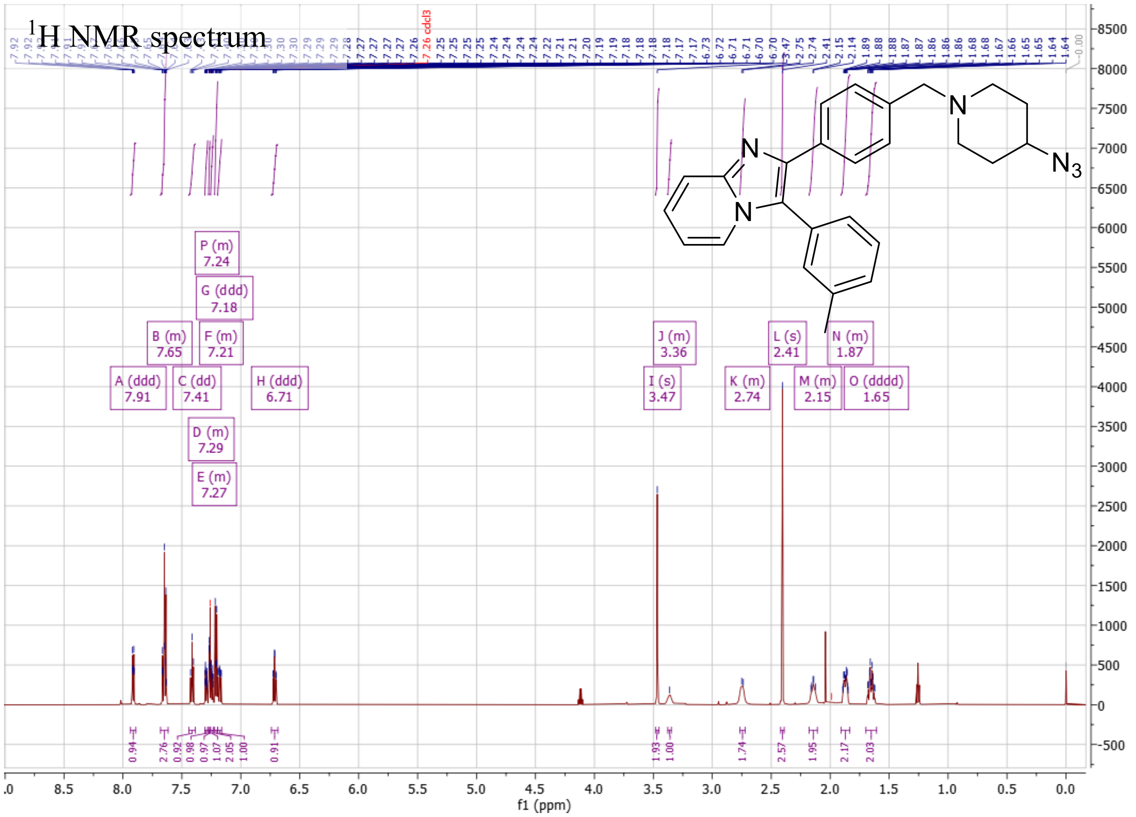

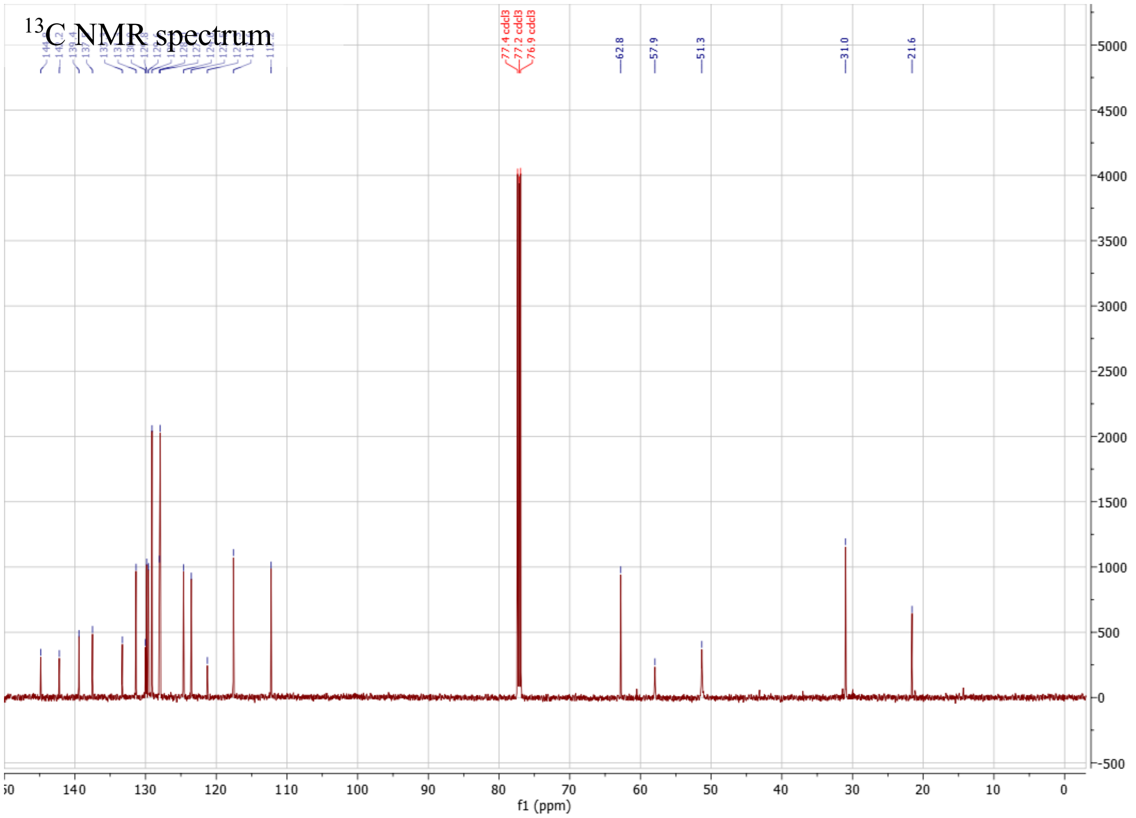

2-{4-[(4-Azidopiperidin-1-yl)methyl]phenyl}-6-methyl-3-phenylimidazo[1,2-*a*]pyridine (45).

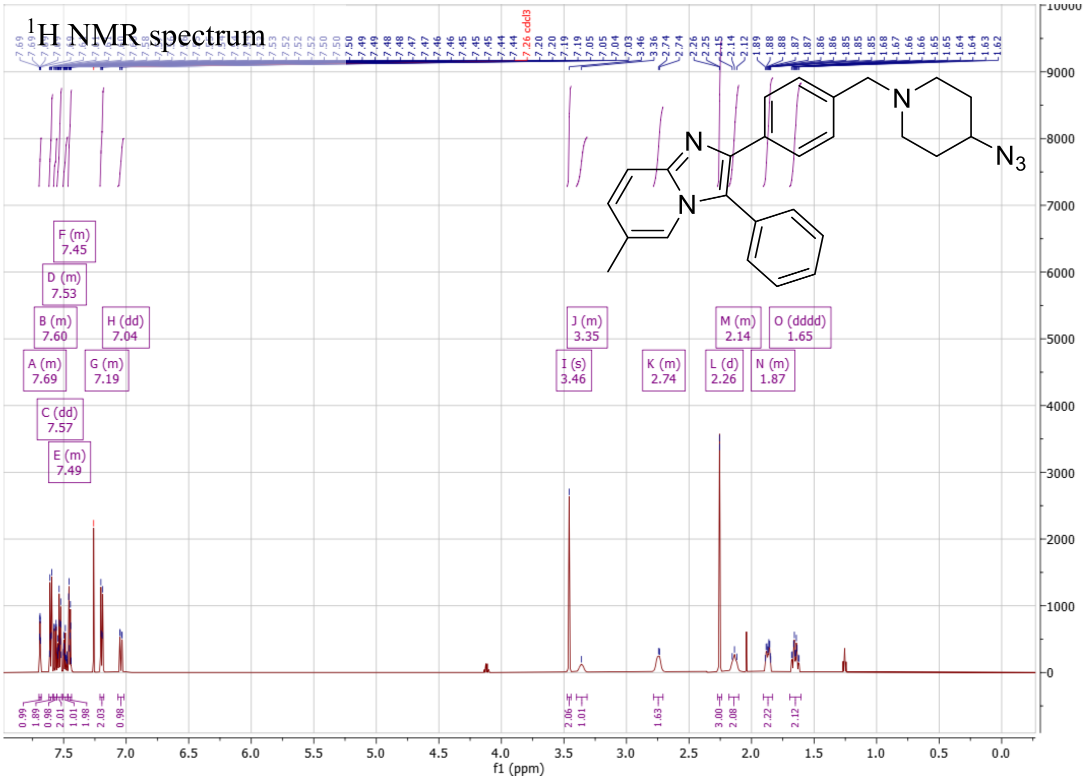

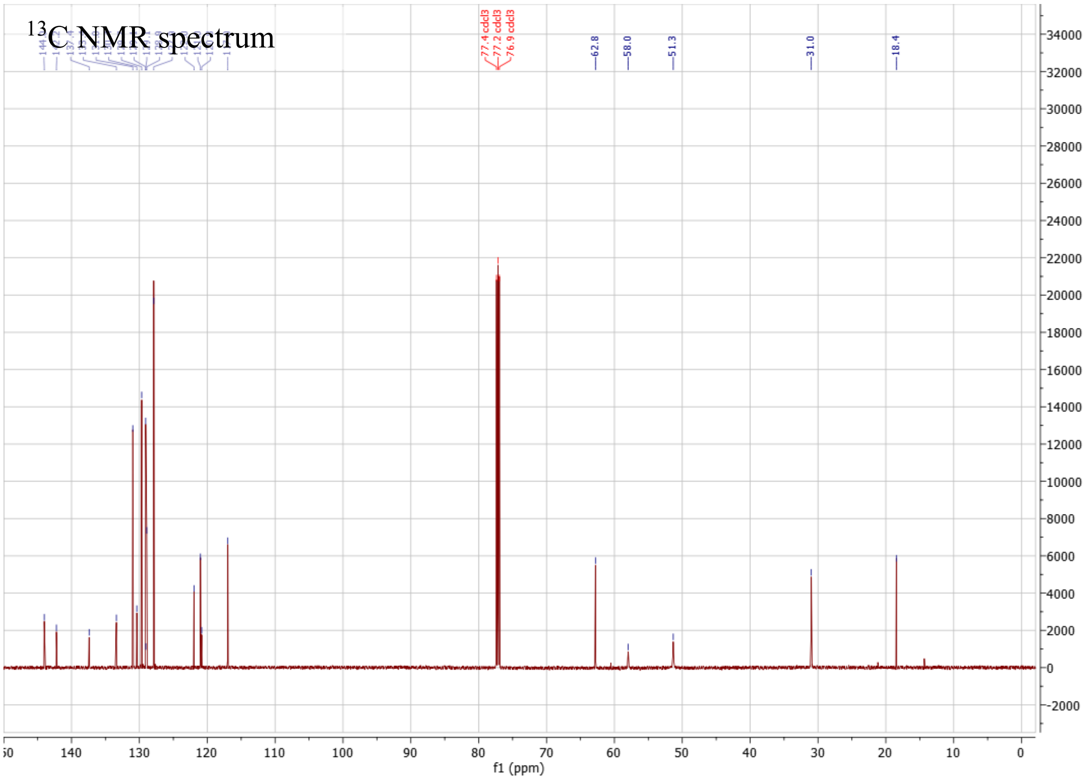

_N_-(2-Ethynylphenyl)acrylamide (51).

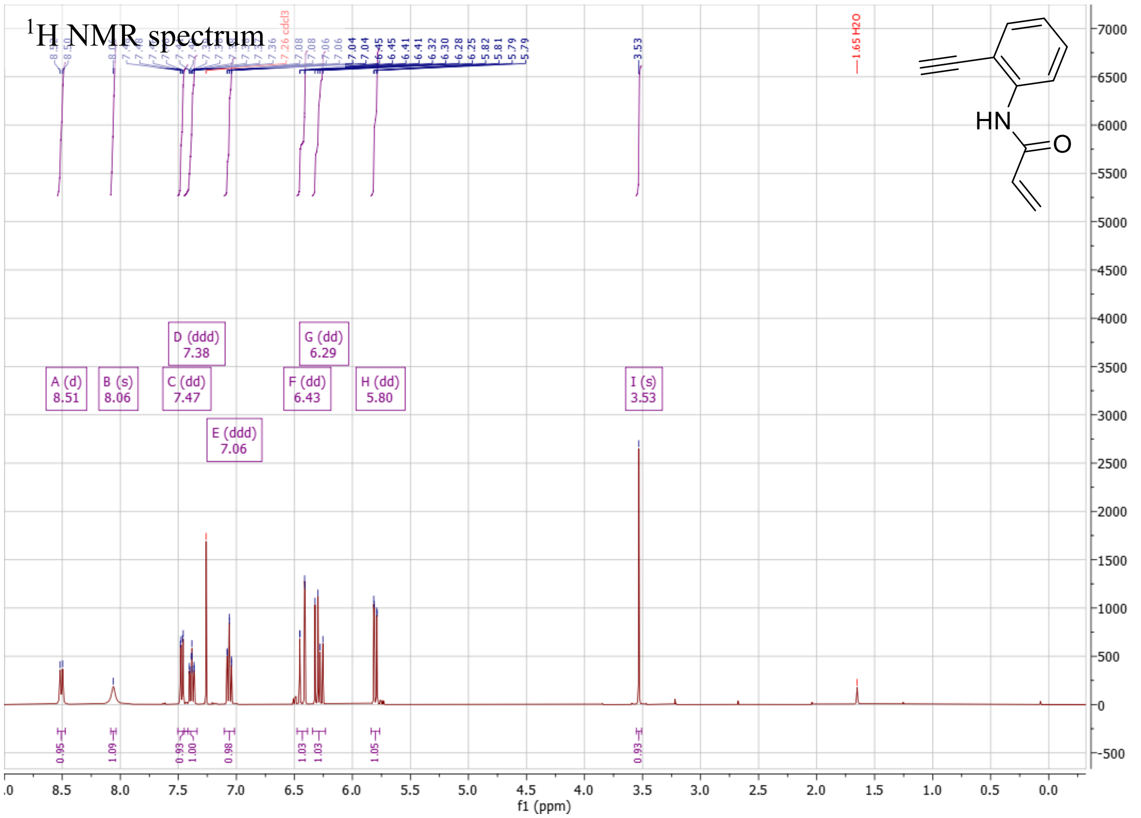

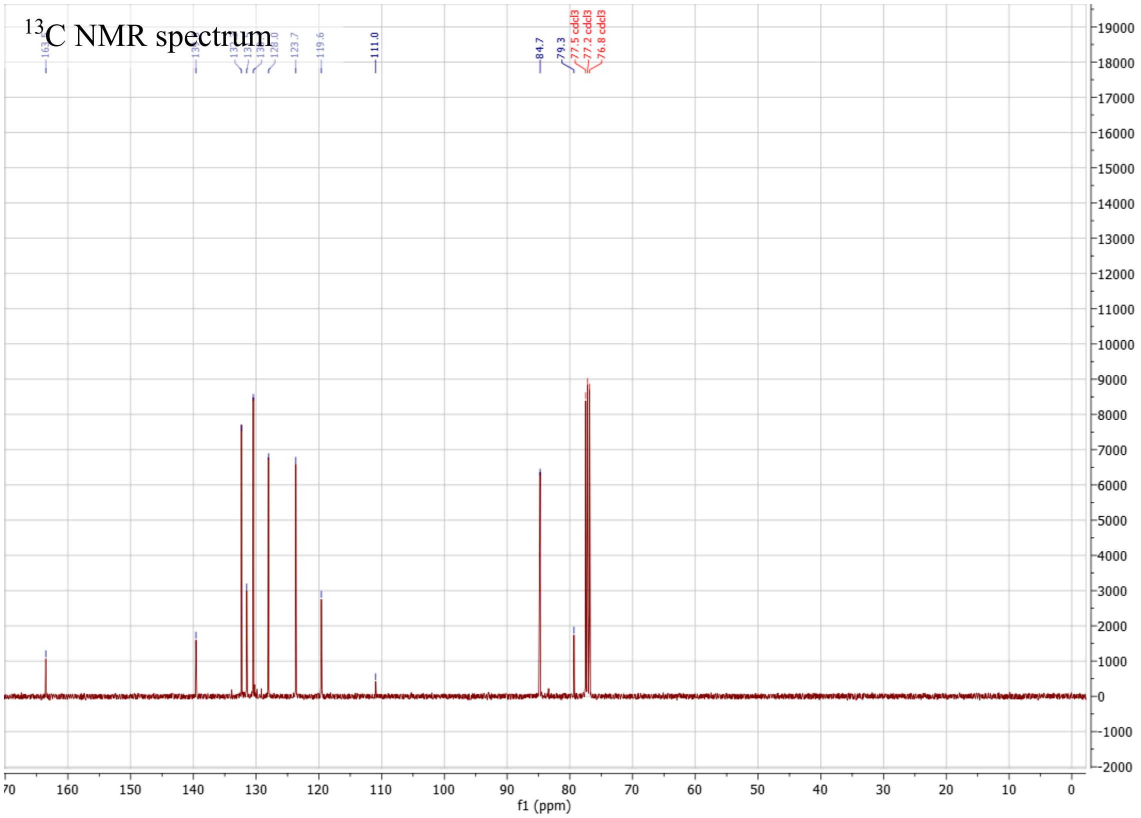

N-(4-Ethynylphenyl)acrylamide (53).

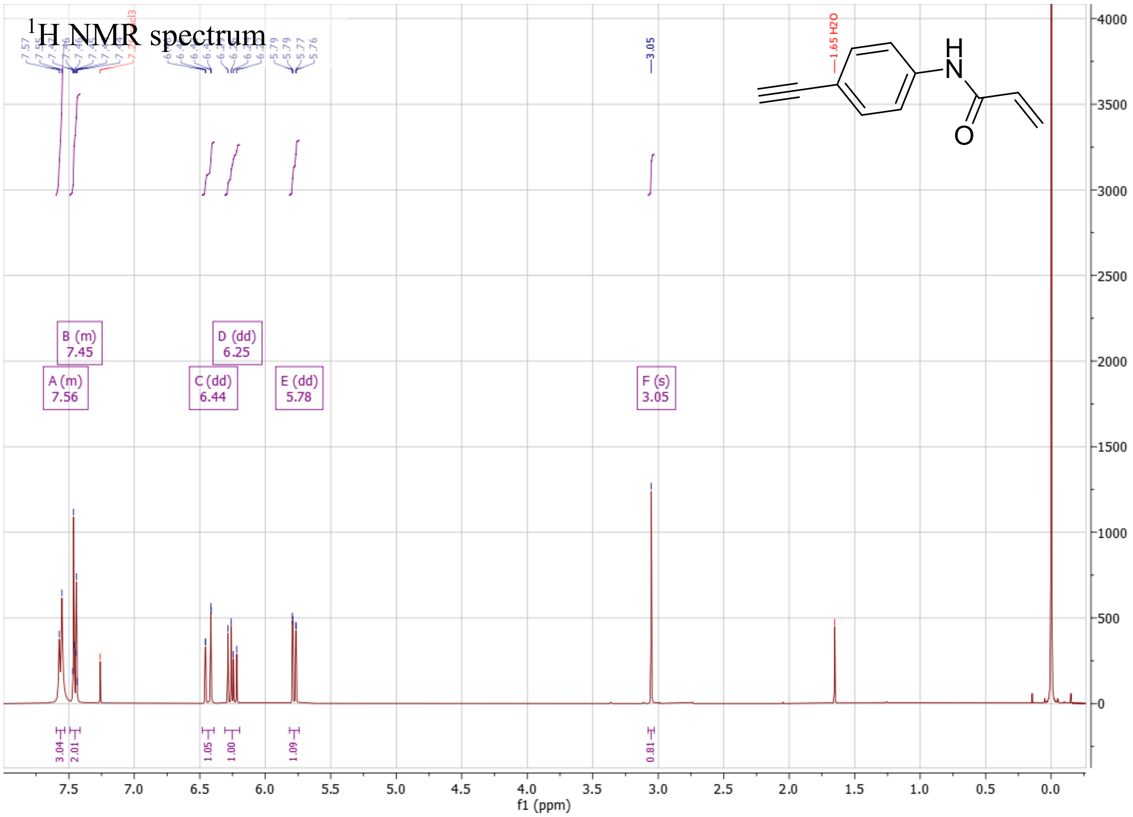

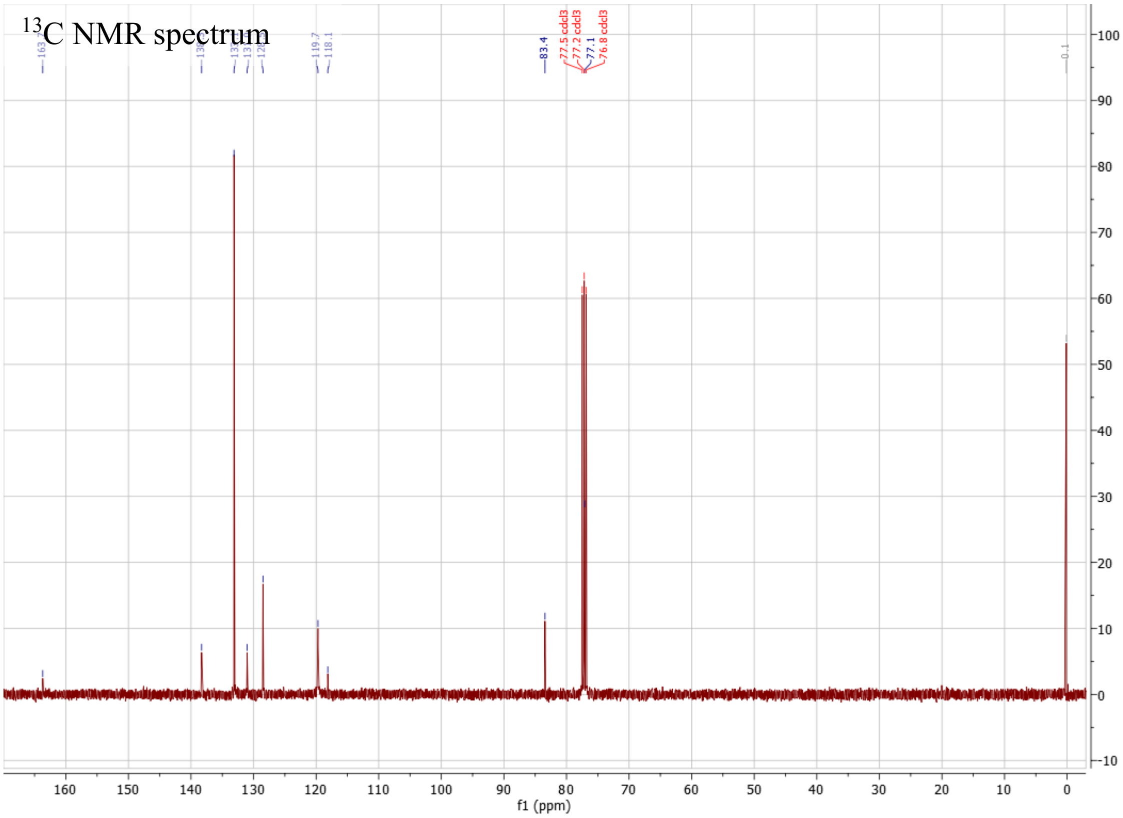

N-[2-(1-{1-[4-(3-Phenylimidazo[1,2-*a*]pyridin-2-yl)benzyl]piperidin-4-yl}-1*H*-1,2,3-triazol-4-yl)phenyl]acrylamide (4).

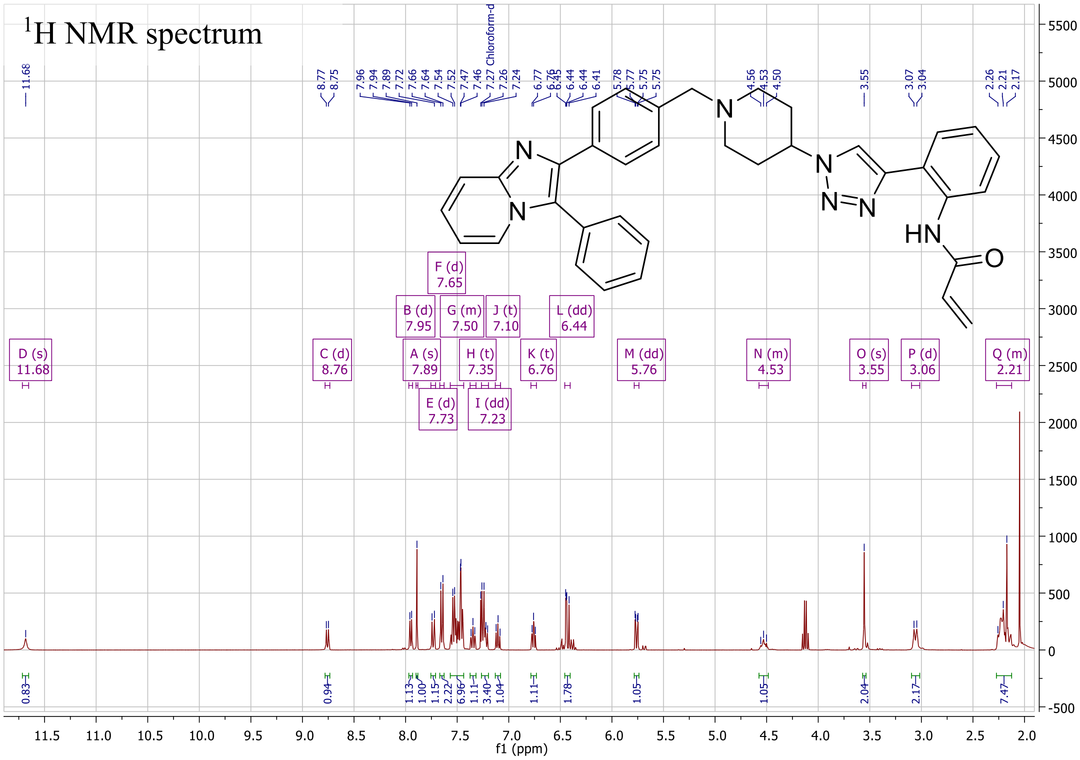

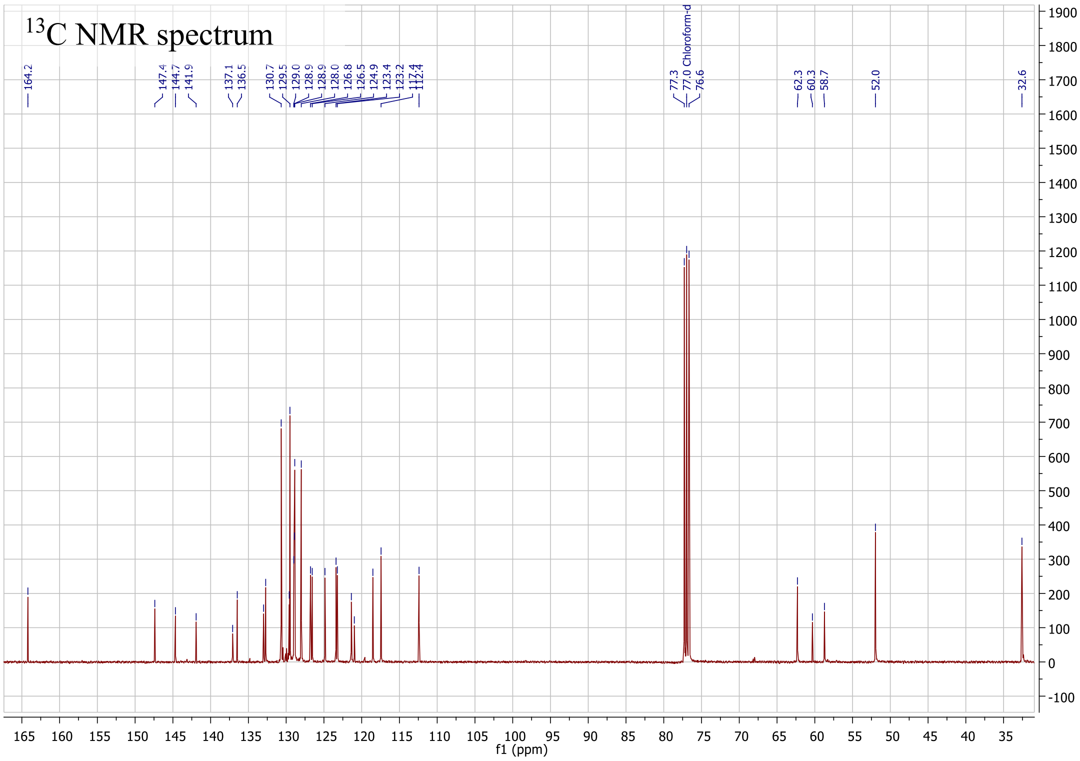

***N*-[4-(1-{1-[4-(3-phenylimidazo[1,2-*a*]pyridin-2-yl)benzyl]piperidin-4-yl}-1*H*-1,2,3-triazol-4-yl)phenyl]acrylamide (5).**

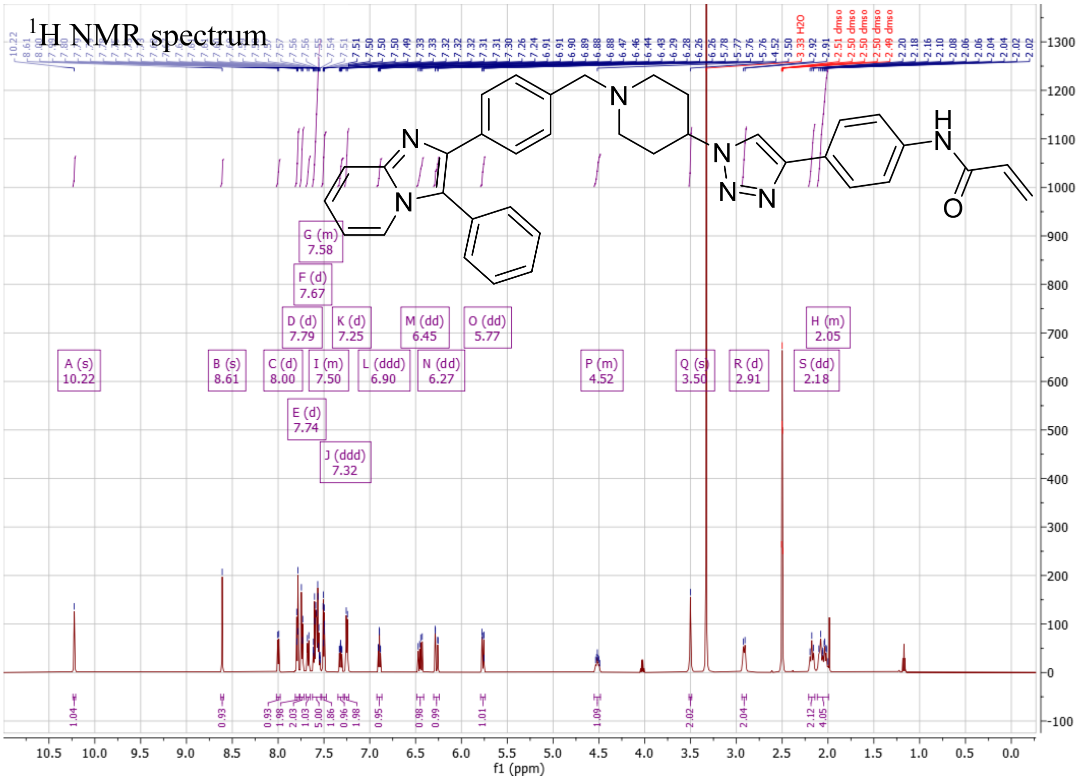

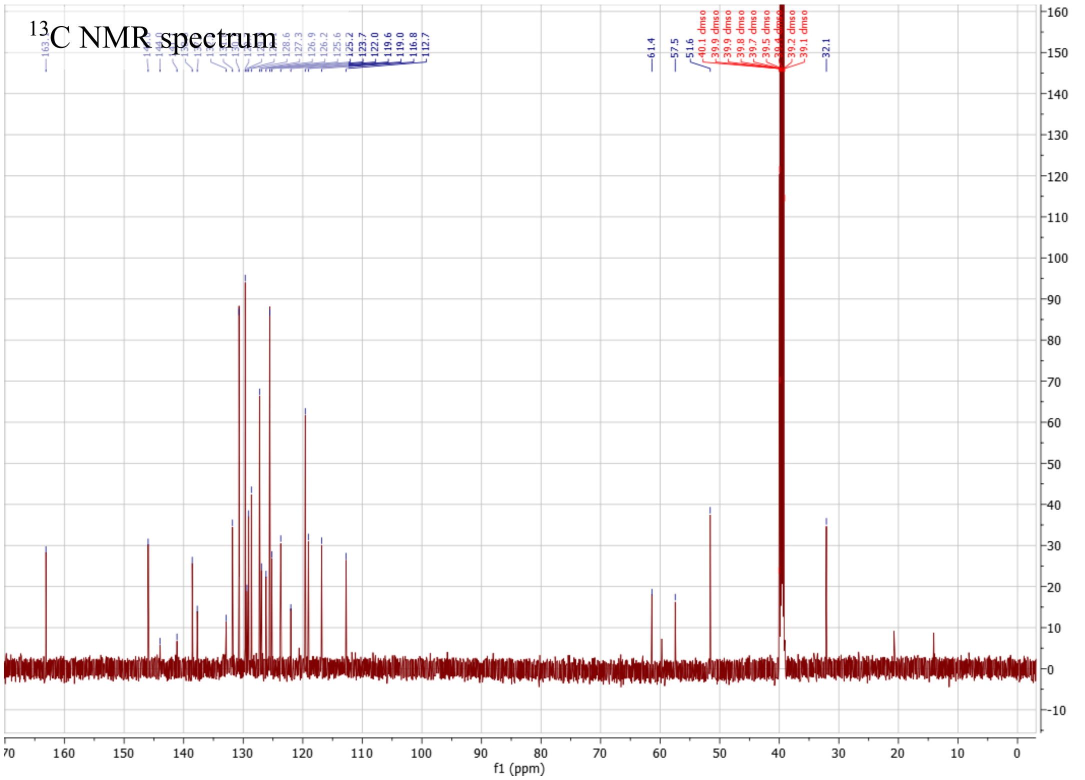

***N*-{2-[1-(1-{4-[3-(*o*-Tolyl)imidazo[1,2-*a*]pyridin-2-yl]benzyl}piperidin-4-yl)-1*H*-1,2,3-triazol-4-yl]phenyl}acrylamide (6).**

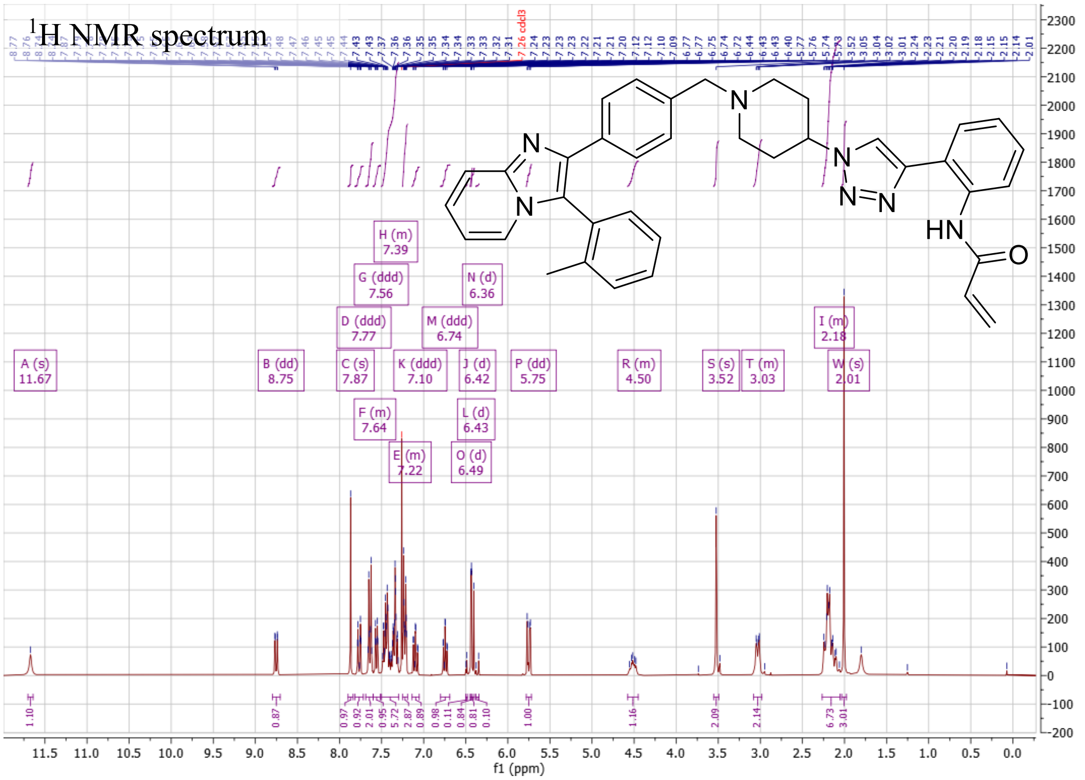

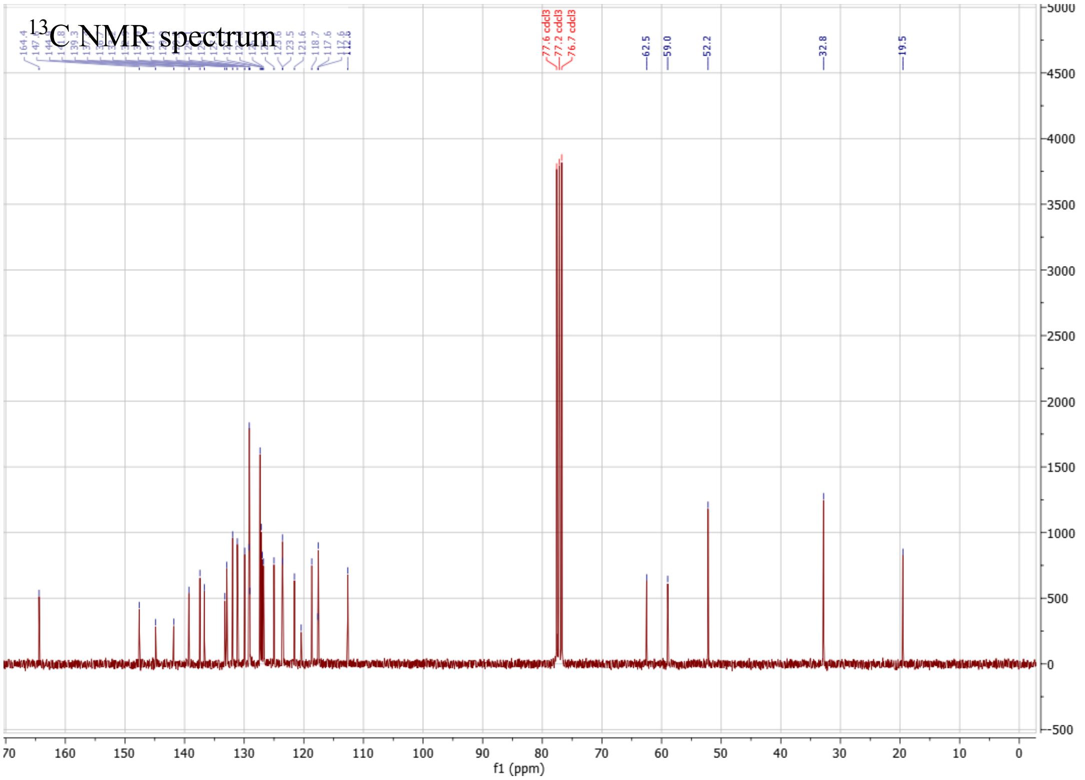

***N*-{3-[1-(1-{4-[3-(*o*-Tolyl)imidazo[1,2-*a*]pyridin-2-yl]benzyl}piperidin-4-yl)-1*H*-1,2,3-triazol-4-yl]phenyl}acrylamide (7).**

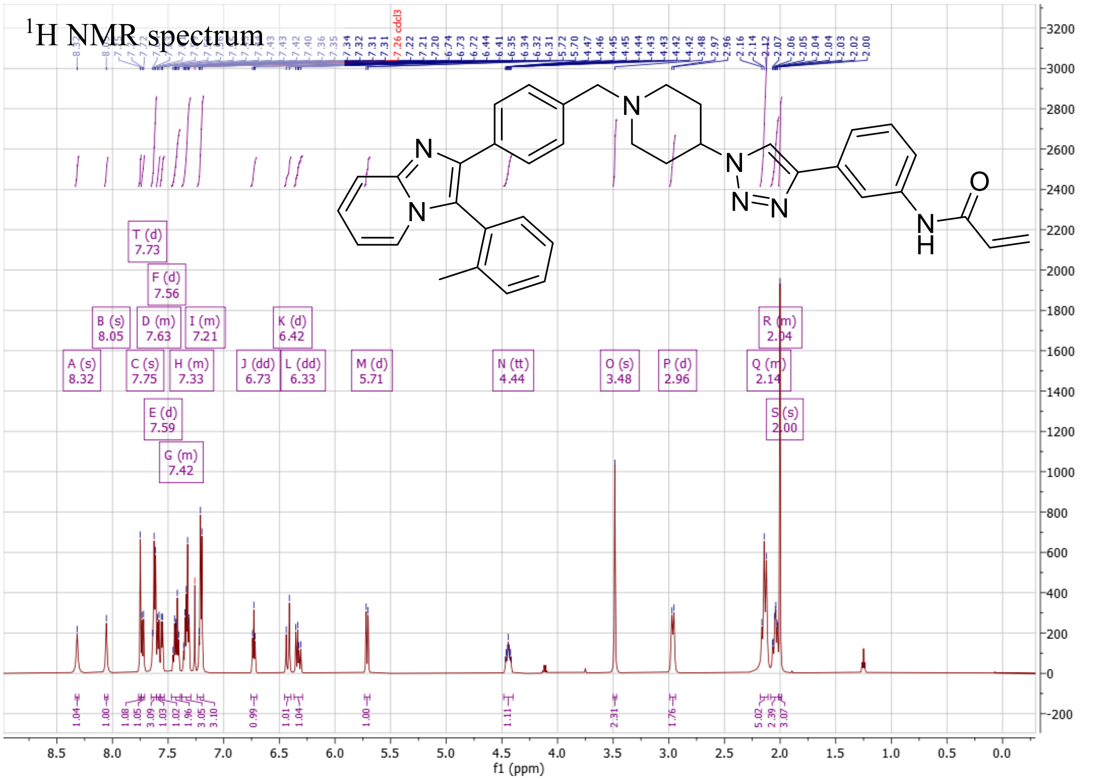

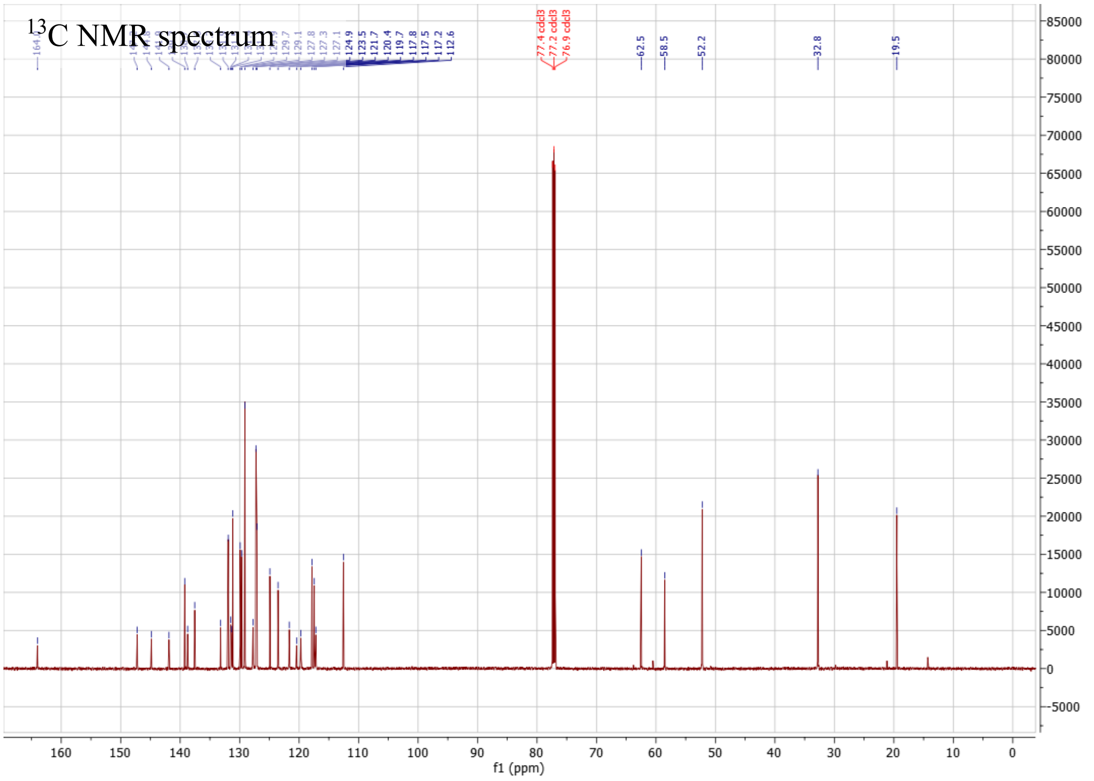

_N_-{4-[1-(1-{4-[3-(_o_-Tolyl)imidazo[1,2-_a_]pyridin-2-yl]benzyl}piperidin-4-yl)-1_H_-1,2,3-triazol-4-yl]phenyl}acrylamide (8).

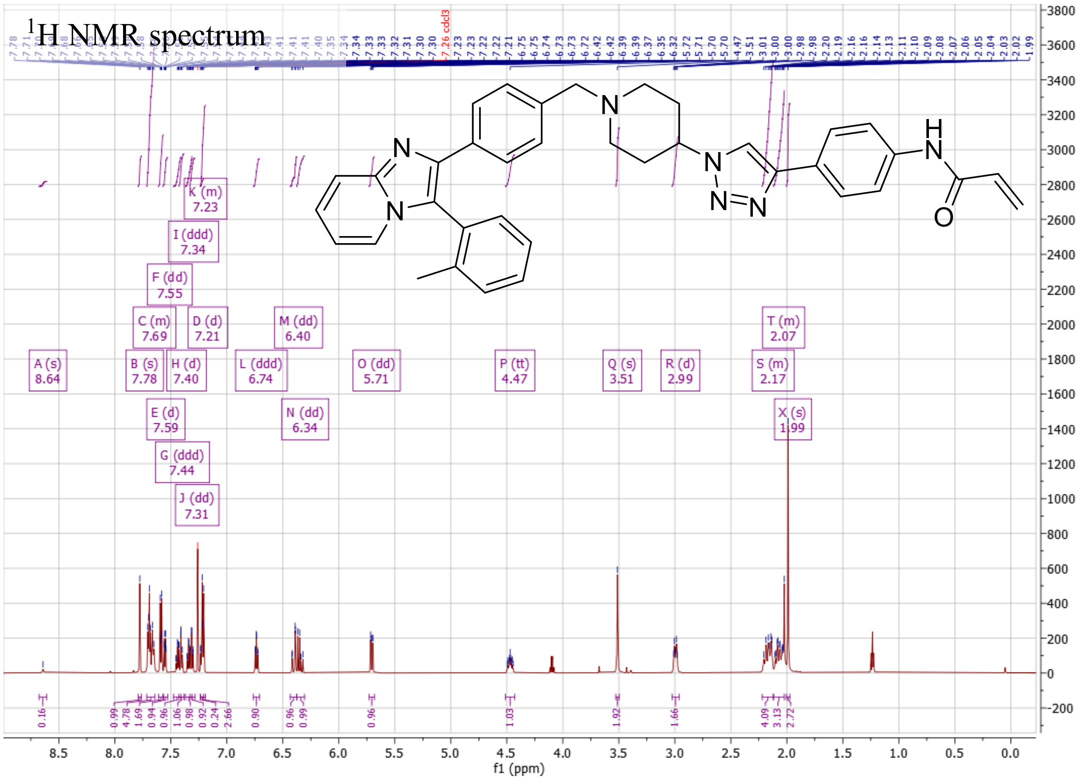

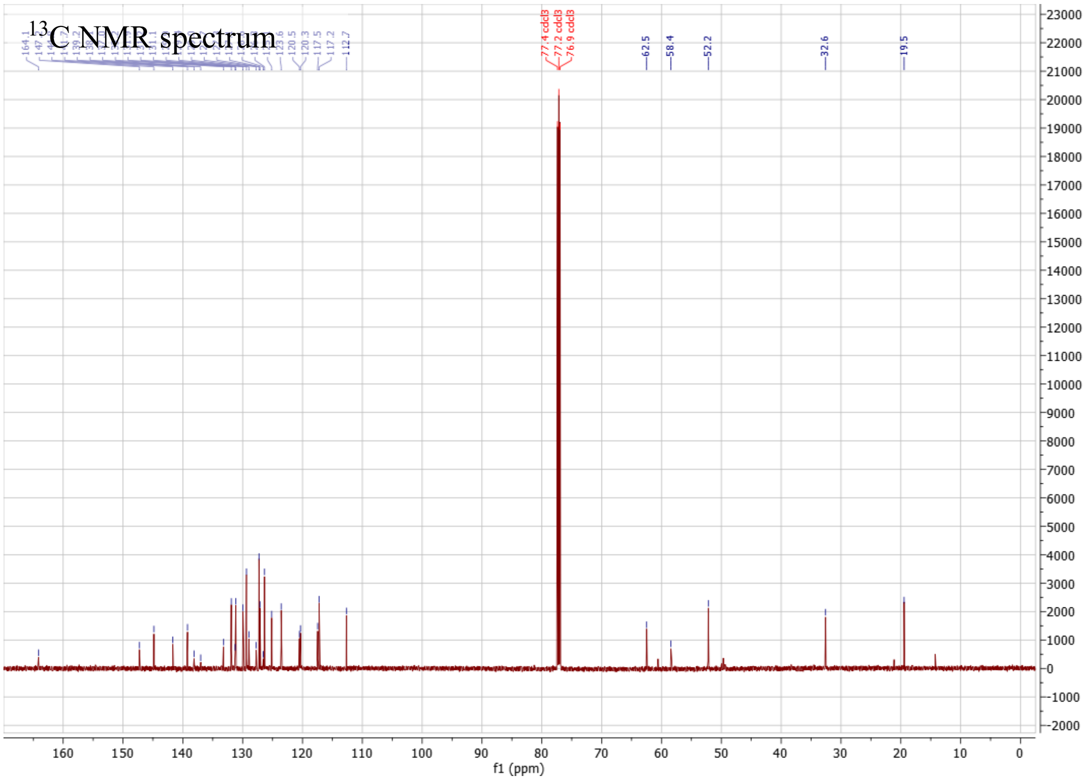

***N*-{3-[1-(1-{4-[3-(*m*-Tolyl)imidazo[1,2-*a*]pyridin-2-yl]benzyl}piperidin-4-yl)-1*H*-1,2,3-triazol-4-yl]phenyl}acrylamide (9).**

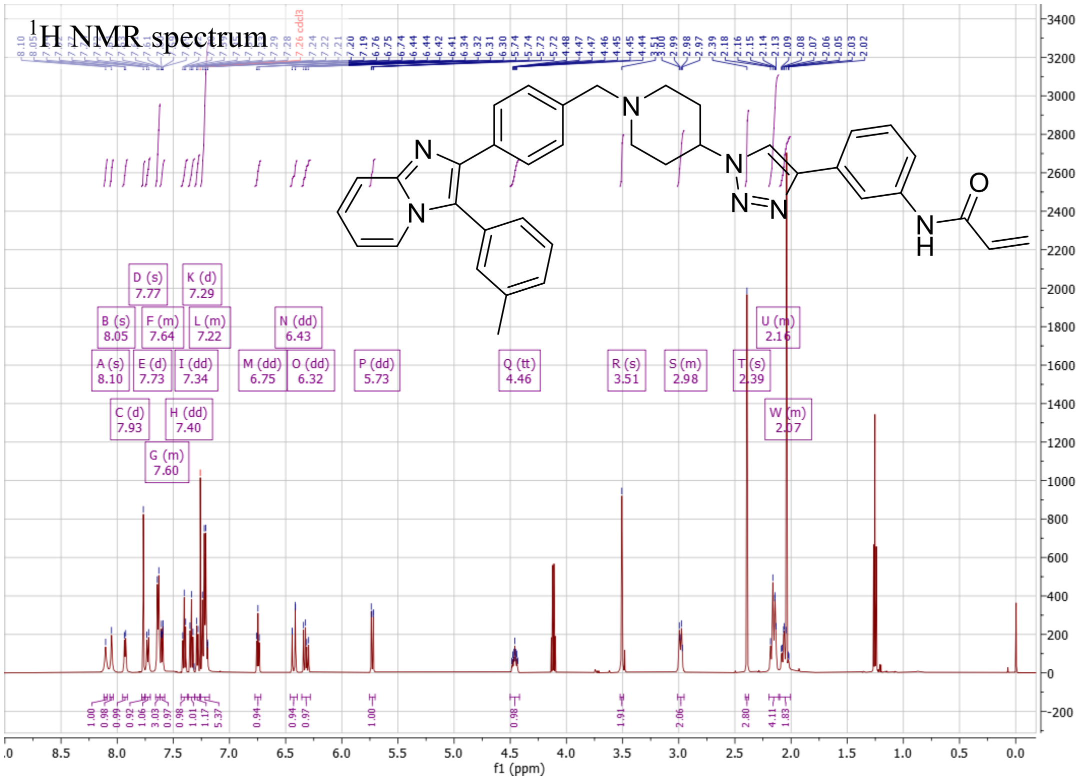

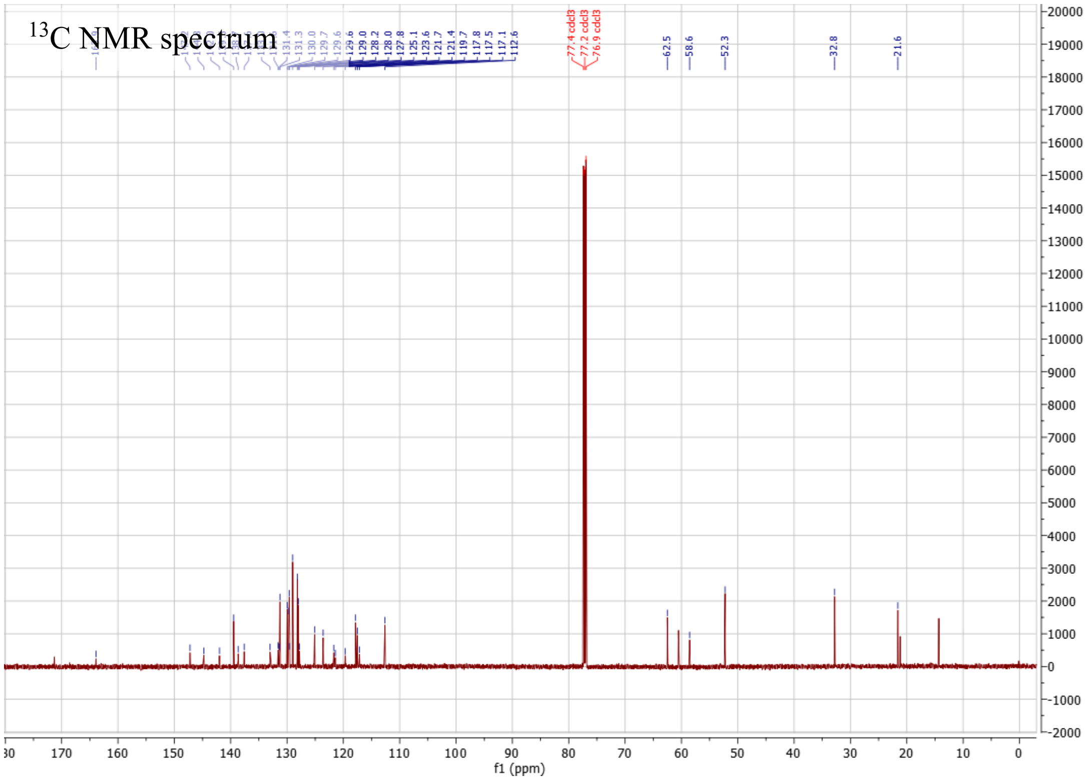

***N*-[3-(1-{1-[4-(6-Methyl-3-phenylimidazo[1,2-*a*]pyridin-2-yl)benzyl]piperidin-4-yl}-1*H*-1,2,3-triazol-4-yl)phenyl]acrylamide (10).**

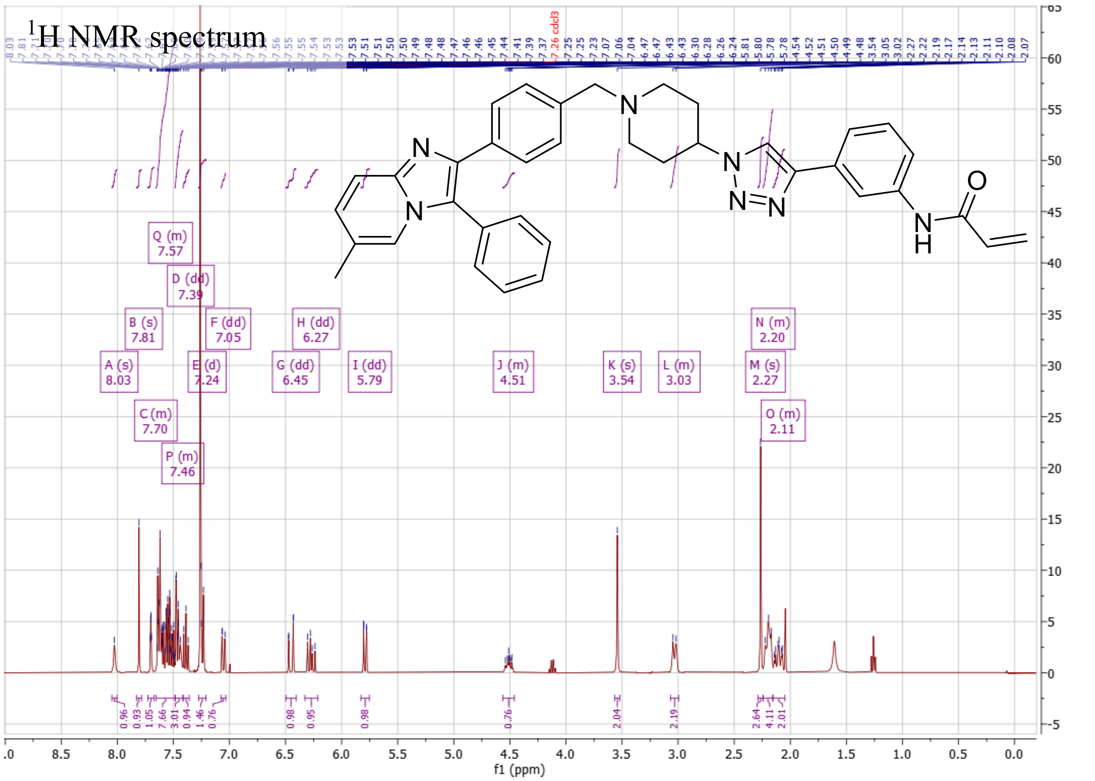

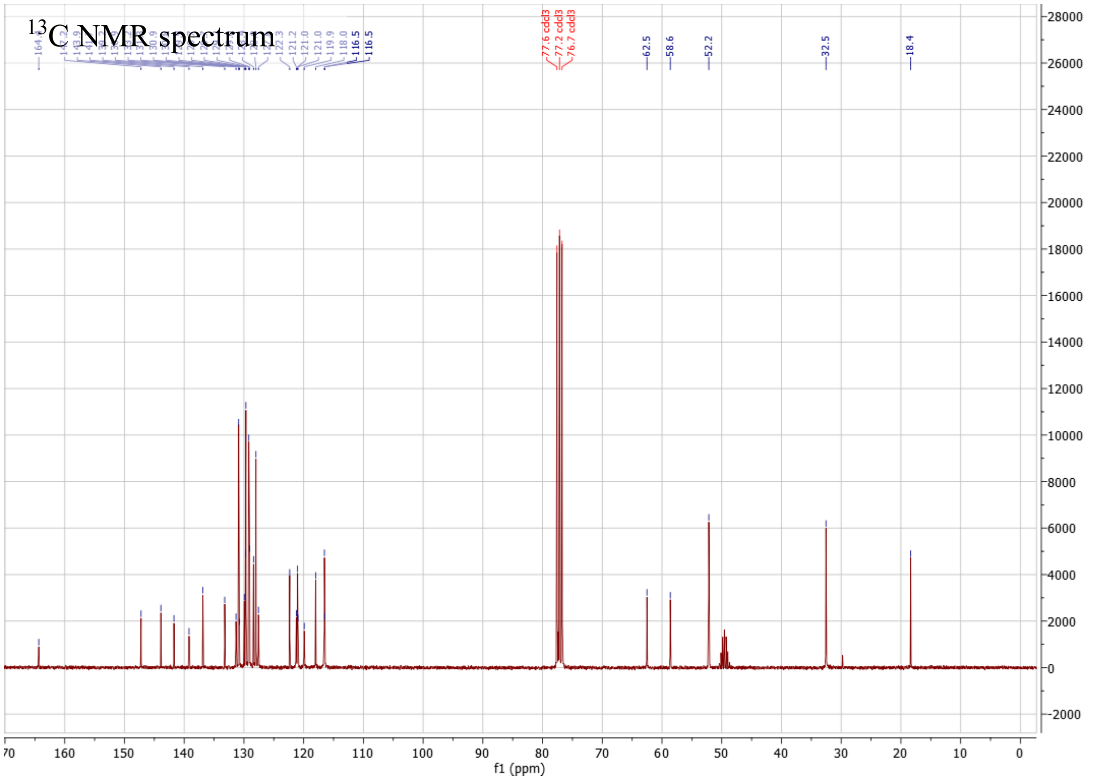

4-(5-Chloro-6-nitro-2-oxo-2,3-dihydro-1*H*-benzo[*d*]imidazol-1-yl)-piperidin-1-ium nitrate (55).

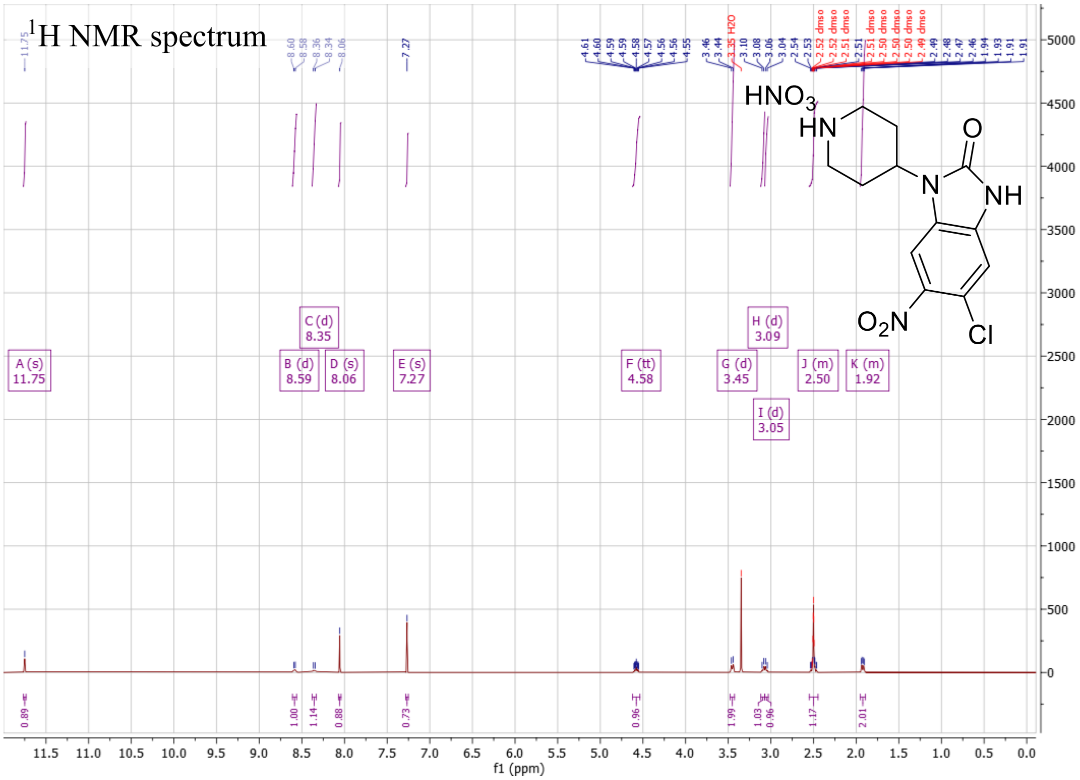

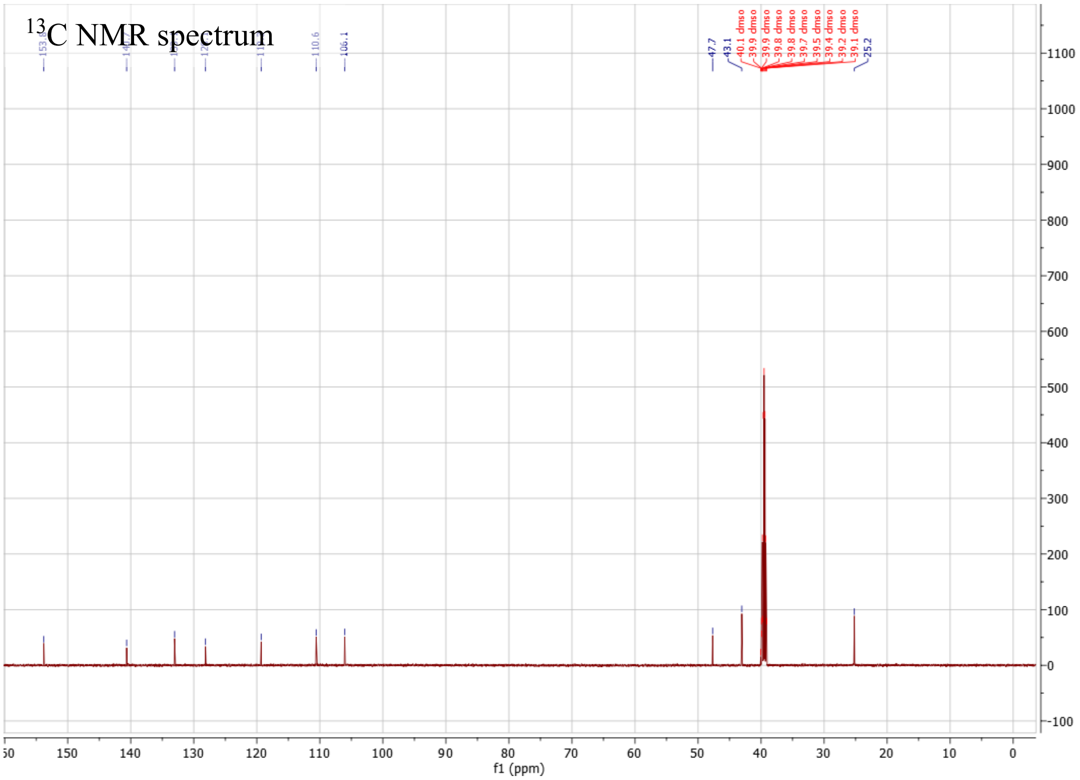

6-Amino-1-(piperidin-4-yl)-1,3-dihydro-2*H*-benzo[*d*]imidazol-2-one (56).

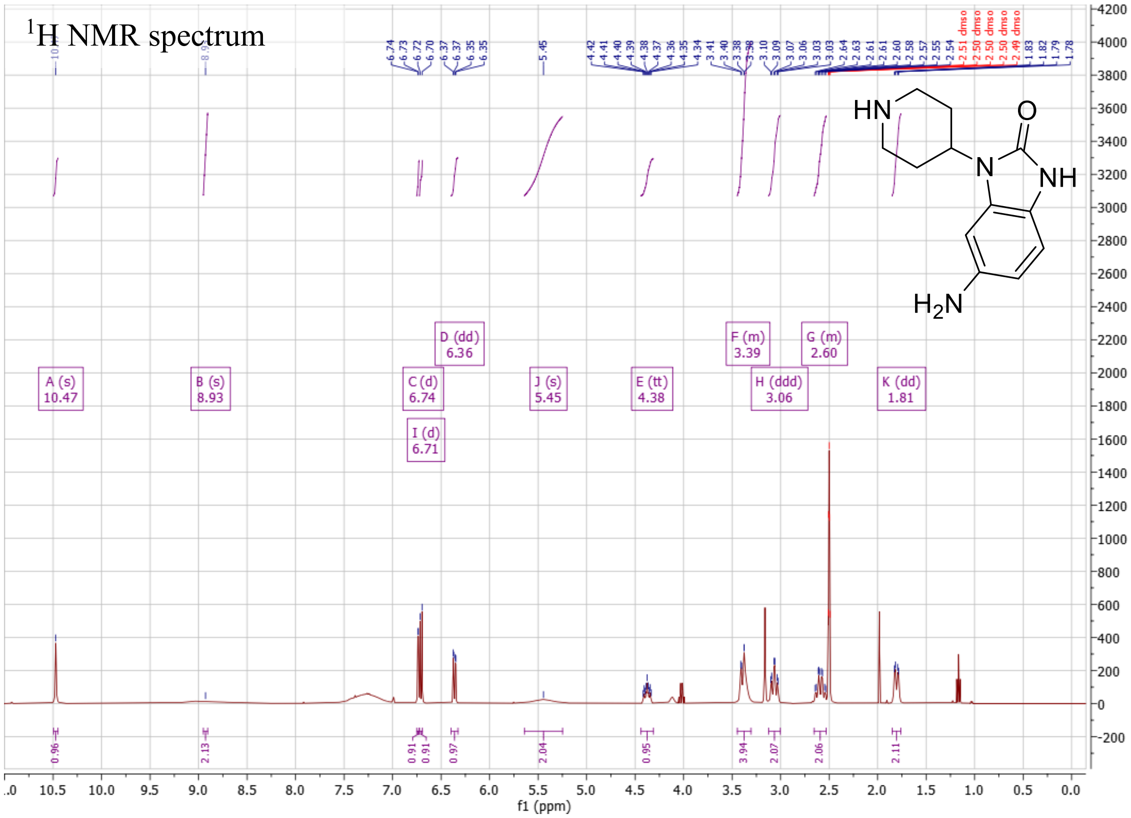

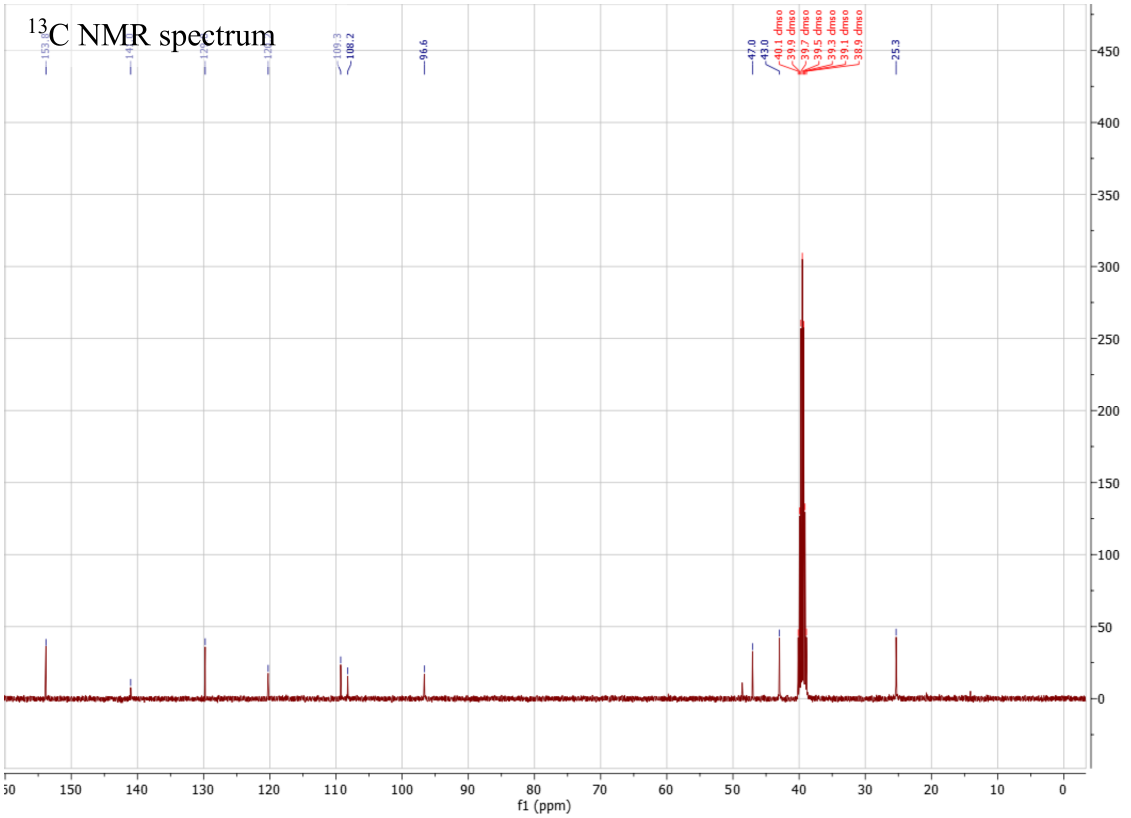

***tert*-Butyl [2-oxo-3-(piperidin-4-yl)-2,3-dihydro-1*H*-benzo[*d*]imidazol-5-yl]carbamate (57).**

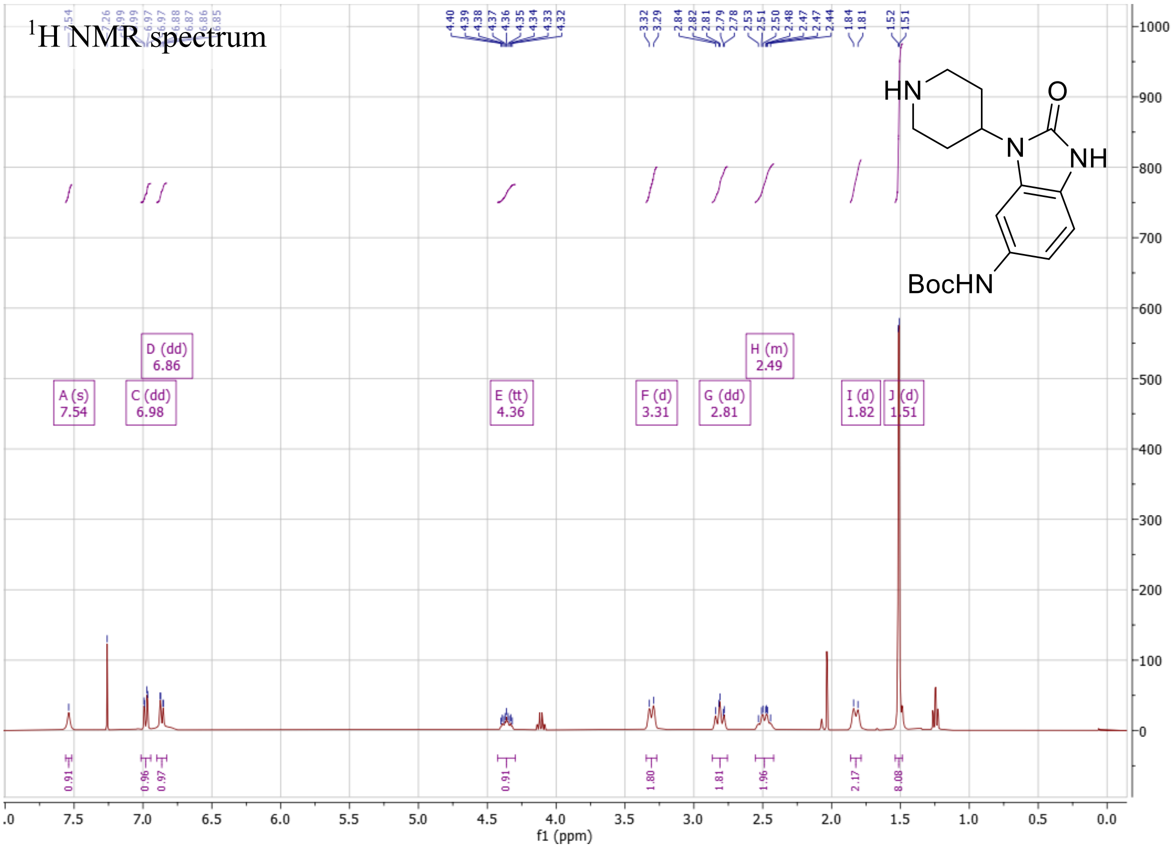

***tert*-Butyl (2-oxo-3-{1-[4-(3-phenylimidazo[1,2-*a*]pyridin-2-yl)benzyl]piperidin-4-yl}-2,3-dihydro-1*H*-benzo[*d*]imidazol-5-yl)carbamate (58).**

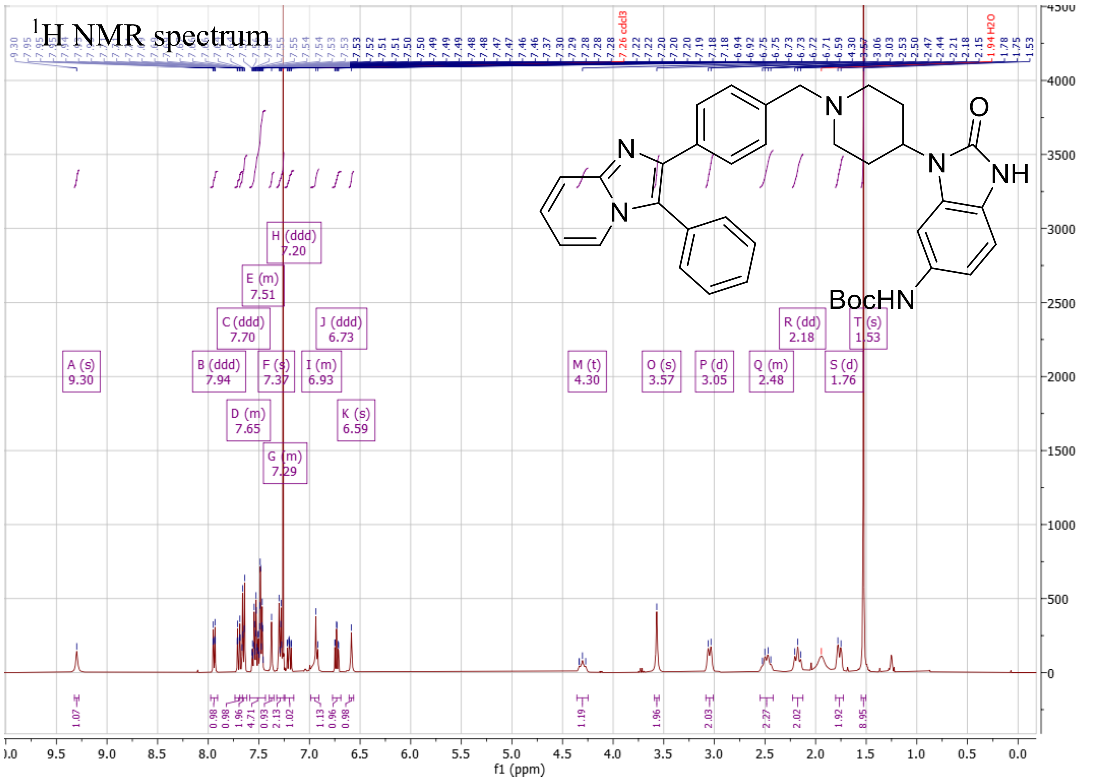

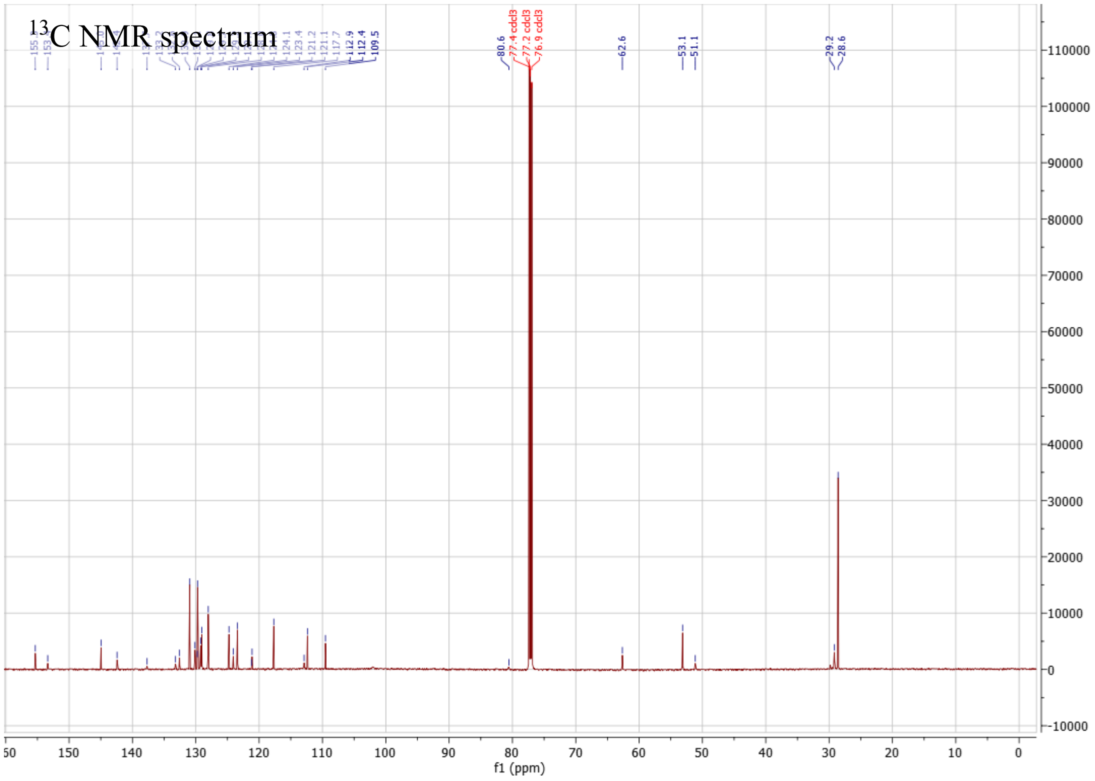

6-Amino-1-{1-[4-(3-phenylimidazo[1,2-*a*]pyridin-2-yl)benzyl]piperidin-4-yl}-1,3-dihydro-2*H*-benzo[*d*]imidazol-2-one (59).

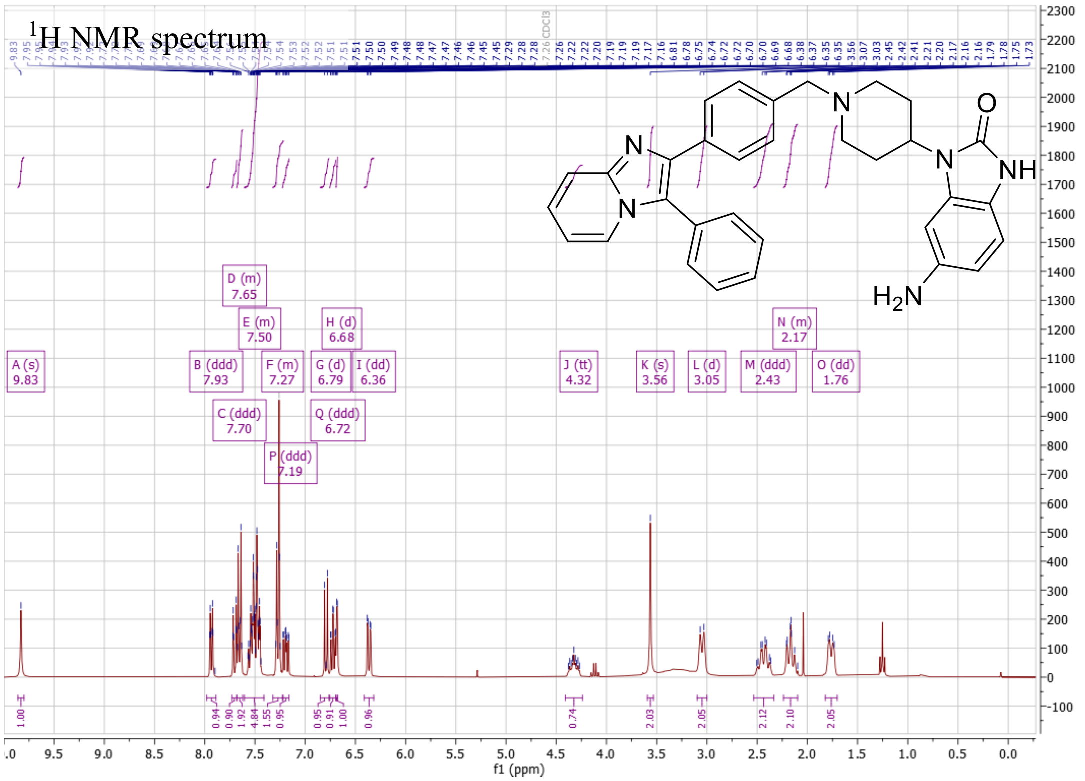

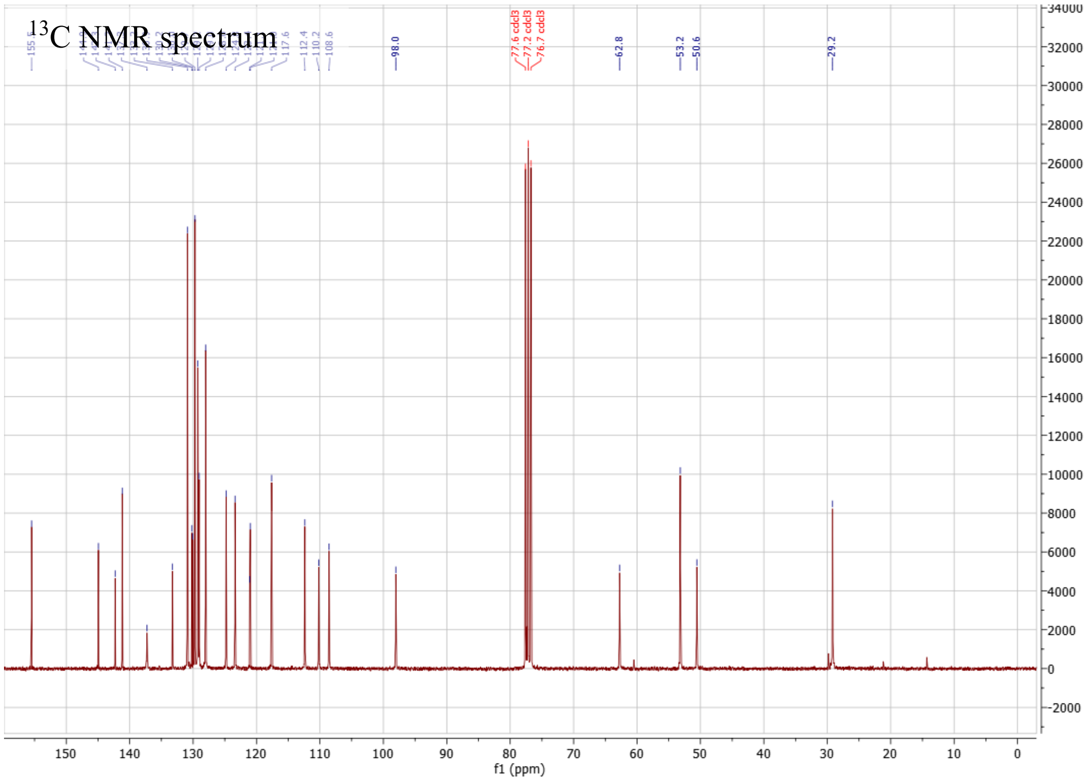

_N_-(2-Oxo-3-{1-[4-(3-phenylimidazo[1,2-_a_]pyridin-2-yl)benzyl]piperidin-4-yl}-2,3-dihydro-1_H_-benzo[_d_]imidazol-5-yl)acrylamide (11).

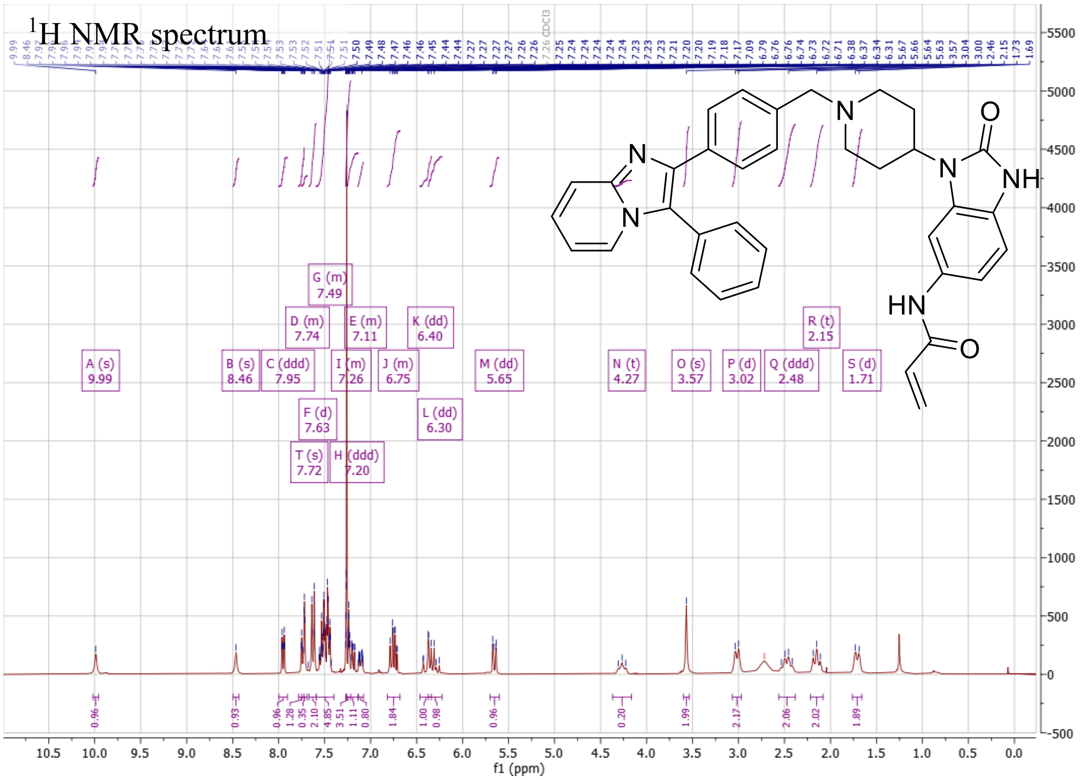

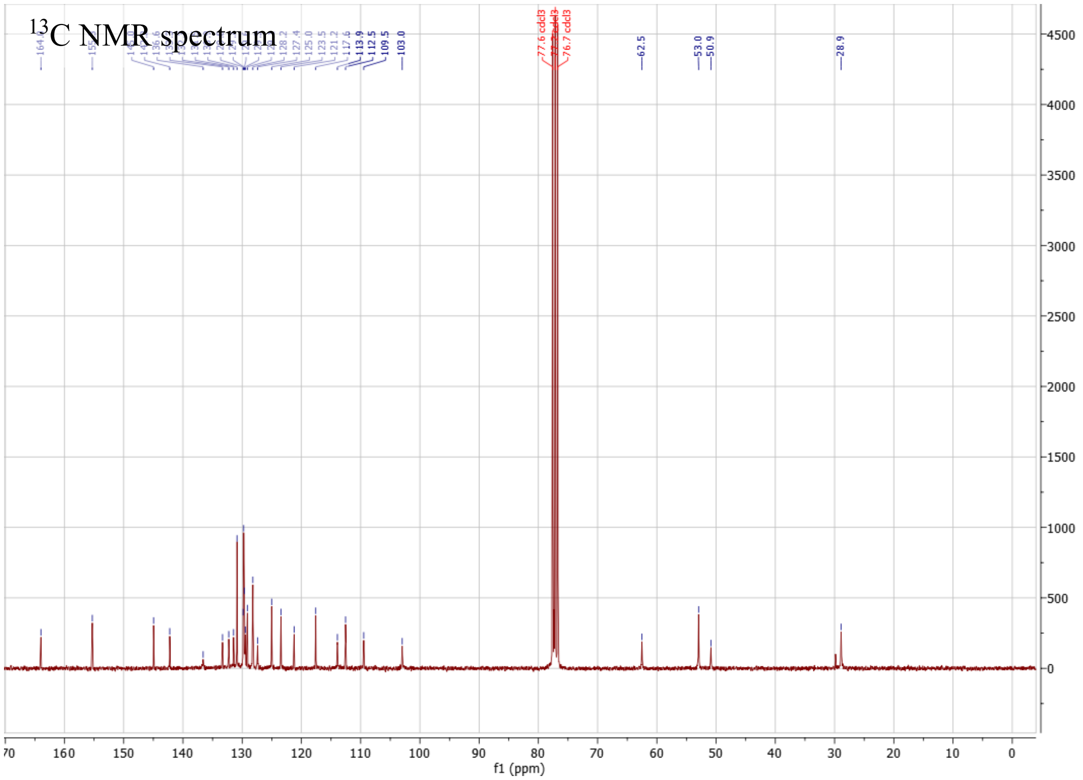

5-Chloro-6-nitro-1-{1-[4-(3-phenylimidazo[1,2-*a*]pyridin-2-yl)benzyl]piperidin-4-yl}-1,3-dihydro-2*H*-benzo[*d*]imidazol-2-one (60).

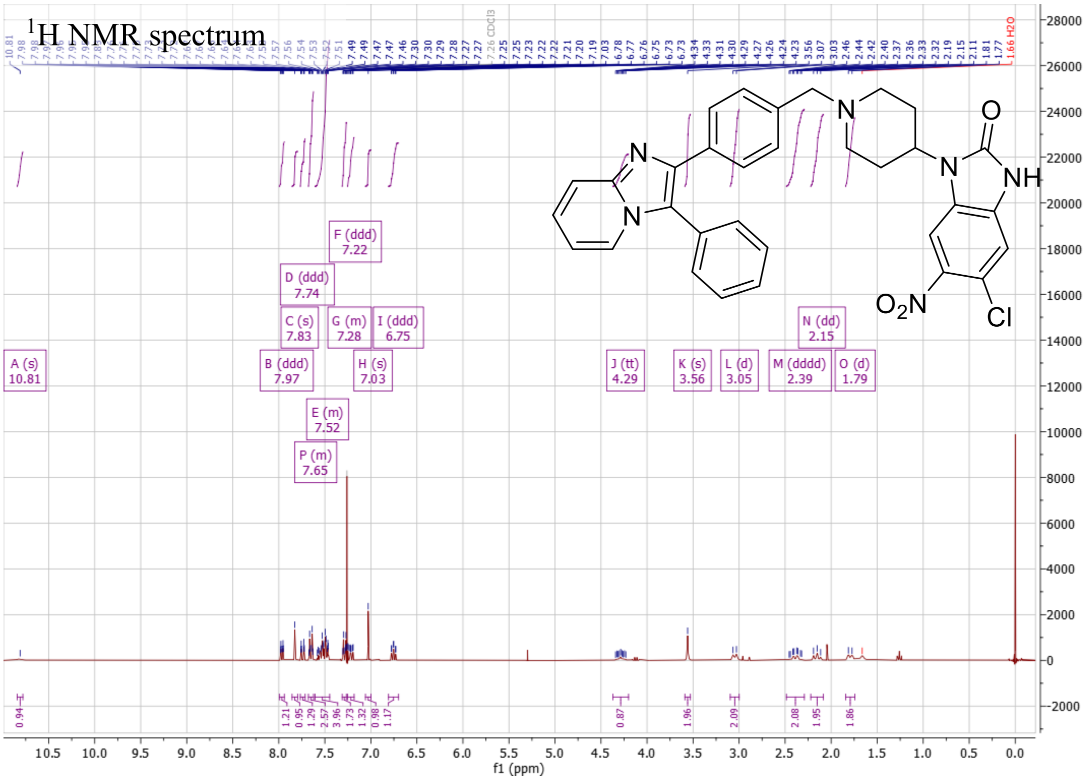

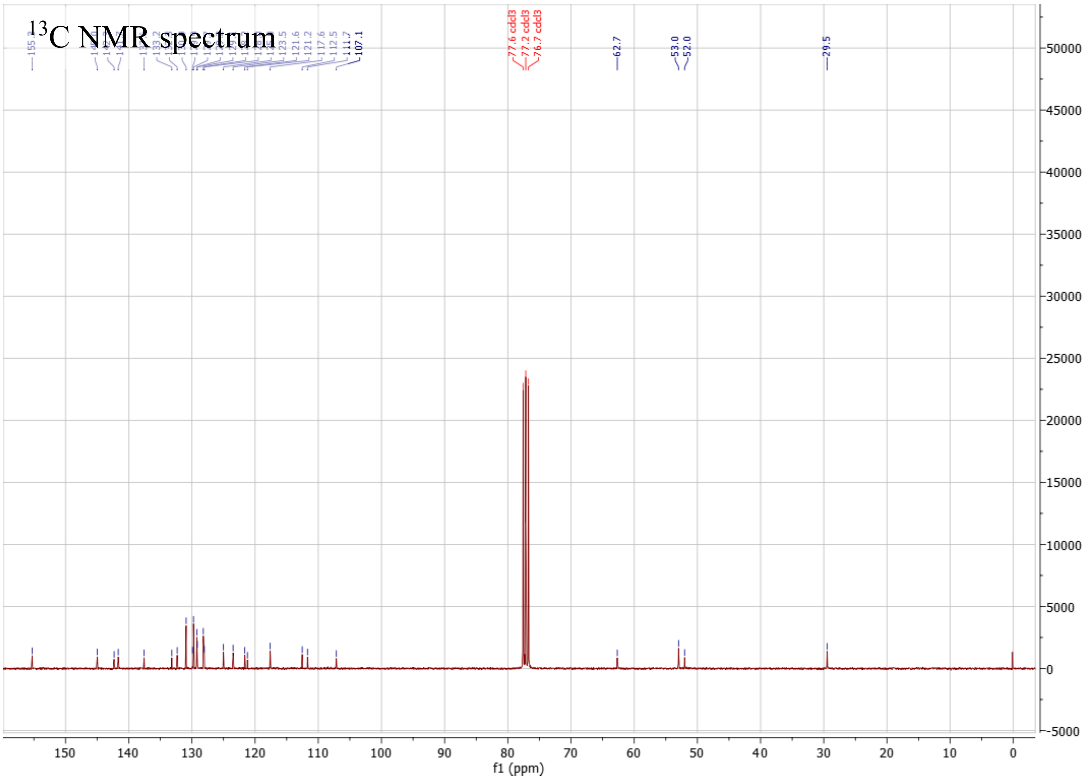

6-Amino-5-chloro-1-{1-[4-(3-phenylimidazo[1,2-*a*]pyridin-2-yl)benzyl]piperidin-4-yl}-1,3-dihydro-2*H*-benzo[*d*]imidazol-2-one (61).

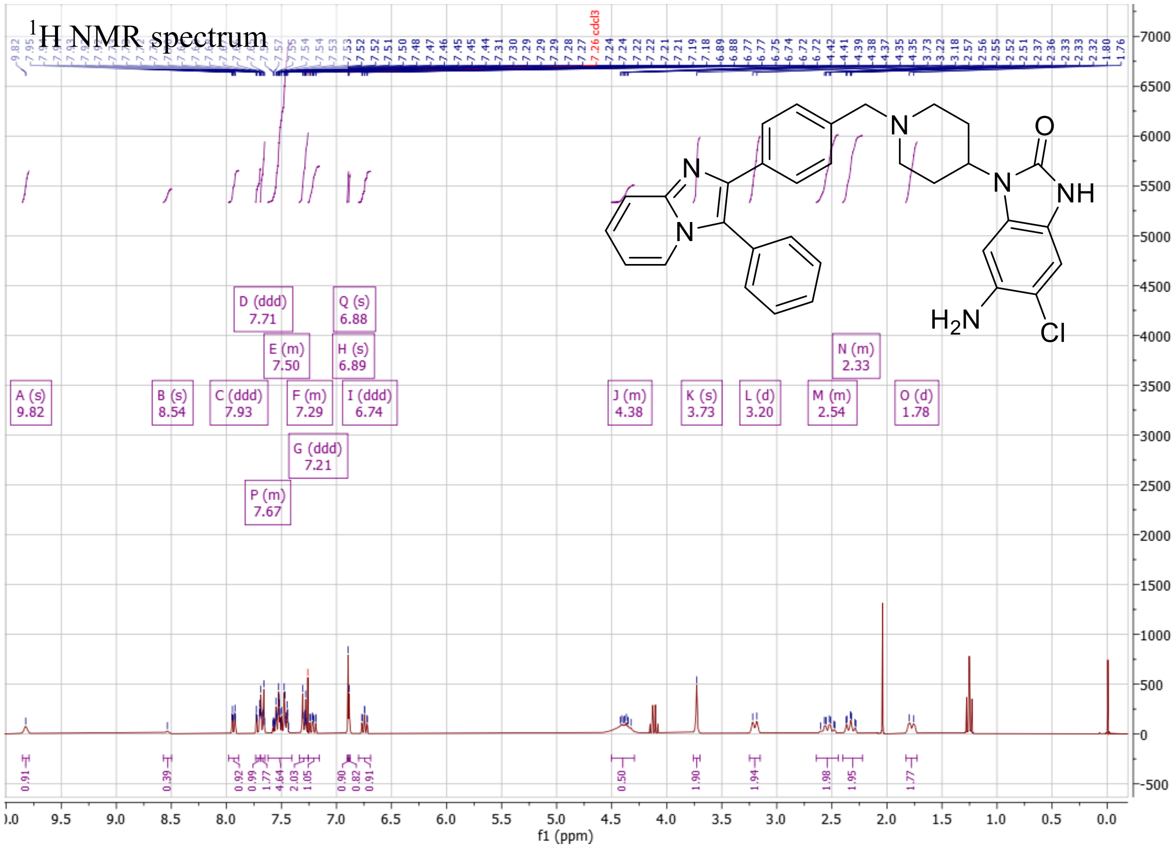

***N*-(6-Chloro-2-oxo-3-{1-[4-(3-phenylimidazo[1,2-*a*]pyridin-2-yl)benzyl]piperidin-4-yl}-2,3-dihydro-1*H*-benzo[*d*]imidazol-5-yl)acrylamide (12).**

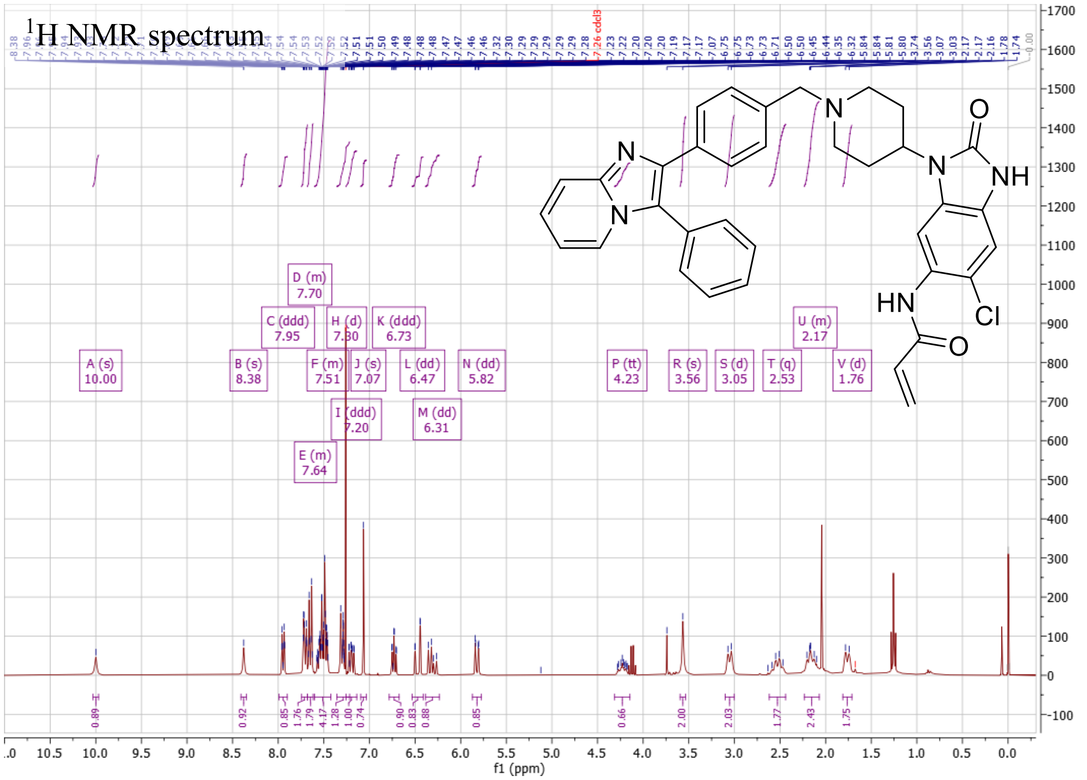

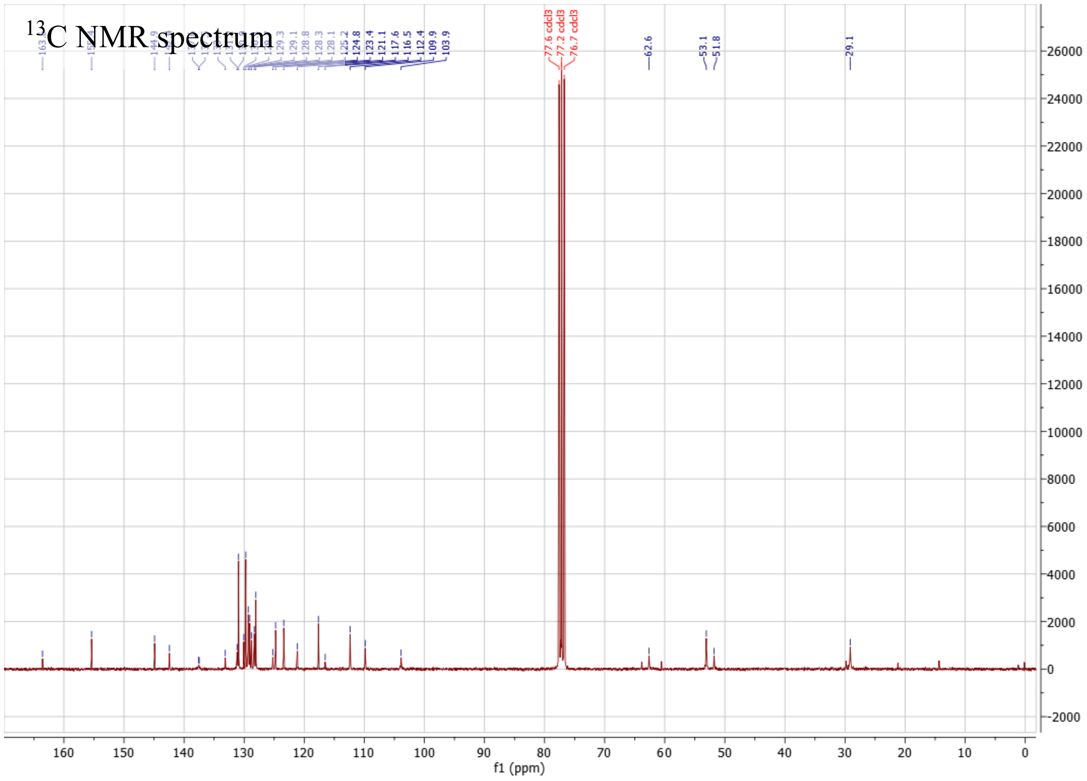

2-{4-[(4-Azidopiperidin-1-yl)methyl]phenyl}-3-phenyl-1,6-naphthyridin-5(6*H*)-one (63).

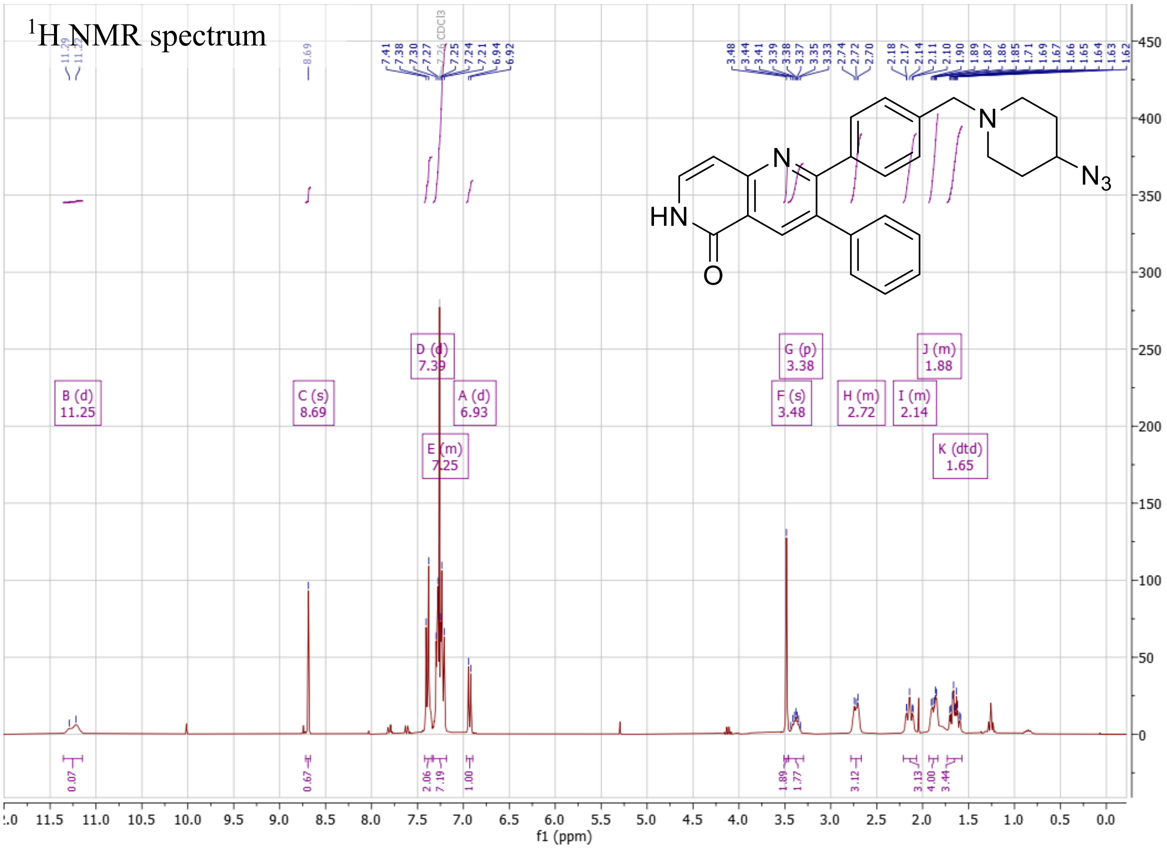

***N*-[3-(1-{1-[4-(5-Oxo-3-phenyl-5,6-dihydro-1,6-naphthyridin-2-yl)benzyl]piperidin-4-yl}-1*H*-1,2,3-triazol-4-yl)phenyl]acrylamide (13).**

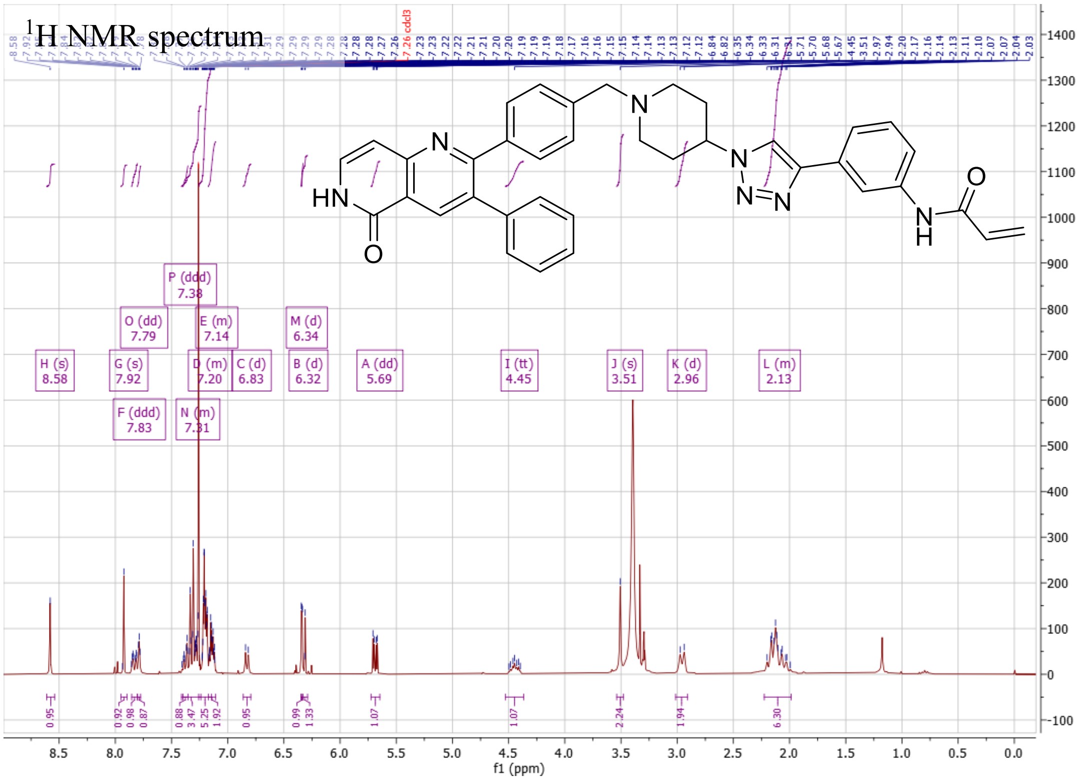

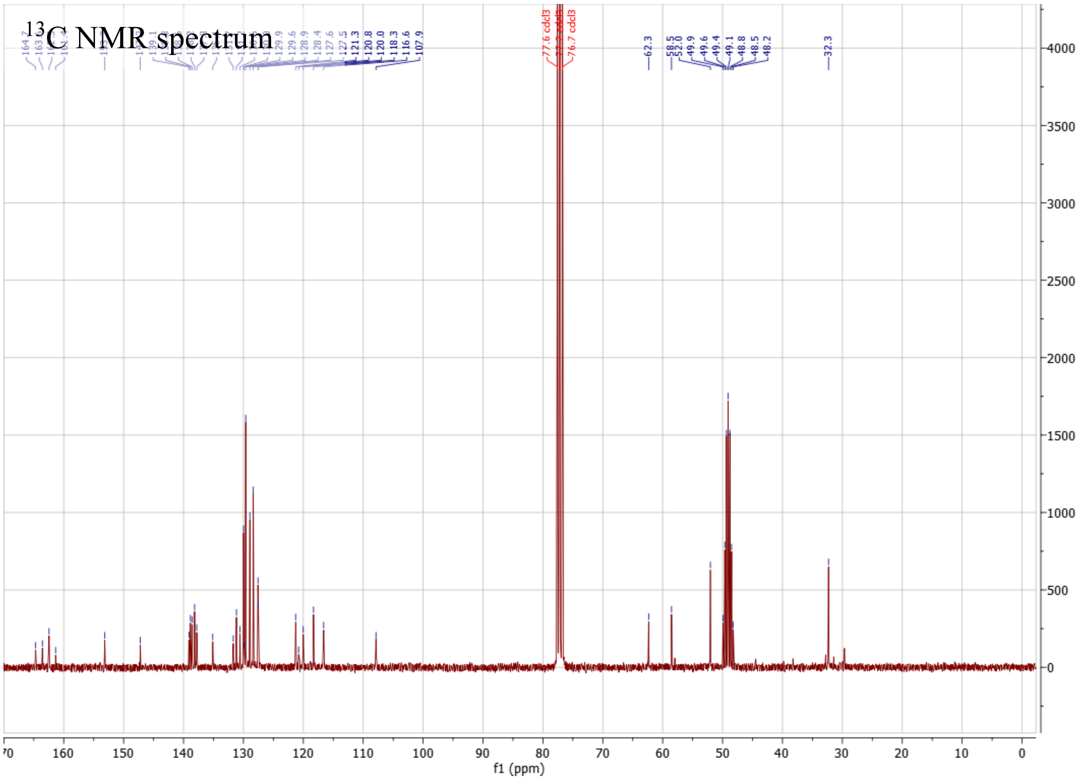

2D NMR ASSIGNMENTS FOR SELECT TARGET COMPOUNDS

Compound **7**

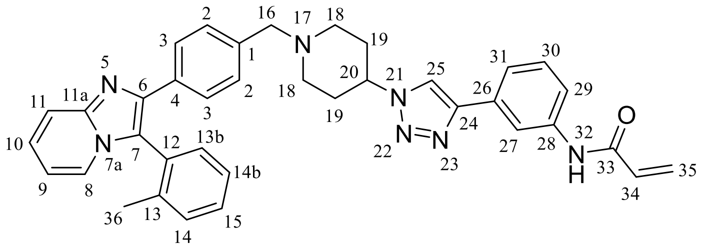

7

Atom #	^{13}C shift (ppm)	^{1}H shift (ppm)	COSY (ppm)	HMBC (ppm)
**1**	137.6	-	-	7.63 (3), 3.48 (16)
**2**	129.1	7.20, *d*, *J* = 7.8 Hz, 2H	7.63 (3), 3.48 (16)	7.20 (2), 3.48 (16)
**3**	127.3	7.63, *d*, *J* = 7.8 Hz, 2H	7.20 (2), 3.48 (16)	7.20 (2), 7.63 (3)
**4**	133.2	-	-	7.20 (2)
**6**	141.9	-	-	7.63 (3)
**7**	120.4	-	-	7.33 (13b)
**8**	123.5	7.56, *d*, *J* = 6.8 Hz, 1H	6.73 (9), 7.21 (10), 7.73 (11)	6.73 (9), 7.21 (10)
**9**	112.6	6.73, *app. t, dd*, *J* = 6.7, 6.7 Hz, 1H	7.56 (8), 7.21 (10), 7.73 (11)	7.56 (8), 7.73 (11)
**10**	124.9	7.21 [7.24 – 7.18], *m*, 1H	7.56 (8), 6.73 (9), 7.73 (11)	7.56 (8), 6.73 (9)
**11**	117.5	7.73, *d*, *J* = 9.0 Hz, 1H	7.56 (8), 6.73 (9), 7.21 (10)	6.73 (9)
**11a**	144.8	-	-	7.56 (8), 7.21 (10), 7.73 (11)
**12**	129.1	-	-	7.33 (13b), 7.42 (14), 2.00 (36)
**13**	131.9	-	-	7.42 (14)
**13b**	139.2	7.33 [7.38 – 7.30], *m*, 1H	7.42 (14b)	7.42 (14b), 2.00 (36)
**14**	131.1	7.42 [7.47 – 7.39], *m*, 1H	7.33 (15), 2.00 (36)	2.00 (36)
**14b**	129.9	7.42 [7.47 – 7.39], *m*, 1H	7.33 (15)	7.33 (15)
**15**	127.1	7.33 [7.38 – 7.30], *m*, 1H	7.42 (14/14b), 2.00 (36)	7.42 (14)

Atom #	^{13}C shift (ppm)	^{1}H shift (ppm)	COSY (ppm)	HMBC (ppm)
**16**	62.5	3.48, *s*, 2H	7.20 (2), 7.63 (3)	7.20 (2), 2.14 (18)
**18**	52.2	2.96, *d*, *J* = 11.4 Hz, 2H	2.14 (18/19), 2.04 (19), 4.44 (20)	3.48 (16), 2.14 (19), 2.04 (19)
		2.14 [2.18 – 2.11], *m*, 2H	2.96 (18), 2.04 (19), 4.44 (20)	
**19**	32.8	2.14 [2.18 – 2.11], *m*, 2H	2.96 (18), 2.04 (19), 4.44 (20)	3.48 (16), 2.14 (18), 4.44 (20)
		2.04 [2.08 – 2.01], *m*, 2H	2.96 (18), 2.14 (18/19), 4.44 (20)	
**20**	58.5	4.44, *app. tt, dddd*, *J* = 11.7, 11.7, 4.2, 4.2 Hz, 1H	2.96 (18), 2.14 (18/19), 2.04 (19), 7.75 (25)	2.14 (18/19), 2.04 (19)
**24**	147.2	-	-	7.75 (25), 8.05 (27), 7.59 (31)
**25**	117.8	7.75, *s*, 1H	4.44 (20)	4.44 (20), 7.59 (31)
**26**	138.7	-	-	7.33 (30)
**27**	117.2	8.05, *s*, 1H	7.63 (29), 7.59 (31)	7.59 (31)
**28**	131.5	-	-	7.33 (30)
**29**	119.7	7.63 [7.65 – 7.60], *m*, 1H	8.05 (27), 7.33 (30)	8.05 (27), 7.59 (31)
**30**	129.7	7.33 [7.38 – 7.30], *m*, 1H	7.63 (29), 7.59 (31)	-
**31**	121.7	7.59, *d*, *J* = 7.7 Hz, 1H	8.05 (27), 7.33 (30)	8.05 (27), 7.63 (29)
**32**	-	8.32, *s*, 1H	-	
**33**	164.0	-	-	6.33 (34), 6.42 (35), 5.71 (35)
**34**	131.4	6.33, *dd*, *J* = 16.8, 10.1 Hz, 1H	6.42 (35), 5.71 (35)	6.42 (35), 5.71 (35)
**35**	127.8	6.42, *d*, *J* = 16.8 Hz, 1H	6.33 (34), 5.71 (35)	6.33 (34)
		5.71, *d*, *J* = 10.1 Hz, 1H	6.33 (34), 6.42 (35)	
**36**	19.5	2.00, *s*, 3H	7.42 (14), 7.33 (15)	7.42 (14)

Compound **11**

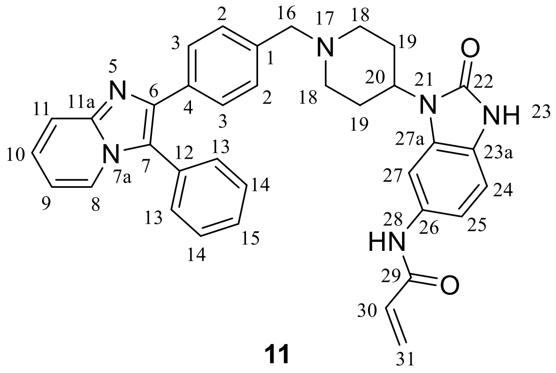

11

Atom #	^{13}C shift (ppm)	^{1}H shift (ppm)	COSY (ppm)	TOCSY (ppm)	HMBC (ppm)
**1**	136.6	-	-	-	7.63 (3), 3.57 (16)
**2**	129.6	7.26 [7.27 – 7.22], *m*, 2H	7.63 (3), 3.57 (16)	7.63 (3)	7.26 (2), 3.57 (16)
**3**	128.2	7.63, *d*, *J* = 8.2 Hz, 2H	7.26 (2), 3.57 (16)	7.26 (2)	7.26 (2)
**4**	133.3	-	-	-	7.26 (2)
**6**	142.2	-	-	-	7.63 (3)
**7**	121.2	-	-	-	7.95 (8), 7.49 (14)
**8**	123.5	7.95, *app. dt, ddd*, *J* = 6.9, 1.2, 1.2 Hz, 1H	6.74 (9), 7.20 (10), 7.74 (11)	6.74 (9), 7.20 (10), 7.74 (11)	6.74 (9), 7.20 (10)
**9**	112.5	6.74, *app. td, ddd*, *J* = 6.9, 6.8, 1.1 Hz, 1H	7.95 (8), 7.20 (10), 7.74 (11)	7.95 (8), 7.20 (10), 7.74 (11)	7.95 (8), 7.20 (10), 7.74 (11)
**10**	125.0	7.20, *ddd*, *J* = 9.1, 6.8, 1.2 Hz, 1H	7.95 (8), 6.74 (9), 7.74 (11)	7.95 (8), 6.74 (9), 7.74 (11)	7.95 (8)
**11**	117.6	7.74 [7.78 – 7.72], *m*, 1H	7.95 (8), 6.74 (9), 7.20 (10)	7.95 (8), 6.74 (9), 7.20 (10)	7.95 (8), 6.74 (9)
**11a**	145.0	-	-	-	7.95 (8), 7.20 (10), 7.74 (11)
**12**	129.8	-	-	-	7.49 (13)
**13**	129.7	7.49 [7.59 – 7.40], *m*, 2H	-	-	7.63 (3)
**14**	130.9	7.49 [7.59 – 7.40], *m*, 2H	-	-	7.49 (15)
**15**	129.1	7.49 [7.59 – 7.40], *m*, 1H	-	-	7.49 (14)
**16**	62.5	3.57, *s*, 2H	7.26 (2), 7.63 (3)	-	7.26 (2)

Atom #	^{13}C shift (ppm)	^{1}H shift (ppm)	COSY (ppm)	TOCSY (ppm)	HMBC (ppm)
**18**	53.0	3.02, *d*, *J* = 11.0 Hz, 2H	2.15 (18), 1.71 (19), 2.48 (19), 4.27 (20)	2.15 (18), 1.71 (19), 2.48 (19), 4.27 (20)	3.57 (16)
		2.15 [2.22 – 2.08], *m*, 2H	3.02 (18), 1.71 (19), 2.48 (19)	3.02 (18), 1.71 (19), 2.48 (19), 4.27 (20)	
**19**	28.9	2.48 [2.56 – 2.38], *m*, 2H	3.02 (18), 2.15 (18), 1.71 (19), 4.27 (20)	3.02 (18), 2.15 (18), 1.71 (19), 4.27 (20)	-
		1.71, *d*, *J* = 12.3 Hz, 2H	3.02 (18), 2.15 (18), 2.48 (19), 4.27 (20)	3.02 (18), 2.15 (18), 2.48 (19), 4.27 (20)	
**20**	50.9	4.27 [4.37 – 4.16], *m*, 1H	3.02 (18), 2.48 (19), 1.71 (19)	3.02 (18), 2.15 (18), 2.48 (19), 1.71 (19)	-
**22**	155.3	-	-	-	9.99 (23)
**23**	-	9.99, *s*, 1H	7.72 (27)	-	
**23a**	129.5	-	-	-	9.99 (23), 6.77 (24)
**24**	109.5	6.77, *d*, *J* = 8.4 Hz, 1H	7.11 (25)	7.11 (25), 7.72 (27)	-
**25**	113.9	7.11, *br d*, *J* = 8.4 Hz, 1H	6.77 (24), 7.72 (27)	6.77 (24)	7.72 (27)
**26**	125.0	-	-	-	7.11 (25), 7.72 (27)
**27**	103.0	7.72, *s*, 1H	9.99 (23), 7.11 (25), 8.46 (28)	6.77 (24)	7.11 (25), 8.46 (28)
**27a**	132.3	-	-	-	6.77 (24), 7.72 (27)
**28**	-	8.46, *s*, 1H	7.72 (27)	-	
**29**	164.0	-	-	-	8.46 (28), 6.30 (30), 6.40 (31), 5.65 (31)
**30**	131.5	6.30, *dd*, *J* = 16.9, 9.6 Hz, 1H	6.40 (31), 5.65 (31)	6.40 (31), 5.65 (31)	6.40 (31)
**31**	127.4	6.40, *dd*, *J* = 16.9, 2.1 Hz, 1H	6.30 (30)	6.30 (30), 5.56 (31)	6.30 (30)

Atom #	^{13}C shift (ppm)	^{1}H shift (ppm)	COSY (ppm)	TOCSY (ppm)	HMBC (ppm)
		5.65, *dd*, *J* = 9.6, 2.1 Hz, 1H	6.30 (30)	6.30 (30), 6.40 (31)	

Compound **13**

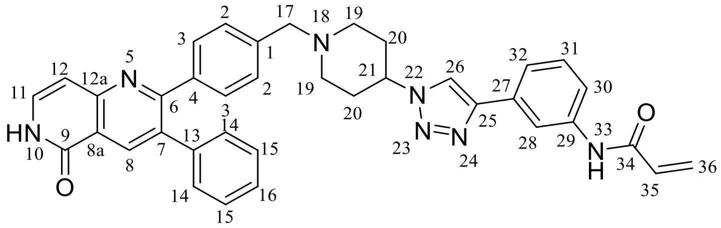

13

Atom #	^{13}C shift (ppm)	^{1}H shift (ppm)	COSY (ppm)	HMBC (ppm)
**1**	138.2	-	-	7.31 (3), 3.51 (17)
**2**	128.9	7.20 [7.26 – 7.15], *m*, 2H	7.31 (3), 3.51 (17)	7.31 (3), 3.51 (17)
**3**	130.0	7.31 [7.41 – 7.24], *m*, 2H	7.20 (2) , 3.51 (17)	-
**4**	138.6/135.1	-	-	7.20 (2/14)
**6**	162.5	-	-	7.31 (3), 8.58 (8)
**7**	138.8/138.6/135.1	-	-	7.20 (2/14), 8.58 (8)
**8**	137.8	8.58, *s*, 1H	6.83 (12)	-
**8a**	120.8	-	-	6.83 (12)
**9**	163.6	-	-	8.58 (8), 7.31 (11)
**11**	131.7	7.31 [7.41 – 7.24], *m*, 1H	6.83 (12)	6.83 (12)
**12**	107.9	6.83, *d*, *J* = 7.4 Hz, 1H	8.58 (8), 7.31 (11)	-
**12a**	153.2	-	-	8.58 (8), 7.31 (11)
**13**	138.8/138.6/135.1	-	-	7.20 (2/14), 8.58 (8)
**14**	128.4	7.20 [7.26 – 7.15], *m*, 2H	7.14 (15)	7.20 (16)
**15**	129.6	7.14 [7.17 – 7.11], *m*, 2H	7.20 (14/16)	7.20 (16)
**16**	127.6	7.20 [7.26 – 7.15], *m*, 1H	7.14 (15)	7.14 (15)
**17**	62.3	3.51, *s*, 2H	7.20 (2), 7.31 (3)	7.20 (2)
**19**	52.0	2.96, d, *J* = 10.6 Hz, 2H	2.13 (19/20), 4.45 (21)	3.51 (17)
		2.13 [2.23 – 1.99], *m*, 2H	2.96 (19), 4.45 (21)	
**20**	32.3	2.13 [2.23 – 1.99], *m*, 4H	2.96 (19), 4.45 (21)	-

Atom #	^{13}C shift (ppm)	^{1}H shift (ppm)	COSY (ppm)	HMBC (ppm)
**21**	58.5	4.45 [4.43 – 4.37], *m*, 1H	2.96 (19), 2.13 (19/20)	-
**25**	147.2	-	-	7.92 (26)
**26**	118.3	7.92, *s*, 1H	-	-
**27**	139.1/130.6	-	-	7.31 (31)
**28**	116.6	7.79, *app. t, dd*, *J* = 1.7, 1.7 Hz, 1H		7.38 (32)
**29**	139.1/130.6	-	-	7.31 (31)
**30**	120.0	7.83, *app. dt, ddd*, *J* = 8.1, 1.7, 1.5 Hz, 1H	7.31 (31)	7.79 (28), 7.38 (32)
**31**	129.9	7.31 [7.41 – 7.24], *m*, 1H	7.83 (30), 7.38 (32)	-
**32**	121.3	7.38, *app. dt, ddd*, *J* = 7.7, 1.7, 1.5 Hz, 1H	7.31 (31)	7.79 (28)
**34**	164.7	-	-	6.32 (35), 5.69 (36)
**35**	131.2	6.32, *d*, *J* = 8.6 Hz, 1H	6.34 (36), 5.69 (36)	6.34 (36)
**36**	127.5	6.34, *d*, *J* = 3.1 Hz, 1H	6.32 (35), 5.69 (36)	-
		5.69, *dd*, *J* = 8.6, 3.1 Hz, 1H	6.32 (35), 6.34 (36)	

LC-MS TRACES FOR PURITY

Compound **5**, Purity 95.4%.

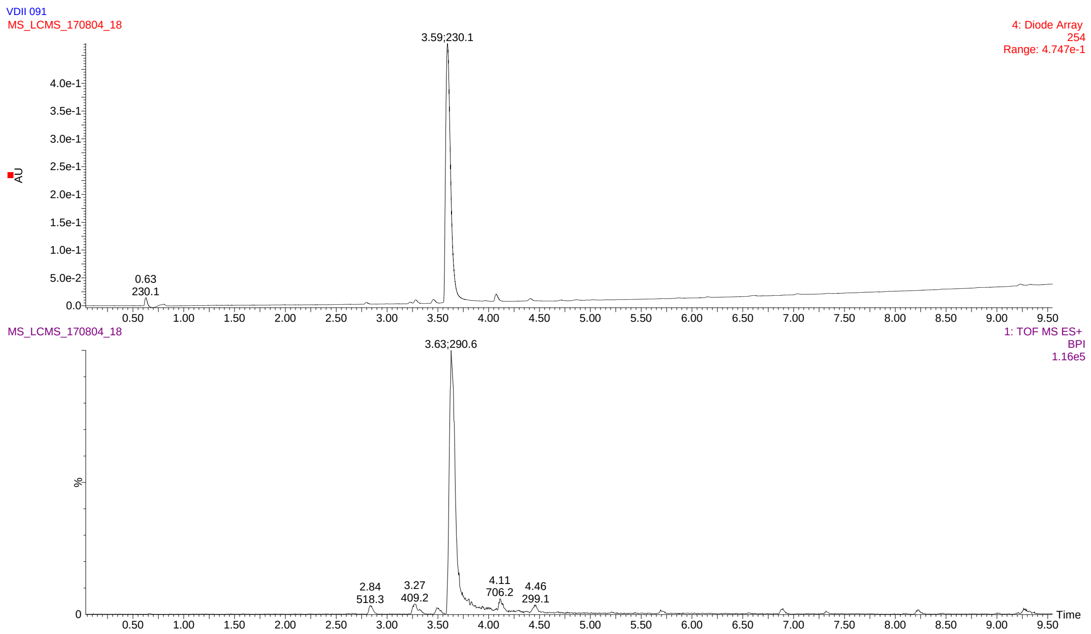

Compound **6**, Purity 98.8%.

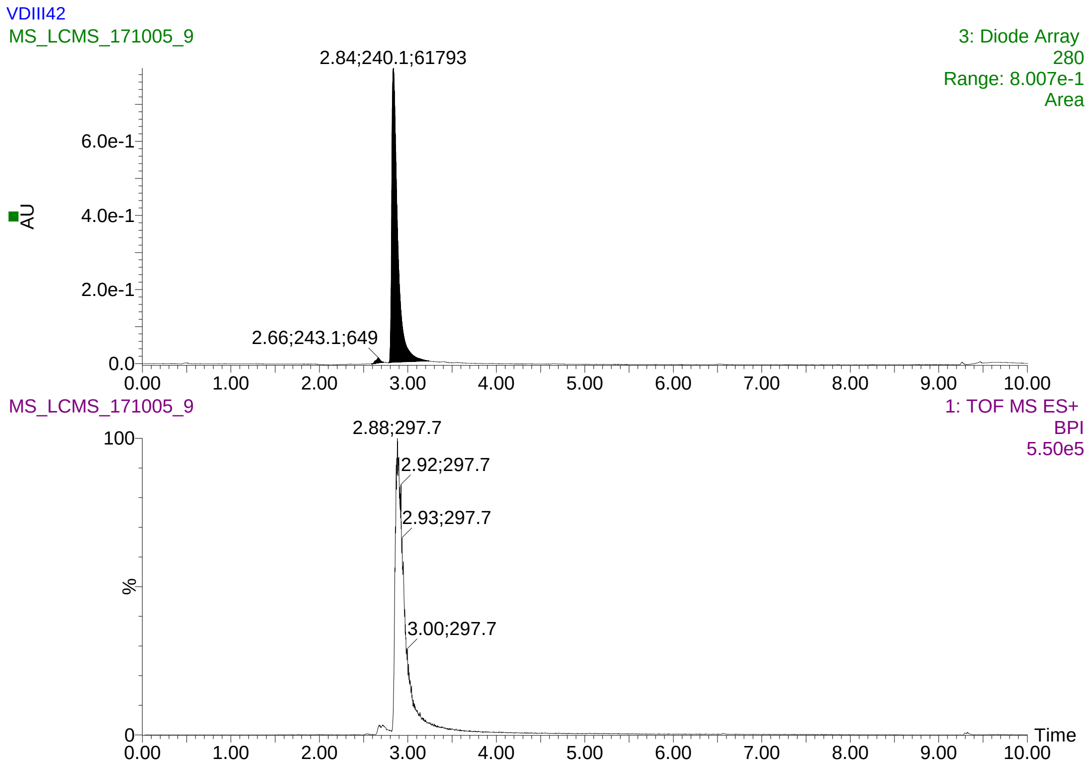

Compound **7**, Purity 99.9%.

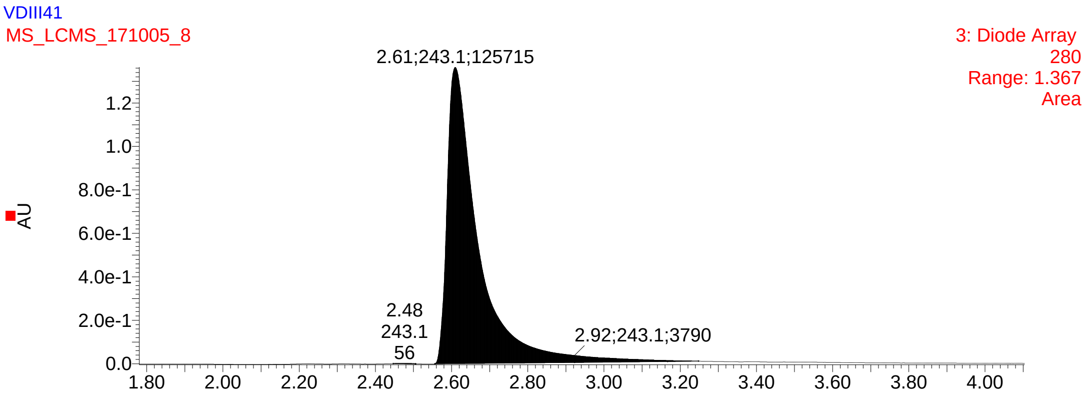

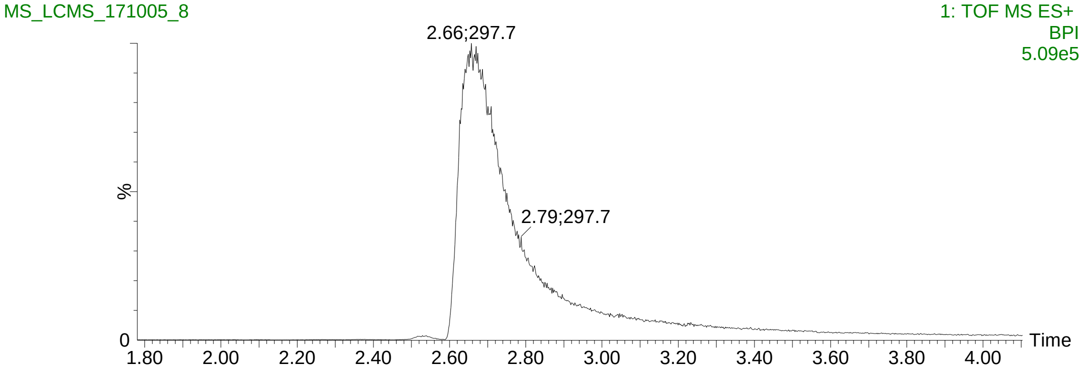

Compound **8**, Purity 92.4%.

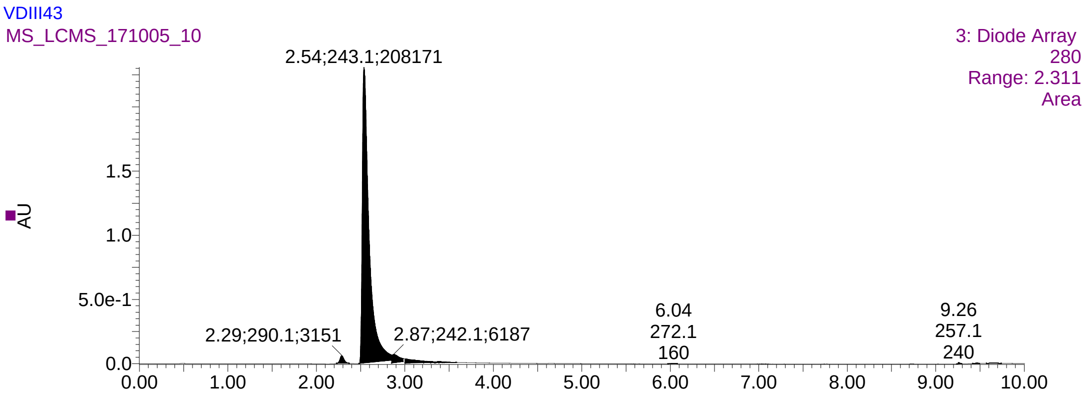

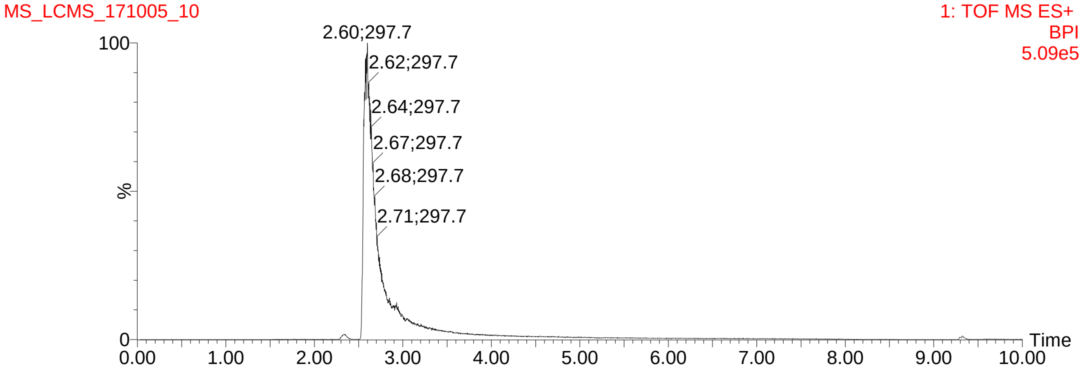

Compound **9**, Purity 93.0%.

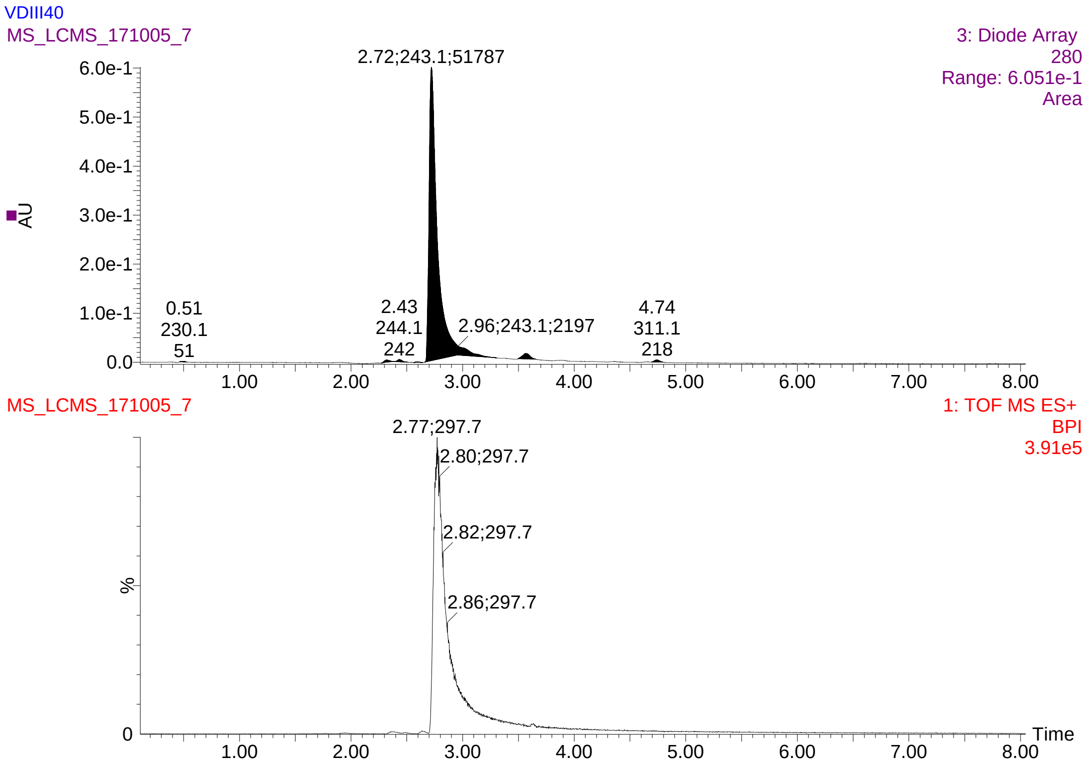

Compound **10**, Purity ~98%.

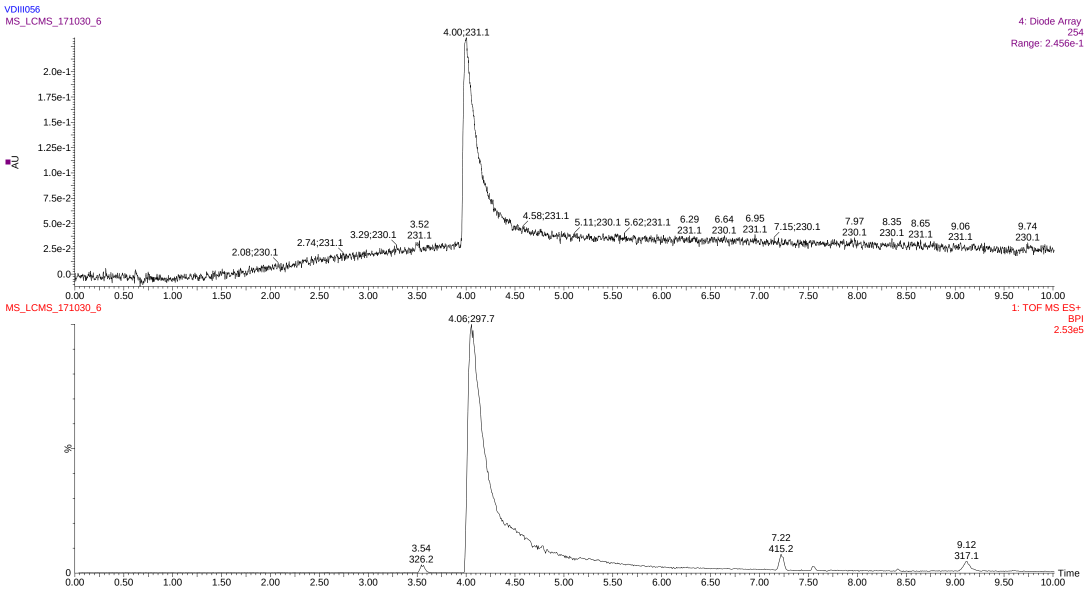

Compound **11**, Purity ~98%.

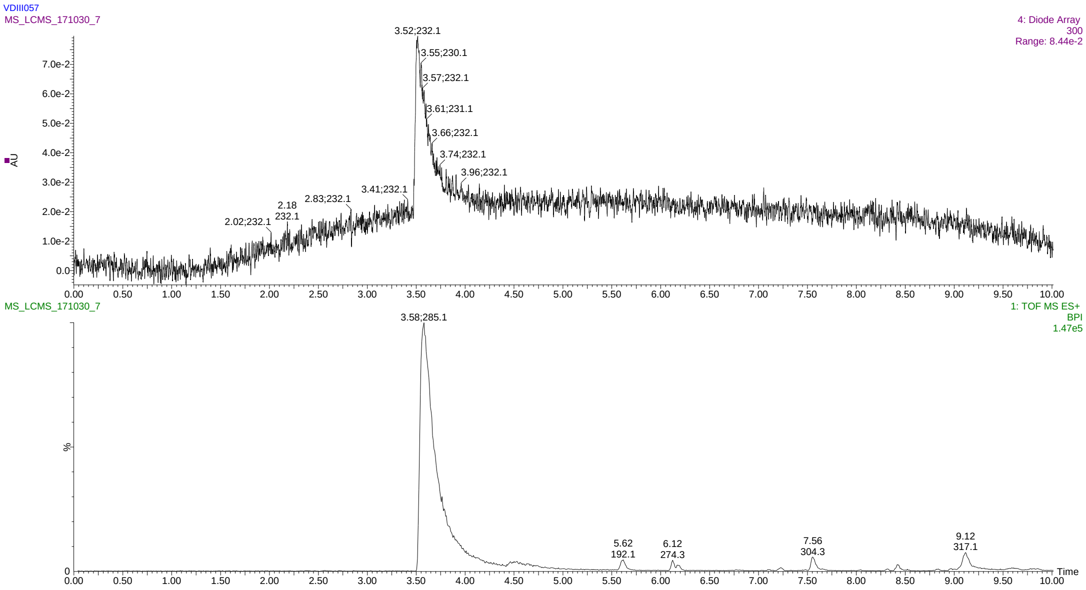

Compound **12**, Purity 99.5%.

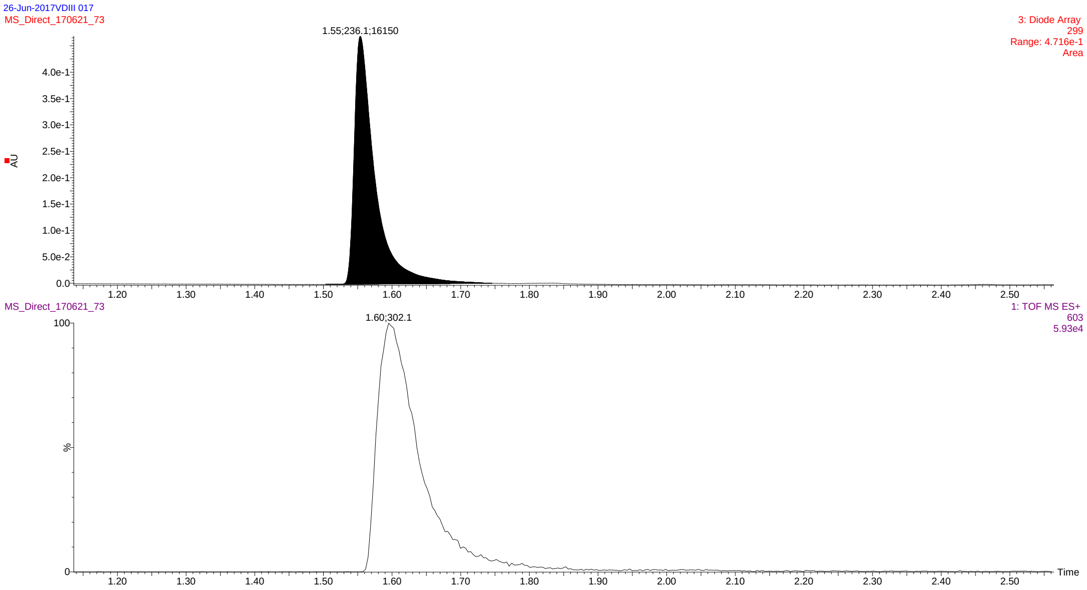

Compound **13**, Purity 88.8%.

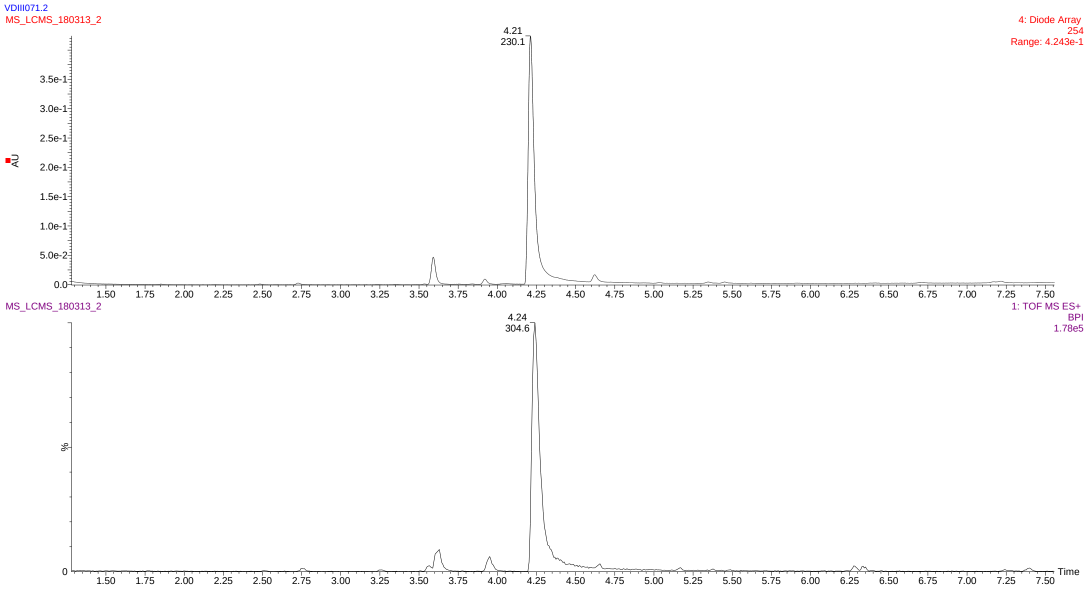

4. Molecular Modelling

Molecular modelling was carried out using the Schrödinger suite (2020-4). The Akt receptor 6HHG was obtained from the PDB and prepared using the standard protein preparation workflow, including adding missing side chains if required. All water molecules were deleted with the exception of HOH619 which forms a key bridging interaction in the pocket. Covalent docking was carried out using the Schrödinger covalent docking workflow selecting Cys310 as the reactive residue and a Michael addition reaction type. No constraints were employed.

5. References

(1) Fang, Z.; Simard, J. R.; Plenker, D.; Nguyen, H. D.; Phan, T.; Wolle, P.; Baumeister, S.; Rauh, D. Discovery of Inter-Domain Stabilizers - A Novel Assay System for Allosteric Akt Inhibitors. *ACS Chem. Biol.* **2015**, *10*, 279–288.

(2) Weisner, J.; Gontla, R.; van der Westhuizen, L.; Oeck, S.; Ketzer, J.; Janning, P.; Richters, A.; Mühlenberg, T.; Fang, Z.; Taher, A.; *et al.* Covalent-Allosteric Kinase Inhibitors. *Angew. Chem., Int. Ed.* **2015**, *54*, 10313–10316.

(3) Uhlenbrock, N.; Smith, S.; Weisner, J.; Landel, I.; Lindemann, M.; Le, T. A.; Hardick, J.; Gontla, R.; Scheinpflug, R.; Czodrowski, P.; *et al.* Structural and Chemical Insights into the Covalent-Allosteric Inhibition of the Protein Kinase Akt. *Chem. Sci.* **2019**, *10*, 3573–3585.

(4) Drexler, C. HTRF® KinEASE™: A New Solution for Screening Serine-Threonine Kinases. *Nat. Methods* **2006**, *3*, i–ii.

(5) Quambusch, L.; Landel, I.; Depta, L.; Weisner, J.; Uhlenbrock, N.; Müller, M. P.; Glanemann, F.; Althoff, K.; Siveke, J. T.; Rauh, D. Covalent-Allosteric Inhibitors to Achieve Akt Isoform-Selectivity. *Angew. Chem., Int. Ed.* **2019**, *58* (52), 18823–18829. https://doi.org/10.1002/anie.201909857.

(6) Krippendorff, B.-F.; Neuhaus, R.; Lienau, P.; Reichel, A.; Huisinga, W. Mechanism-Based Inhibition: Deriving Ki and Kinact Directly from Time-Dependent IC_{50} Values. *J. Biomol. Screening* **2009**, *14*, 913–923.

(7) Weisner, J.; Landel, I.; Reintjes, C.; Uhlenbrock, N.; Trajkovic-Arsic, M.; Dienstbier, N.; Hardick, J.; Ladigan, S.; Lindemann, M.; Smith, S.; *et al.* Preclinical Efficacy of Covalent-Allosteric AKT Inhibitor Borussertib in Combination with Trametinib in KRAS-Mutant Pancreatic and Colorectal Cancer. *Cancer Res.* **2019**, *79*, 2367-2378.

(8) Technical Bulletin: CellTiter-Glo® Luminescent Cell Viability Assay, Promega Corporation, United States https://worldwide.promega.com/products/cell-health-assays/cell-viability-and-cytotoxicity-assays/celltiter_glo-luminescent-cell-viability-assay/?catNum=G7570 (accessed Apr 5, 2019).
